# Regioselective (thio)carbamoylation of 2,7-di-*tert*-butylpyrene at the 1-position with iso(thio)cyanates

**DOI:** 10.3762/bjoc.13.102

**Published:** 2017-05-29

**Authors:** Anna Wrona-Piotrowicz, Marzena Witalewska, Janusz Zakrzewski, Anna Makal

**Affiliations:** 1 Department of Organic Chemistry, Faculty of Chemistry, University of Łódź, Tamka 12, 91-403 Łódź, Poland; 2 University of Warsaw, Biological and Chemical Research Center, Żwirki i Wigury 101, 02-089 Warszawa, Poland

**Keywords:** amide, Friedel–Crafts, isocyanate, isothiocyanate, pyrene, thioamide

## Abstract

It has been found that 2,7-di-*tert*-butylpyrene reacts with aliphatic iso(thio)cyanates in the presence of trifluoromethanesulfonic acid to exclusively afford the corresponding 1-substituted (thio)amides in high yields. For aromatic iso(thio)cyanates the reaction is less regioselective, although substitution at the 1-position prevails. For ethoxycarbonyl isothiocyanate, apart from the 1-substituted thioamide, 1,8-disubstituted thioamide and 2,7-di-*tert*-butylpyrene-1-carbonitrile are formed (especially at longer reaction times).

## Introduction

**Figure 1:**
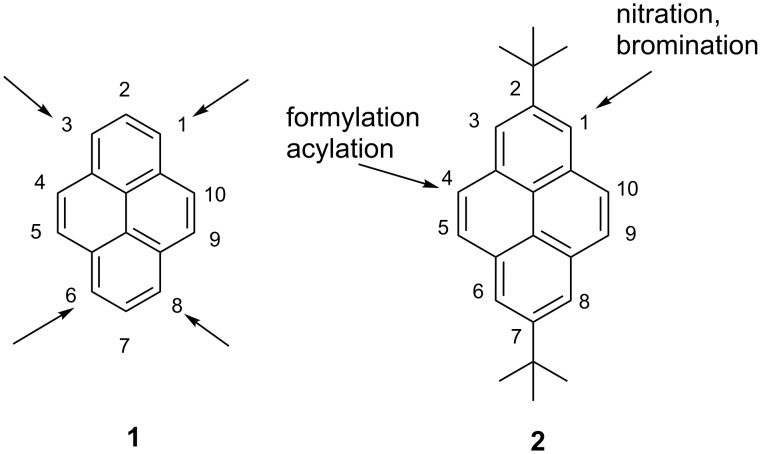
Sites of electrophilic attack in **1** and **2**.

Direct functionalization of pyrene **1** has attracted a great deal of attention in recent years because it is the most straightforward route to novel organic molecular materials for optoelectronic devices (OLEDs, field-effect transistors, fluorescent sensors, dye lasers, etc.) [1–7]. Since **1** is an electron-rich arene, aromatic electrophilic substitution seems to be the simplest method for this purpose, and a plethora of substituted pyrenes have been synthesized in this way [1]. As is shown in Figure 1, the most reactive in such reactions are positions 1, 3, 6 and 8 of **1**. However, Friedel–Crafts alkylation of **1** with an excess of sterically hindered *tert*-butyl chloride leads to 2,7-di-*tert*-butylpyrene (**2**) [8]. This compound, owing to the presence of two bulky and electron-donating *tert*-butyl groups, displays different reactivity towards electrophiles. It has been reported that nitration and bromination of **2** take place at the 1-position (however, the bromine atom in 1-bromopyrene can migrate into the 4-position in the presence of AlCl_3_) [8–9], whereas Friedel–Crafts acylation and Vilsmeier formylation take place at the 4-position [10].

**Scheme 1:**
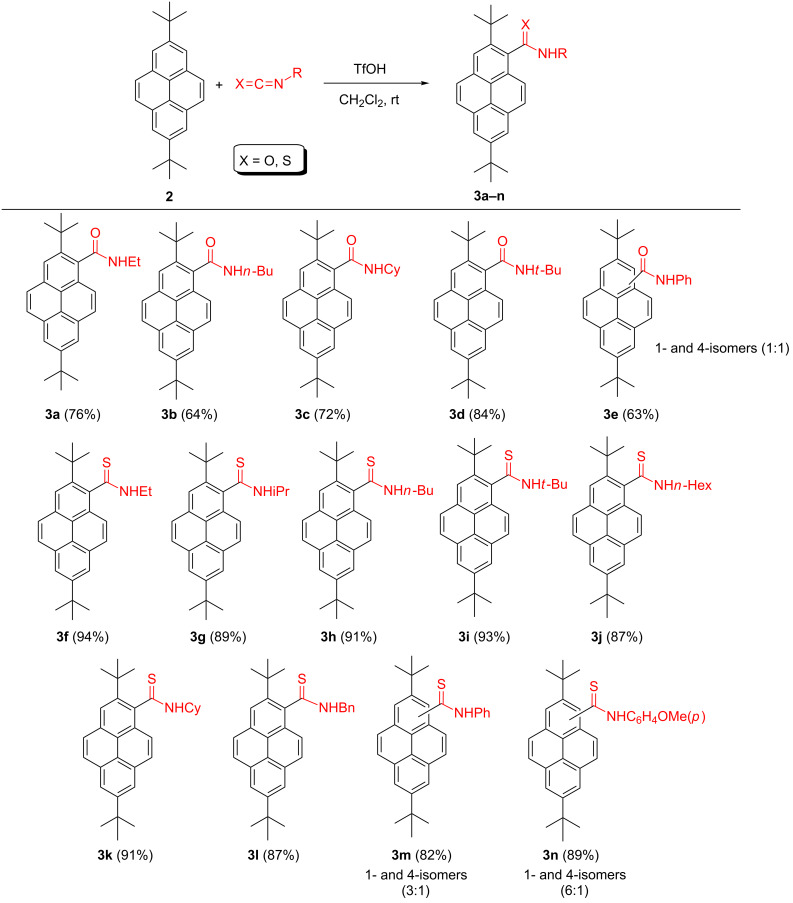
Triflic acid promoted reaction of **2** with iso(thio)cyanates.

We recently reported an efficient method for the synthesis of pyrene-1-carbothioamides via reaction of pyrene with isothiocyanates in the presence of trifluoromethanesulfonic (triflic) acid (TfOH) [11–12]. Since **2** was used as a starting material in the syntheses of various pyrenyl fluorophores exhibiting unique photophysical properties [4,10,13–20], we thought it would be of interest to study its reactivity in the above reaction (and to extend its scope for isocyanates). An additional reason for such a study was the fact that thioamides (and amides) are versatile starting materials in the syntheses of various products (especially heterocycles) [21–22]. Furthermore, aromatic amides undergo a variety of directed C–H bond functionalizations [23]. Herein we report that the reaction of **2** with aliphatic isocyanates and isothiocyanates proceeds regioselectively at the 1-position (Scheme 1).

## Results and Discussion

The reaction was carried out at room temperature in dichloromethane using 1.7 equiv of isocyanate or isothiocyanate and 3.3 equiv of TfOH. For the isocyanates, the reaction was completed in less than 5 min, whereas the reaction with isothiocyanates required ≈30 min for completion. For the aliphatic isocyanates or isothiocyanates it afforded exclusively C1-substituted (thio)amides (as deduced from the splitting pattern of the pyrenyl protons in their ^1^H NMR spectra, containing one pair of 1-proton doublets displaying a *meta*-coupling constant (≈2Hz, H6 and H8), two pairs of 1-proton doublets displaying *ortho*-coupling constants (≈7 Hz, H4, H5 and H9, H10) and one 1-proton singlet (H3).

Unexpectedly, the reaction of **2** with aromatic iso(thio)cyanates was less selective and led to inseparable mixtures of 1-subtituted (main components) and 4-substituted products **3e,m,n**. However, for the reaction with *p*-methoxyphenyl isothiocyanate we were able to obtain, by crystallization, the major product, C(1)-substituted thioamide **3n**, contaminated with only ≈5% of its C4-substituted counterpart. We tentatively explain this poorer regioselectivity by possible π–π interaction of pyrene with the arene ring of the protonated aromatic iso(thio)cyanate during formation of the Wheland complex, which may direct the electrophilic attack partly to the 4-position of the pyrene moiety.

To the best of our knowledge, the reaction reported here is the first example of a C1-selective substitution of **2** leading to the formation of a C–C bond.

The presence of two *tert*-butyl groups significantly increases the reactivity of **2** in comparison with **1**. A control experiment in which an equimolar mixture of **1** and **2** was treated in dichloromethane at rt with one equiv of *tert-*butyl isothiocyanate and 2 equiv of TfOH for 30 min resulted in practically exclusive formation of thioamide **3d** (the amount of the product of thiocarbamoylation of **1** was estimated as <2%).

**Scheme 2:**
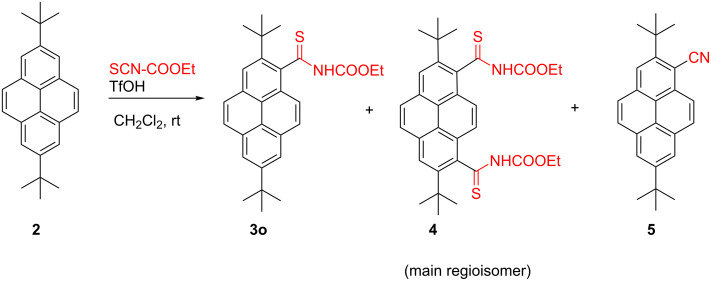
Triflic acid promoted reaction of **2** with ethoxycarbonyl isothiocyanate.

The reaction of **2** with ethoxycarbonyl isothiocyanate (2 equiv) in the presence of TfOH ran in a more complex way (Scheme 2, Table 1).

**Table 1:** Reaction of **2** with ethoxycarbonyl isothiocyanate (2 equiv) and TfOH (4 equiv) in CH_2_Cl_2_ at room temperature.

Entry	Reaction time	**3o** (%)	**4** (%)	**5** (%)

1	10 min	78	9	7

2	2 h	46	17	13

3	4 h	40	21	18

4	6 h	39	33	20

5	8 h	36	30	21

6	16 h	29	26	32

7	3 days	15	17	38

8	7 days	10	14	54
9	7days^a^	9	3	43
10	12 days	7	9	19

^a^3 Equivalents of isothiocyanate were used.

After 10 min, 1,8-bis-carbothioamide **4**, containing ≈20% of other regioisomers (9%) and nitrile **5** (7%) were isolated by column chromatography besides the expected **3o** (78%). The amounts of **4** and **5** significantly increased along with the reaction time (Table 1). The highest yield of **4** (33%) was found after 6 h, whereas a 7-day reaction afforded **5** in a moderate (54%) isolated yield.

**Figure 2:**
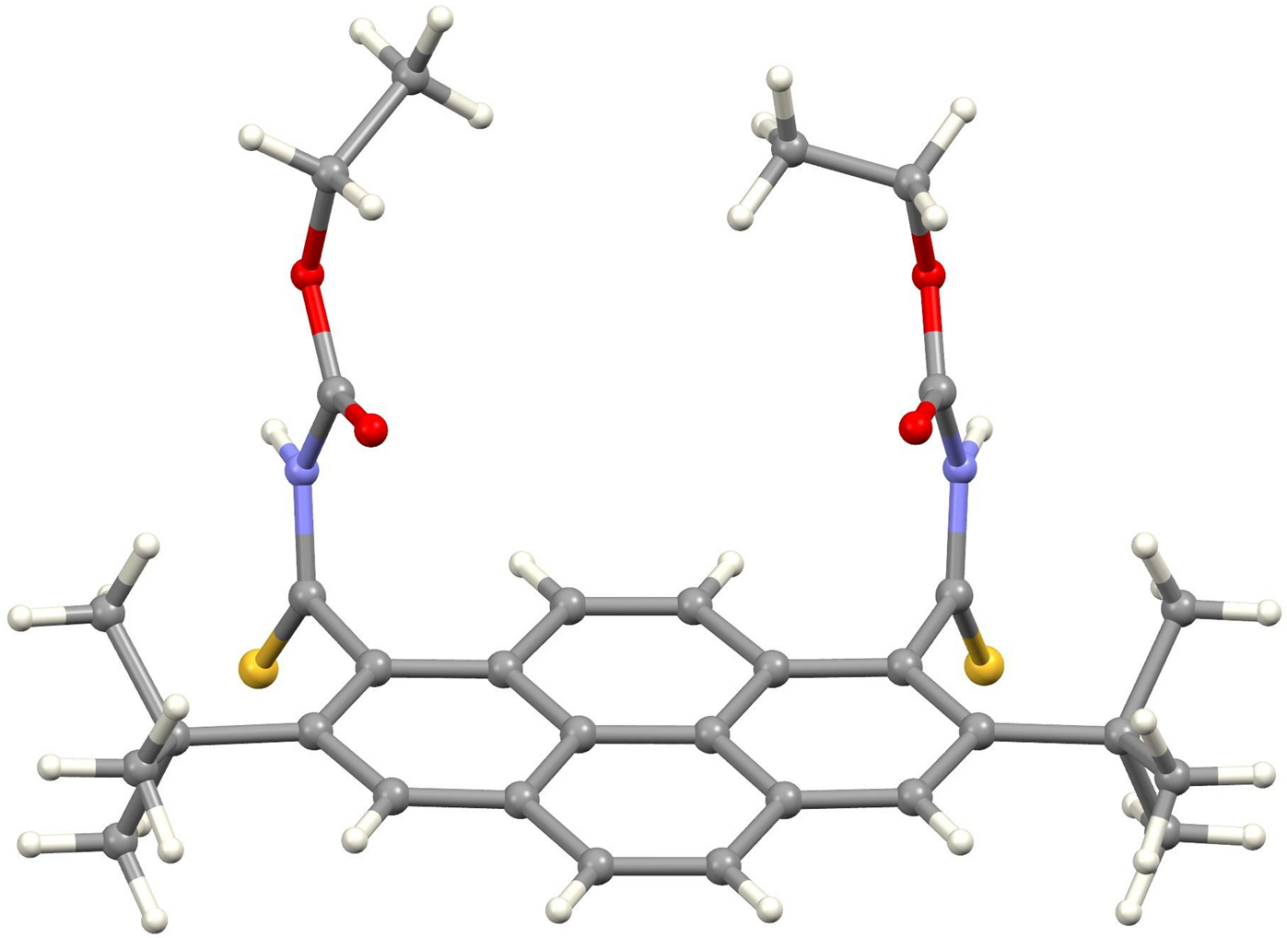
Molecular structure of **4**.

Attempts to separate the regioisomeric bis-thioamides failed, but repeated chromatography and recrystallization from dichloromethane/hexane allowed isolation of the practically pure main regioisomer, 1,8-dithioamide **4**. Its structure was confirmed by single-crystal X-ray diffraction, which revealed a cisoidal conformation of this molecule in the crystal (Figure 2, for details, see Supporting Information File 1 and Supporting Information File 2). However, the ^1^H NMR spectrum of **4** at room temperature was more complex than expected and contained broadened signals, suggesting the presence of other rotamers in CDCl_3_ solution.

The mechanism of the formation of nitrile **5** in the reaction under study here is so far unclear. The control experiment showed that under reaction conditions the secondary thioamide **3o** undergoes transformation into **5**, but to our knowledge this reaction, which must involve cleavage of the C–N bond, has no precedent in the literature (in contrast to the well-known formation of nitriles from primary thioamides, involving formal elimination of H_2_S [24]).

**Scheme 3:**
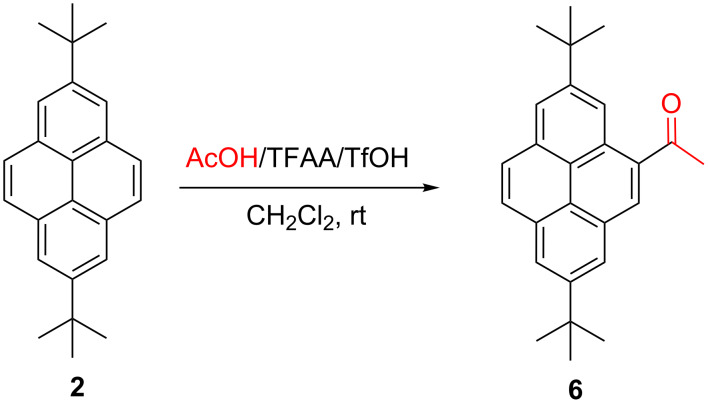
Friedel–Crafts acylation of **2**.

Encouraged by the above results, we decided to check whether TfOH-promoted Friedel–Crafts acylation of **2** will also occur at the 1-position. For this purpose, we examined the reaction of **2** with acetic acid and trifluoroacetic anhydride, (TFAA)/TfOH, according to our protocol used for the acylation of pyrene with alkynoic acids [25]. However, in this case we observed exclusive formation of C-4 substituted ketone **6** (Scheme 3).

In the ^1^H NMR spectrum of **6** we can see two pairs of doublets split with *J*_meta_ (1.8 Hz), assignable to H(1),H(3) and H(6),H(8) and a pair of doublets split with *J*_ortho_ (9 Hz) assignable to H(9),H(10 as well as a singlet (H5).

In our opinion, the observed difference in the regioselectivity of the (thio)carbamoylation and acylation of **2** may be due to different bulkiness of the reacting electrophile: the electrophilic center of the protonated iso(thio)cyanate is relatively unhindered and able to attack the electronically activated but sterically hindered pyrene 1-position, whereas the bulkier protonated acetyl trifluoroacetate (the postulated electrophile in the examined Friedel–Crafts acylation) attacks sterically the less hindered 4-position.

## Conclusion

We found that triflic acid-promoted (thio)carbamoylation of **2** with aliphatic iso(thio)cyanates occurs selectively at the 1- position, whereas Friedel–Crafts acylation promoted by this acid leads to 4-substitution. The reaction is less regioselective for aromatic iso(thio)cyanates. We believe that the described reaction opens up new synthetic routes to novel fluorophores having a sterically hindered pyrene framework.

## Experimental

### General

All reagents were purchased from Sigma-Aldrich and used without further purification. Solvents were purified before use by reported methods. Column chromatography was carried out on silica gel 60 (0.040–0.063 mm, 230–400 mesh, Fluka). ^1^H and ^13^C NMR spectra were recorded in CDCl_3_ at 600 MHz for ^1^H and 150 MHz for ^13^C at room temperature. Chemical shifts are reported in ppm and are referenced relative to solvent signals.

#### 2,7-Di-*tert-*butylpyrene-1-carboxamides. General procedure

Analogous as described in [12]. Isocyanate (1.2 mmol) and TfOH (348 μL, 4 mmol) were added to a solution of 2,7-di-*tert-*butylpyrene (**2**, 314 mg, 1 mmol) in CH_2_Cl_2_ (10 mL) at room temperature. After stirring for 5 min, the reaction mixture was poured into ice-water (50 mL) and extracted several times with CH_2_Cl_2_. The combined extracts were dried over anhydrous Na_2_SO_4_ and evaporated. The products were isolated by flash chromatography (eluent: CH_2_Cl_2_).

**2,7-Di-*tert*-butyl-*N*-ethylpyrene-1-carboxamide (3a)**. White solid (293 mg, 76%). Mp 258–259 °C; ^1^H NMR (600 MHz, CDCl_3_) δ 8.26 (s, 1H), 8.20 (d, *J* = 1.8 Hz, 1H), 8.18 (d, *J* = 1.8 Hz, 1H), 8.03 (m, 3H), 7.98 (d, *J* = 9.0 Hz, 1H), 5.87 (t, *J* = 5.4 Hz, 1H), 3.74 (m, 1H), 3.64 (m, 1H), 1.67 (s, 9H), 1.58 (s, 9H), 1.36 (t, *J* = 7.2 Hz, 3H); ^13^C NMR (150 MHz, CDCl_3_) δ 171.8, 149.2, 144.0, 131.3, 131.1, 130.9, 130.3, 128.7, 128.4, 128.2, 127.2, 124.4, 123.6, 122.7, 122.7, 122.4, 122.4, 37.1, 35.2, 35.1, 32.3, 31.9, 14.4; IR (KBr, cm^−1^): 3414, 3259, 2958, 1629, 1534, 1300, 1224, 880; anal. calcd. for C_27_H_31_NO: C, 84.11; H, 8.10; N, 3.63; O, 4.15; found: C, 84.07; H, 8.12; N, 3.71.

(For characterization data of other synthesized amides, see Supporting Information File 1).

#### 2,7-Di-*tert-*butylpyrene-1-carbothioamides. General procedure

Analogous as described in [12]. Isothiocyanate (1.2 mmol) and TfOH (348 μL, 4 mmol) were added to a solution of 2,7-di-*tert-*butylpyrene (314 mg, 1 mmol, ) in CH_2_Cl_2_ (10 mL) at room temperature. After stirring for 30 min, the reaction mixture was poured into ice-water (50 mL) and extracted several times with CH_2_Cl_2_. The combined extracts were dried over anhydrous Na_2_SO_4_ and evaporated. For the reaction with ethoxycarbonyl isothiocyanate, chromatography afforded two fractions. The less polar fraction contained monothioamide **3o** and nitrile **5**, whereas the more polar fraction contained dithioamides **4**. The separation of **3o** and **5** required the second chromatography with CH_2_Cl_2_/hexanes (1:1) as the eluent. The yields of **3o**, **4** and **5** are gathered in Table 1.

**2,7-Di-*tert*-butyl-*N*-ethylpyrene-1-carbothioamide (3f)**. White solid (378 mg, 94%). Mp 222–223 °C; ^1^H NMR (600 MHz, CDCl_3_) δ 8.28 (s, 1H), 8.19 (d, *J* = 1.2 Hz, 1H), 8.17 (d, *J* = 1.2 Hz, 1H), 8.08 (d, *J* = 9.6 Hz, 1H), 8.02 (d, *J* = 9.0 Hz, 1H), 8.01 (d, *J* = 9.0 Hz, 1H), 7.95 (d, *J* = 9.0 Hz, 1H), 7.59 (s, 1H), 4.09 (m, 1H), 3.96 (m, 1H), 1.72 (s, 9H), 1.58 (s, 9H), 1.43 (t, *J* = 7.2 Hz, 3H); ^13^C NMR (150 MHz, CDCl_3_) δ 203.4, 149.2, 142.5, 137.1, 131.0, 130.9, 130.3, 128.4, 128.0, 127.7, 127.2, 124.3, 124.2, 122.9, 122.7, 122.3, 41.0, 37.8, 35.2, 32.6, 31.9, 12.8; IR (KBr, cm^−1^): 3424, 3164, 2961, 1603, 1540, 1391, 1334, 1223, 881; anal. calcd. for C_27_H_31_NS: C, 80.75; H, 7.78; N, 3.49; S, 7.98; found: C, 80.70; H, 7.80; N, 3.51, S, 7.99.

**Ethyl (2,7-di-*tert*-butylpyrene-1-carbonothioyl)carbamate (3o).** Yellow solid. Mp 97–98 °C; ^1^H NMR (600 MHz, CDCl_3_) δ 9.82 (s, 1H), 8.33 (s, 1H), 8.19 (d, *J* = 13.2 Hz, 2H), 8.05 (s, 2H), 7.99 (m, 2H), 3.92 (q, *J* = 7.2 Hz, 2H), 1.74 (m, 9H), 1.59 (m, 9H), 0.97 (t, *J* = 7.2 Hz, 3H); ^13^C NMR (150 MHz, CDCl_3_) δ 211.0, 149.0, 148.9, 141.9, 134.9, 131.0, 130.8, 130.2, 128.4, 127.8, 127.4, 125.9, 124.4, 123.4, 122.7, 122.6, 122.5, 122.2, 63.0, 37.6, 35.2, 32.5, 31.9, 13.8; IR (KBr, cm^−1^): 3447, 3383, 2961, 2906, 2868, 1769, 1479, 1227, 1137, 1043, 881; anal. calcd. for C_28_H_31_NO_2_S: C, 75.47; H, 7.01; N, 3.14; O, 7.18; S, 7.20; found: C, 75.34; H, 7.18; N, 3.21, S, 7.14.

**Diethyl (2,7-di-*tert*-butyl**.**pyrene-1,8-dicarbonothioyl)carbamate (4).** Yellow solid. Mp 240–241 °C; ^1^H NMR (600 MHz, CDCl_3_) δ 9.75 (s, 1H), 9.72 (s, 1H), 8.28 (m, 2H), 8.02 (s, 2H), 7.95 (s, 2H), 3.90 (m, 4H), 1.69 (m, 18H), 0.97 (m, 3H), 0.88 (m, 3H). The ^13^C NMR spectrum was not measured due to limited solubility of this compound. IR (KBr, cm^−1^): 3432, 3385, 3162, 2964, 2910, 1773, 1492, 1228, 1168, 1149, 1110, 1038; anal. calcd. for C_32_H_34_N_2_O_4_S_2_: C, 66.64; H, 6.29; N, 4.86; O, 11.10; S, 11.12; found: C, 66.59; H, 6.33; N, 4.81, S, 11.03.

(For characterization data of other synthesized thioamides, see Supporting Information File 1).

**2,7-Di-*tert*-butylpyrene-1-carbonitrile (5).** Yellow solid. Mp 176–177 °C; ^1^H NMR (600 MHz, CDCl_3_) δ 8.50 (d, *J* = 9.0 Hz, 1H), 8.30 (d, *J* = 1.2 Hz, 1H), 8.28 (d, *J* = 1.2 Hz, 1H), 8.18 (d, *J* = 9.6 Hz, 1H), 8.17 (s, 1H), 8.14 (d, *J* = 9.0 Hz, 1H), 7.98 (d, *J* = 9.0 Hz, 1H), 1.81 (s, 9H), 1.62 (s, 9H); ^13^C NMR (150 MHz, CDCl_3_) δ 150.9, 149.9, 134.7, 133.4, 130.55, 130.53, 130.50, 130.0, 126.9, 124.0, 124.0, 123.9, 122.1, 121.9, 121.5, 119.8, 103.9, 36.2, 35.3, 31.8, 30.8; IR (KBr, cm^−1^): 2958, 2905, 2872, 2198, 1600, 1465, 1364, 1228, 879, 733; anal. calcd. for C_25_H_25_N: C, 88.45; H, 7.42; N, 4.13; found: C, 88.39; H, 7.51; N, 4.06.

**4-Acetyl-2,7-di-*tert*-butylpyrene (6).** 2,7-Di-*tert-*butylpyrene (**2**, 628 mg, 2 mmol) and triflic acid (174 μL, 2 mmol) were added at 0 °C to a solution of acetic acid (126 μL, 2.2 mmol) and trifluoroacetic anhydride (278 μL, 2 mmol) in CH_2_Cl_2_ (20 mL). The reaction mixture was warmed to room temperature and stirred for 2 h. After this time the reaction mixture was poured into ice-water (50 mL) and extracted several times with CH_2_Cl_2_. The combined extracts were dried over anhydrous Na_2_SO_4_ and evaporated. Flash chromatography (eluent: CH_2_Cl_2_) afforded the product. Yellow solid. (310 mg, 87%). Mp 121–122 °C; ^1^H NMR (600 MHz, CDCl_3_) δ 9.23 (d, *J* = 1.8 Hz, 1H), 8.59 (s, 1H), 8.29 (d, *J* = 1.2 Hz, 1H), 8.28 (d, *J* = 1.8 Hz, 1H), 8.24 (d, *J* = 1.2 Hz, 1H), 8.05 (d, *J* = 9.0 Hz, 1H), 8.01 (d, *J* = 9.0 Hz, 1H), 2.94 (s, 3H), 1.60 (s, 18H); ^13^C NMR (150 MHz, CDCl_3_) δ 201.9, 149.51, 149.07, 134.8, 132.3, 130.80, 130.78, 128.9, 128.3, 127.1, 126.7, 124.5, 123.9, 123.6, 123.5, 122.9, 121.9, 35.5, 35.2, 31.93, 31.88, 29.9; IR (KBr, cm^–1^): 2958, 2901, 2870, 1666, 1601, 1391, 1225, 1200, 892; anal. calcd. for C_26_H_28_O: C, 87.60; H, 7.98; O, 4.48; found: C, 87.57; H, 8.05.

## Supporting Information

File 1 Characterization data, copies of ^1^H, ^13^C NMR and IR spectra for synthesized compounds, and crystallographic structure and refinement data of **4**.

File 2 Crystallographic data for **4**.

## References

[1] Feng X, Hu J-Y, Redshaw C, Yamato T (2016). Chem – Eur J.

[2] Figueira-Duarte T M, Müllen K (2011). Chem Rev.

[3] Casas-Solvas J M, Howgego J D, Davis A P (2014). Org Biomol Chem.

[4] Mateo-Alonso A (2014). Chem Soc Rev.

[5] Niko Y, Cho Y, Kawauchi S, Konishi G-i (2014). RSC Adv.

[6] Piotrowicz M, Zakrzewski J, Métivier R, Brosseau A, Makal A, Woźniak K (2015). J Org Chem.

[7] Chercka D, Yoo S-J, Baumgarten M, Kim J-J, Müllen K (2014). J Mater Chem C.

[8] Rodenburg L, Brandsma R, Tintel C, van Thuijl J, Lugtenburg J, Cornelisse J (1986). Recl Trav Chim Pays-Bas.

[9] Yamato T, Fujimoto M, Miyazawa A, Matsuo K (1997). J Chem Soc, Perkin Trans 1.

[10] Bock H, Subervie D, Mathey P, Pradhan A, Sarkar P, Dechambenoit P, Hillard E A, Durola F (2014). Org Lett.

[11] Wrona-Piotrowicz A, Zakrzewski J, Métivier R, Brosseau A, Makal A, Woźniak K (2014). RSC Adv.

[12] Wrona-Piotrowicz A, Zakrzewski J, Gajda A, Gajda T, Makal A, Brosseau A, Métivier R (2015). Beilstein J Org Chem.

[13] Ozaki K, Kawasumi K, Shibata M, Ito H, Itami K (2015). Nat Commun.

[14] Duan Z, Hoshino D, Yang Z, Yano H, Ueki H, Liu Y, Ohuchi H, Takayanagi Y, Zhao G, Nishioka Y (2011). Mol Cryst Liq Cryst.

[15] Gonell S, Poyatos M, Peris E (2014). Chem – Eur J.

[16] García R, More S, Melle-Franco M, Mateo-Alonso A (2014). Org Lett.

[17] Paudel A, Hu J-Y, Yamato T (2008). J Chem Res, Synop.

[18] Jang K, Ranasinghe A D, Heske C, Lee D-C (2010). Langmuir.

[19] Mochida K, Kawasumi K, Segawa Y, Itami K (2011). J Am Chem Soc.

[20] Jiang L, Papageorgiou A C, Oh S C, Sağlam Ö, Reichert J, Duncan D A, Zhang Y-Q, Klappenberger F, Guo Y, Allegretti F (2016). ACS Nano.

[21] Jagodziński T S (2003). Chem Rev.

[22] Volkov A, Tinnis F, Slagbrand T, Trillo P, Adolfsson H (2017). Chem Soc Rev.

[23] Zhu R-Y, Farmer M E, Chen Y-Q, Yu J-Q (2016). Angew Chem, Int Ed.

[24] Yamaguchi K, Yajima K, Mizuno N (2012). Chem Commun.

[25] Flamholc R, Plażuk D, Zakrzewski J, Métivier R, Nakatani K, Makal A, Woźniak K (2014). RSC Adv.

