# Supplementary material for: Regioselective (thio)carbamoylation of 2,7-di-tert-butylpyrene at the 1-position with iso(thio)cyanates
Source: Beilstein J Org Chem. 2017 May 29;13:1032–8. doi: 10.3762/bjoc.13.102 (PMC5480332; doi:10.3762/bjoc.13.102)
Supplement: File 1 — Characterization data, copies of 1H, 13C NMR and IR spectra for synthesized compounds, and crystallographic structure and refinement data of 4. [file Beilstein_J_Org_Chem-13-1032-s001.pdf]

Supporting Information

for

Regioselective (thio)carbamoylation of 2,7-di-*tert*-butylpyrene at the 1-position with iso(thio)cyanates

Anna Wrona-Piotrowicz[*,1], Marzena Witalewska[1], Janusz Zakrzewski[*1] and Anna Makal[2]

Address: [1]Department of Organic Chemistry, Faculty of Chemistry, University of Łódź, Tamka 12, 91-403 Łódź, Poland and [2]University of Warsaw, Biological and Chemical Research Center, Żwirki i Wigury 101, 02-089 Warszawa, Poland

Email: Janusz Zakrzewski - janzak@uni.lodz.pl

*Corresponding author

Characterization data, copies of ^{1}H, ^{13}C NMR and IR spectra for synthesized compounds, and crystallographic structure and refinement data of 4.

Content

Characterization data ……………………………………………………………...S2

^{1}H-, ^{13}C NMR and IR spectra of the synthesized compounds……………………………..…S6

Crystallographic and structure refinement data for **4**………………………………………S33

Figure S1. X-ray crystallographic structure of **4**…………………………………………...S36

Characterization data◦

2,7-Di-*tert*-butyl-*N*-butylpyrene-1-carboxamide (3b). White solid (265 mg, 64%); Mp 134 – 136◦C ^{1}H NMR (600 MHz, $CDCl_3$)◦: *δ* 8.25 (s, 1H), 8.20 (m, 2H), 8.03 (m, 3H), 7.96 (d, *J* = 9.0 Hz, 1H), 5.98 (t, *J* = 5.4 Hz, 1H), 3.67 (m, 1H), 3.52 (m, 1H), 1.67 (m, 13H), 1.60 (s, 9H), 1.45 (m, 2H), 0.99 (t, *J* = 7.8 Hz, 3H). ^{13}C NMR (150 MHz, $CDCl_3$): *δ* 171.9, 149.1, 143.9, 131.3, 131.0, 130.9, 130.3, 128.7, 128.3, 128.1, 127.2, 124.4, 123.5, 122.65, 122.63, 122.34, 122.32, 40.0, 37.00, 35.2, 32.3, 31.9, 31.1, 20.3, 13.7. IR (KBr, cm^{-1}): 3430, 2958, 2898, 2870, 1670, 1605, 1393, 1226, 894. Anal. Calcd. for $C_{29}H_{35}NO$: C, 84.22; H, 8.53; N, 3.39; O, 3.87. Found: C, 84.30; H, 8.59; N, 3.27.

2,7-Di-*tert*-butyl-*N*-cyclohexylpyrene-1-carboxamide (3c). White solid (317 mg, 72%). Mp 217 – 218 °C. ^{1}H NMR (600 MHz, $CDCl_3$): *δ* 8.25 (s, 1H), 8.21 (s, 2H), 8.09 (d, *J* = 9.6 Hz, 1H), 8.05 (d, *J* = 9.6 Hz, 1H), 8.00 (d, *J* = 9.0 Hz, 1H), 7.94 (d, *J* = 9.0 Hz, 1H), 5.87 (d, *J* = 8.4 Hz, 1H), 4.24 (m, 1H), 2.27 (d, *J* = 9.6 Hz, 1H), 2.10 (d, *J* = 9.0 Hz, 1H), 1.77 (m, 2H), 1.69 (m, 10H), 1.61 (s, 9H), 1.49 (m, 2H), 1.32 (m, 1H), 1.20 (m, 2H). ^{13}C NMR (150 MHz, $CDCl_3$): *δ* 170.7, 149.1, 143.8, 131.5, 130.9, 130.8, 130.2, 128.6, 128.2, 128.0, 127.2, 124.3, 123.4, 122.6, 122.6, 122.29, 122.28, 48.9, 37.0, 35.2, 33.1, 32.4, 32.3, 31.9, 25.5, 24.81, 24.80. IR (KBr, cm^{-1}): 3433, 3215, 2958, 2930, 1624, 1602, 1553, 1226, 881. Anal. Calcd. for $C_{31}H_{37}NO$: C, 84.69; H, 8.48; N, 3.19; O, 3.64. Found: C, 84.58; H, 8.47; N, 3.25.

***N*,2,7-Tri-*tert*-butylpyrene-1-carboxamide (3d)**. White solid (281 mg, 84%). Mp 148 – 149 (C. ^{1}H NMR (600 MHz, $CDCl_3$): *δ* 8.26 (s, 1H), 8.19 (m, 3H), 8.06 (d, *J* = 9.0 Hz, 1H), 8.03 (d, *J* = 9.0 Hz, 1H), 7.98 (d, *J* = 9.0 Hz, 1H), 5.69 (s, 1H), 1.72 (s, 9H), 1.62 (s, 9H), 1.59 (s, 1H). ^{13}C NMR (150 MHz, $CDCl_3$): *δ* 171.0, 149.1, 143.7, 132.2, 130.94, 130.92, 130.3, 128.9, 128.3, 128.1, 127.2, 124.3, 123.5, 122.8, 122.6, 122.4, 122.3, 52.5, 37.16, 35.21, 32.4, 31.9, 28.7. IR (KBr, cm^{-1}): 3243, 3050, 2961, 1625, 1546, 1363, 1226, 878. Anal. Calcd. for $C_{29}H_{35}NO$: C, 84.22; H, 8.53; N, 3.39; O, 3.87. Found: C, 84.25; H, 8.47; N, 3.43.

2,7-Di-*tert*-butyl-*N*-phenylpyrenecarboxamides (3e) (1:1 mixture of 1- and 4-isomers)

(**3e**). White solid (273 mg, 63%). Mp 242 – 275 °C. ^{1}H NMR (600 MHz, $CDCl_3$): *δ* 8.77 (s, 1H), 8.34 (s, 1H), 8.29 (s, 1H), 8.26 (d, *J* = 1.8 Hz, 1H), 8.24 (m, 2H), 8.23 (d, *J* = 1.2 Hz, 1H), 8.20 (d, *J* = 1.8 Hz, 1H), 8.13 (d, *J* = 9.0 Hz, 1H), 8.07 (d, *J* = 9.0 Hz, 1H), 8.04 (m, 2H), 8.00 (d, *J* = 9.0 Hz, 1H), 7.96 (s, 1H), 7.76 (s, 1H), 7.73 (d, *J* = 7.8 Hz, 2H), 7.64 (s, 1H), 7.44 (m, 4H), 7.22 (m, 2H), 1.70 (s, 9H), 1.60 (s, 9H), 1.59 (s, 9H), 1.57 (s, 9H). ^{13}C NMR (150

MHz, $CDCl_3$): δ 170.1, 149.4, 149.3, 149.2, 144.1, 138.2, 138.0, 131.3, 131.0, 130.9, 130.8, 130.3, 129.3, 129.2, 129.1, 128.8, 128.6, 128.5, 127.9, 127.7, 127.2, 127.18, 127.17, 124.7, 124.6, 124.0, 123.7, 123.6, 123.3, 123.2, 123.1, 123.0, 122.9, 122.7, 122.6, 122.3, 120.6, 120.1, 119.8, 37.1, 35.4, 35.25, 35.24, 32.3, 31.92, 31.91, 31.9. IR (KBr, cm^{-1}): 3427, 3275, 2958, 1652, 1599, 1534, 1440, 1316, 1225, 882. Anal. Calcd. for $C_{31}H_{31}NO$: C, 85.87; H, 7.21; N, 3.23; O, 3.69. Found: C, 85.79; H, 7.30; N, 3.27.

2,7-Di-*tert*-butyl-*N*-isopropylpyrene-1-carbothioamide (3g). Pale yellow solid (370 mg, 89%). Mp 216 – 217 °C. ^{1}H NMR (600 MHz, $CDCl_3$): δ 8.28 (s, 1H), 8.19 (d, J = 1.8 Hz, 1H), 8.17 (d, J = 1.8 Hz, 1H), 8.11 (d, J = 9.0 Hz, 1H), 8.40 (d, J = 9.6 Hz, 1H), 8.01 (d, J = 9.0 Hz, 1H), 7.95 (d, J = 9.0 Hz, 1H), 7.46 (d, J = 7.8 Hz, 1H), 5.08 (m, 1H), 1.75 (s, 9H), 1.59 (s, 9H), 1.50 (d, J = 6.6 Hz, 3H), 1.40 (d, J = 6.6 Hz, 3H). ^{13}C NMR (150 MHz, $CDCl_3$): δ 202.0, 149.2, 142.3, 137.1, 130.9, 130.8, 130.2, 128.4, 128.0, 127.6, 127.2, 124.2, 124.8, 123.0, 122.7, 122.3, 122.3, 47.6, 37.9, 35.2, 32.7, 31.9, 21.2, 20.7; IR (KBr, cm^{-1}): 3432, 3385, 3195, 2963, 1602, 1533, 1456, 1390, 1227, 991, 881. Anal. Calcd. for $C_{28}H_{33}NS$: C, 80.91; H, 8.00; N, 3.37; S, 7.71. Found: C, 80.82; H, 8.07; N, 3.34, S, 7.63.

2,7-Di-*tert*-butyl-*N*-butylpyrene-1-carbothioamide (3h). Pale yellow solid (391 mg, 91%). Mp 179 – 180 °C. ^{1}H NMR (600 MHz, $CDCl_3$): δ 8.28 (s, 1H), 8.19 (d, J = 1.8 Hz, 1H), 8.18 (d, J = 1.8 Hz, 1H), 8.07 (d, J = 9.6 Hz, 1H), 8.03 (d, J = 9.0 Hz, 1H), 8.01 (d, J = 8.4 Hz, 1H), 7.95 (d, J = 9.0 Hz, 1H), 7.61 (s, 1H), 4.07 (m, 1H), 3.82 (m, 1H), 1.77 (m, 2H), 1.72 (s, 9H), 1.59 (s, 9H), 1.48 (m, 2H), 1.00 (t, J = 7.8 Hz, 3H). ^{13}C NMR (150 MHz, $CDCl_3$): δ 203.3, 149.2, 142.5, 137.1, 130.9, 130.8, 130.3, 128.4, 128.0, 127.7, 127.2, 124.9, 124.3, 124.2, 122.9, 122.7, 122.29, 122.28, 46.0, 37.7, 35.2, 32.6, 31.9, 29.6, 20.4, 13.7. IR (KBr, cm^{-1}): 3167, 2958, 1600, 1540, 1360, 1224, 883. Anal. Calcd. for $C_{29}H_{35}NS$: C, 81.07; H, 8.21; N, 3.26; S, 7.46. Found: C, 80.99; H, 8.28; N, 3.24, S, 7.40.

***N*,2,7-Tri-*tert*-butylpyrene-1-carbothioamide (3i)**. Pale yellow solid (400 mg, 93%). Mp 121 – 123 °C. ^{1}H NMR (600 MHz, $CDCl_3$): δ 8.30 (s, 1H), 8.28 (d, J = 9.0 Hz, 1H), 8.20 (d, J = 1.8 Hz, 1H), 8.19 (d, J = 1.8 Hz, 1H), 8.08 (d, J = 9.0 Hz, 1H), 8.01 (d, J = 9.0 Hz, 1H), 7.96 (d, J = 9.0 Hz, 1H), 7.46 (s, 1H), 1.81 (s, 9H), 1.80 (s, 9H), 1.61 (s, 9H). ^{13}C NMR (150 MHz, $CDCl_3$): δ 202.6, 149.1, 141.7, 138.5, 130.9, 130.6, 130.2, 128.3, 127.9, 127.6, 127.1, 124.4, 124.3, 123.1, 122.6, 122.3, 122.2, 57.0, 37.9, 35.2, 32.8, 31.9, 27.4. IR (KBr, cm^{-1}): 3379, 2955, 1601, 1500, 1456, 1358, 1260, 1225, 880. Anal. cCalcd. for $C_{29}H_{35}NS$: C, 81.07; H, 8.21; N, 3.26; S, 7.46. Found: C, 81.11; H, 8.22; N, 3.19, S, 7.35.

2,7-Di-*tert*-butyl-*N*-hexylpyrene-1-carbothioamide (3j). Pale yellow solid (398 mg, 87%). Mp 168 – 169 °C. ^{1}H NMR (600 MHz, $CDCl_3$): δ 8.28 (s, 1H), 8.19 (d, J = 1.8 Hz, 1H), 8.17 (d, J = 1.8 Hz, 1H), 8.07 (d, J = 9.6 Hz, 1H), 8.02 (d, J = 9.0 Hz, 1H), 8.01 (d, J = 8.4 Hz, 1H), 7.95 (d, J = 9.0 Hz, 1H), 7.63 (s, 1H), 4.07 (m, 1H), 3.83 (m, 1H), 1.79 (m, 2H), 1.72 (s, 9H), 1.59 (s, 9H), 1.45 (m, 2H), 1.35 (m, 4H), 0.92 (t, J = 6.6 Hz, 3H). ^{13}C NMR (150 MHz, $CDCl_3$): δ 203.3, 149.2, 142.4, 137.1, 130.80, 130.27, 128.3, 128.0, 127.7, 127.2, 124.4, 124.2, 122.9, 122.7, 122.29, 122.27, 46.3, 37.7, 35.2, 32.6, 31.9, 31.4, 27.5, 26.9, 22.5, 14.0. IR (KBr, cm^{-1}): 3221, 2955, 1600, 1533, 1360, 1227, 880. Anal. Calcd. for $C_{31}H_{39}NS$: C, 81.35; H, 8.59; N, 3.06; S, 7.01. Found: C, 81.37; H, 8.53; N, 3.08, S, 7.02.

2,7-Di-*tert*-butyl-*N*-cyclohexylpyrene-1-carbothioamide (3k). Pale yellow solid (415 mg, 91%) Mp 277 – 278 °C. ^{1}H NMR (600 MHz, $CDCl_3$): δ 8.28 (s, 1H), 8.18 (d, J = 1.2 Hz, 1H), 8.16 (d, J = 1.8 Hz, 1H), 8.07 (d, J = 9.6 Hz, 1H), 8.02 (d, J = 9.6 Hz, 1H), 8.00 (d, J = 9.0 Hz, 1H), 7.95 (d, J = 8.4 Hz, 1H), 7.49 (d, J = 7.2 Hz, 1H), 4.84 (m, 1H), 2.44 (m, 1H), 2.24 (m, 1H), 1.81 (m, 2H), 1.74 (s, 9H), 1.71 (m, 1H), 1.58 (s, 9H), 1.55 (m, 2H), 1.41 (m, 1H), 1.35 (m, 1H), 1.27 (m, 1H). ^{13}C NMR (150 MHz, $CDCl_3$): δ 201.8, 149.2, 142.3, 137.2, 131.0, 130.8, 130.3, 128.4, 128.0, 127.6, 127.2, 124.3, 124.2, 123.0, 122.7, 122.31, 122.27, 54.3, 37.9, 35.2, 32.75, 32.4, 31.9, 31.4, 31.0, 25.5, 24.6. IR (KBr, cm^{-1}): 3433, 3386, 3155, 2965, 2927, 1603, 1540, 1385, 1359, 1223, 985, 878. Anal. Calcd. for $C_{31}H_{37}NS$: C, 81.71; H, 8.18; N, 3.07; S, 7.04. Found: C, 81.77; H, 8.15; N, 3.09, S, 6.95.

2,7-Di-*tert*-butyl-*N*-benzylpyrene-1-carbothioamide (3l). Yellow solid (415 mg, 87%). Mp 115 – 116 °C. ^{1}H NMR (600 MHz, $CDCl_3$): δ 8.28 (s, 1H), 8.20 (d, J = 1.8 Hz, 1H), 8.19 (d, J = 1.8 Hz, 1H), 8.12 (d, J = 9.0 Hz, 1H), 8.04 (d, J = 9.6 Hz, 1H), 8.01 (d, J = 9.0 Hz, 1H), 7.95 (d, J = 9.0 Hz, 1H), 7.79 (t, J = 4.2 Hz, 1H), 7.42 (m, 2H), 7.36 (m, 2H), 7.32 (m, 1H), 5.25 (dd, J_1 = 14.4 Hz, J_2 = 4.8 Hz, 1H), 4.90 (dd, J_1 = 14.4 Hz, J_2 = 4.8 Hz, 1H), 1.74 (s, 9H), 1.60 (s, 9H). ^{13}C NMR (150 MHz, $CDCl_3$): δ 203.1, 149.2, 142.5, 136.7, 135.5, 130.93, 130.86, 130.2, 129.0, 128.8, 128.4, 128.3, 128.05, 127.7, 127.2, 124.3, 124.1, 122.9, 122.7, 122.3, 122.2, 50.9, 37.7, 35.2, 32.6, 31.9. IR (KBr, cm^{-1}): 3395, 3351, 2958, 1602, 1497, 1380, 1360, 1226, 881. Anal. Calcd. for $C_{32}H_{33}NS$: C, 82.89; H, 7.17; N, 3.02; S, 6.92. Found: C, 82.95; H, 7.21; N, 2.94, S, 6.86.

2,7-Di-*tert*-butyl-*N*-phenylpyrenecarbothioamides (3m). (Mixture of 1 and 4- isomers_ (3:1). Pale brown solid (369 mg, 82%). Mp 238 – 247 °C. Main component: ^{1}H NMR (600 MHz, $CDCl_3$): δ 8.34 (s, 1H), 8.27 (d, J = 9.6 Hz, 1H), 8.21 (d, J = 1.8 Hz, 1H), 8.19 (d, J =

1.8 Hz, 1H), 8.06 (d, *J* = 9.6 Hz, 1H), 8.04 (d, *J* = 9.0 Hz, 1H), 7.99 (d, *J* = 9.0 Hz, 1H), 7.96 (m, 2H), 7.49 (m, 2H), 7.35 (m, 1H), 6.88 (m, 1H), 1.79 (s, 9H), 1.60 (s, 9H); ^{13}C NMR (150 MHz, $CDCl_3$): δ 202.1, 149.3, 142.3, 138.5, 137.9, 131.0, 130.3, 129.2, 129.2, 128.7, 128.2, 127.5, 127.2, 127.1, 124.4, 124.1, 123.0, 122.9, 122.8, 122.4, 122.3, 119.8, 37.9, 32.8, 32.5, 31.9. IR (KBr, cm^{-1}): 3436, 3378, 3212, 3050, 2961, 1597, 1549, 1363, 1226, 880. Anal. Calcd. for $C_{31}H_{31}NS$: C, 82.80; H, 6.95; N, 3.12; S, 7.13. Found: C, 82.85; H, 7.01; N, 3.03, S, 7.05.

2,7-Di-*tert*-butyl-*N*-(4-methoxyphenyl)pyrene-1-carbothioamide (3n). Yellow solid (427 mg, 89%). Mp 237 – 238 °C. ^{1}H NMR (600 MHz, DMSO): δ 12.36 (s, 1H), 8.47 (s, 1H), 8.36 (d, *J* = 1.2 Hz, 1H), 8.29 (d, *J* = 1.8 Hz, 1H), 8.18 (d, *J* = 9.6 Hz, 1H), 8.14 (m, 3H), 8.04 (m, 2H), 7.08 (m, 2H), 3.82 (s, 1H), 1.73(s, 9H), 1.55 (s, 9H). ^{13}C NMR (150 MHz, DMSO): δ 199.2, 158.0, 149.4, 142.2, 138.5, 133.1, 131.1, 130.4, 130.3, 128.5, 128.1, 127.7, 127.2, 125.01, 124.97, 124.8, 123.1, 122.6, 122.45, 122.1, 114.4, 55.9, 38.1, 35.5, 32.9, 32.2. IR (KBr, cm^{-1}): 3173, 2949, 1602, 1546, 1507, 1378, 1247, 1226, 1037, 835. Anal. Calcd. for $C_{32}H_{33}NOS$: C, 80.13; H, 6.93; N, 2.92; O, 3.34; S, 6.68. Found: C, 80.17; H, 7.02; N, 2.84, S, 6.65.

3a

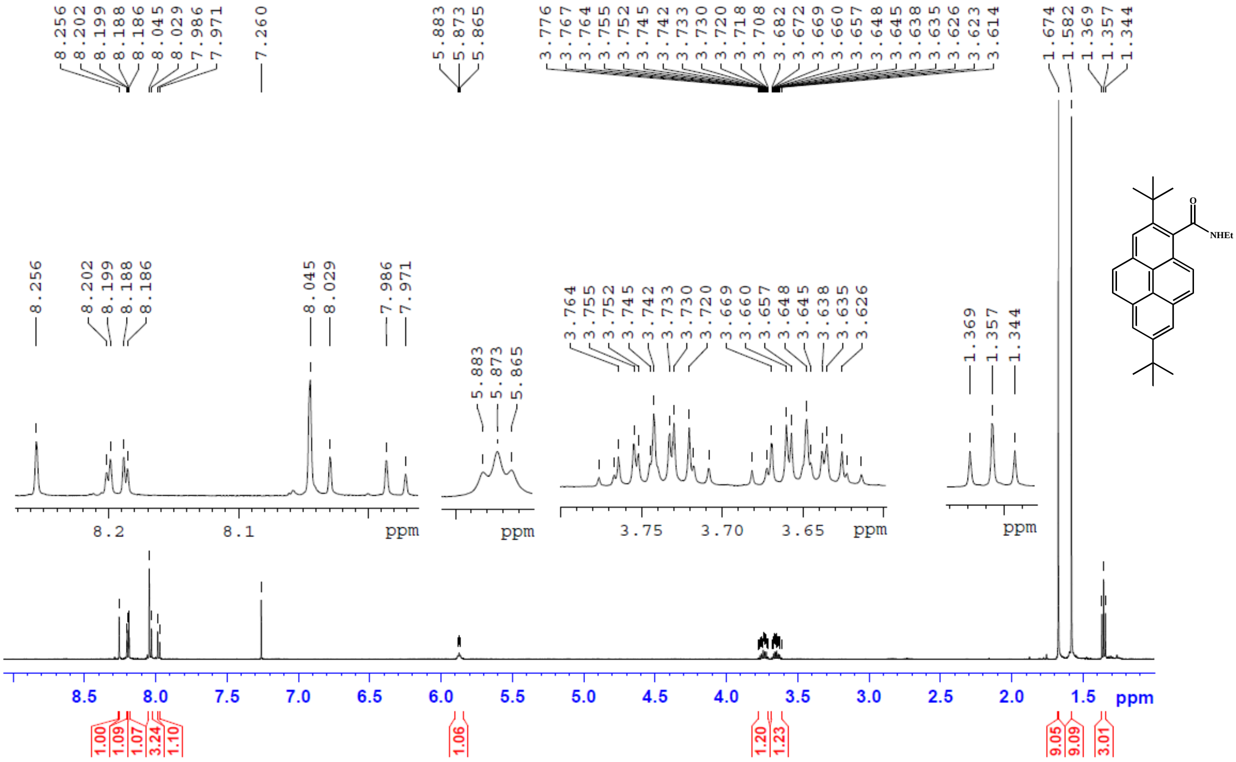
8.256 8.202 8.199 8.188 8.186 8.045 8.029 7.986 7.971
7.260
5.883 5.873 5.865
3.776 3.767 3.764 3.755 3.752 3.745 3.742 3.733 3.730 3.720 3.718 3.708 3.682 3.672 3.669 3.660 3.657 3.648 3.645 3.638 3.635 3.626 3.623 3.614
1.674 1.582 1.369 1.357 1.344
NHEt
ppm
1.00 1.09 1.07 3.24 1.10 1.06 1.20 1.23 9.05 9.09 3.01

3a

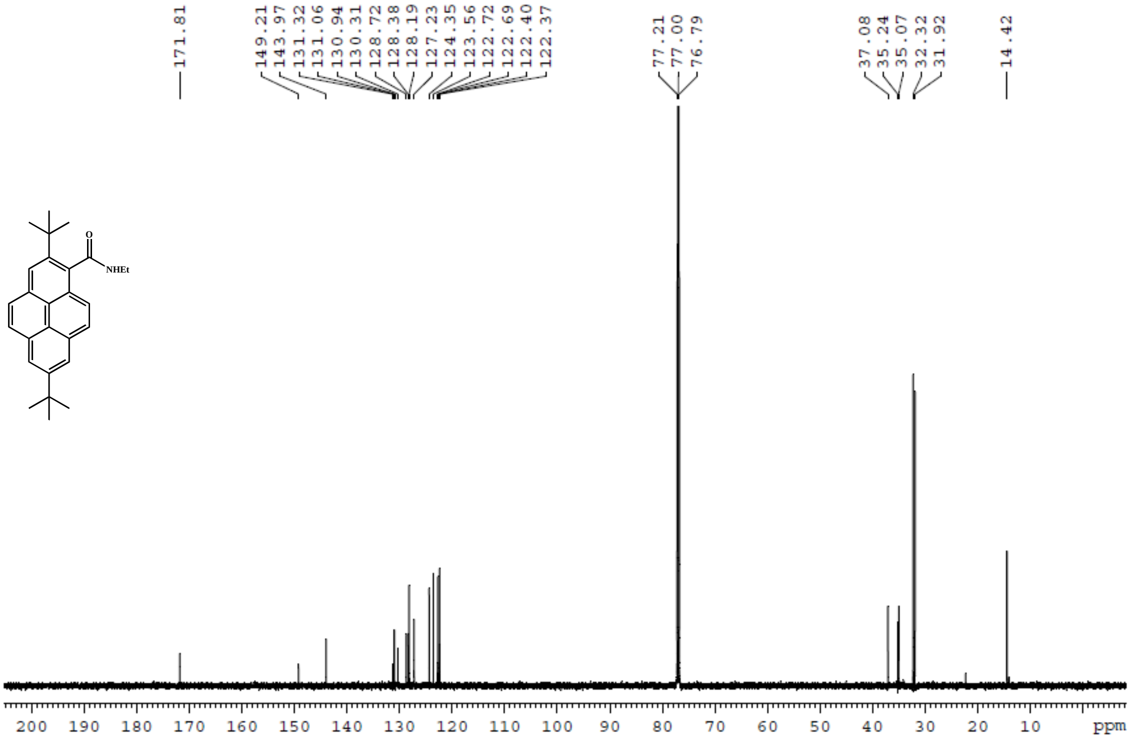
171.81
149.21 143.97 131.32 131.06 130.94 130.31 128.72 128.38 128.19 127.23 124.35 123.56 122.72 122.69 122.40 122.37
77.21 77.00 76.79
37.08 35.24 35.07 32.32 31.92
14.42
NHEt
ppm

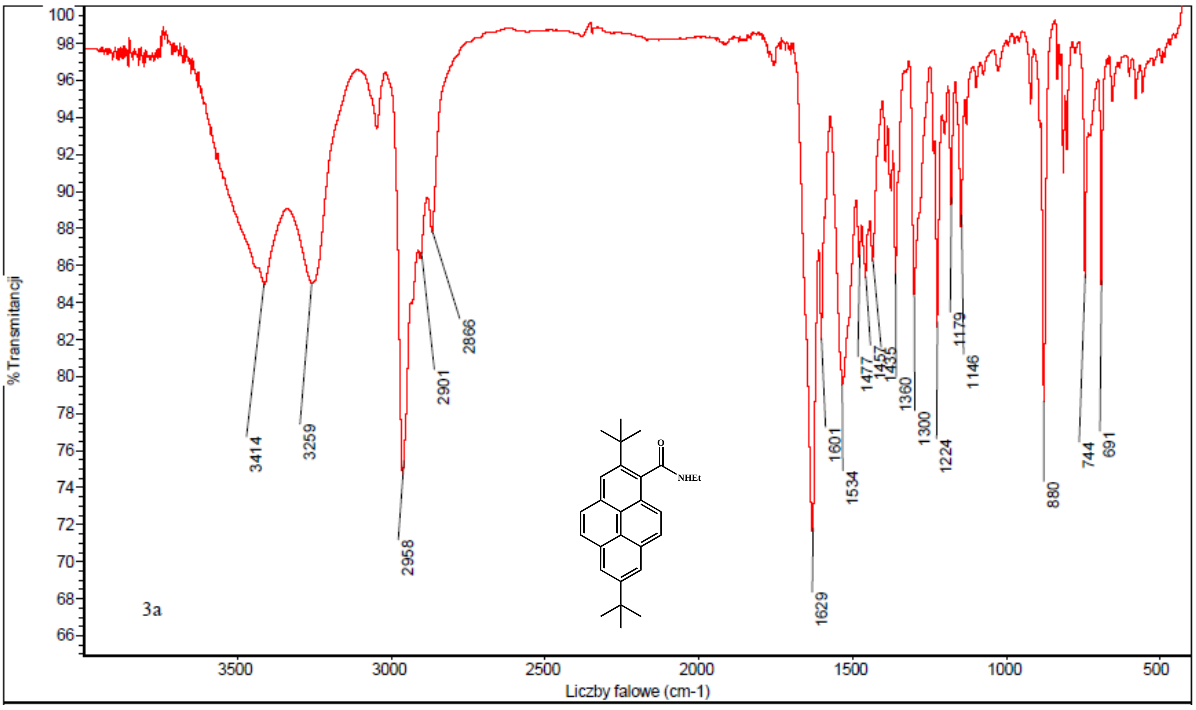

% Transmitancji
Liczby falowe (cm-1)
3a
NHEt
3414
3259
2958
2901
2866
1629
1601
1534
1477
1457
1435
1360
1300
1224
1179
1146
880
744
691

3b

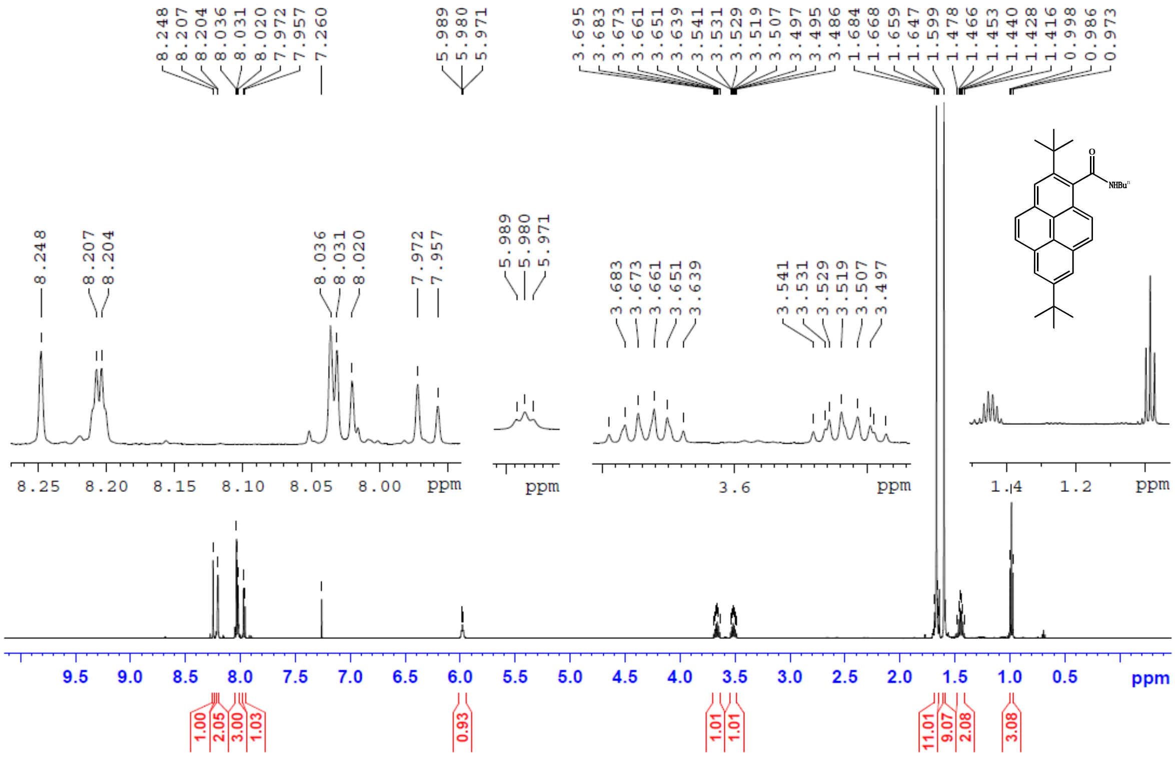

8.248
8.207
8.204
8.036
8.031
8.020
7.972
7.957
7.260
5.989
5.980
5.971
3.695
3.683
3.673
3.661
3.651
3.639
3.541
3.531
3.529
3.519
3.507
3.497
3.495
3.486
1.684
1.668
1.659
1.647
1.599
1.478
1.466
1.453
1.440
1.428
1.416
0.998
0.986
0.973
NHBu^n
ppm
1.00
2.05
3.00
1.03
0.93
1.01
1.01
11.01
9.07
2.08
3.08

3b

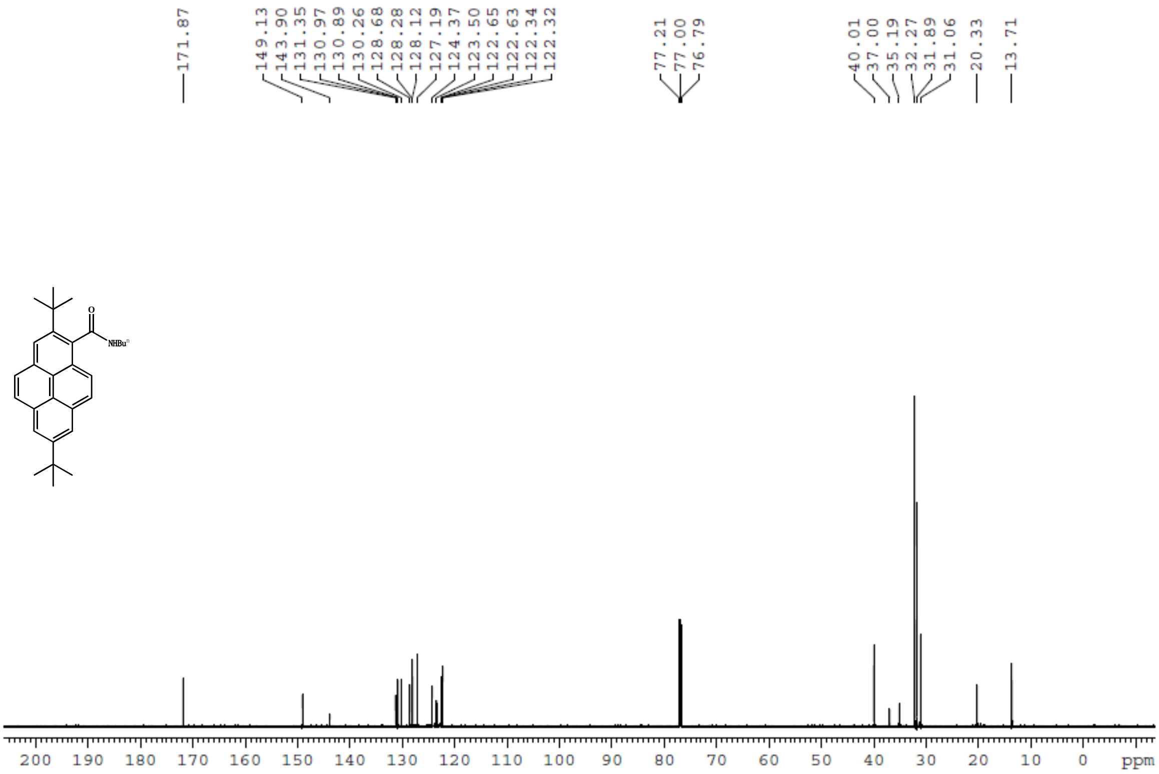
171.87
149.13
143.90
131.35
130.97
130.89
130.26
128.68
128.28
128.12
127.19
124.37
123.50
122.65
122.63
122.34
122.32
77.21
77.00
76.79
40.01
37.00
35.19
32.27
31.89
31.06
20.33
13.71
O
NHBu^n
200 190 180 170 160 150 140 130 120 110 100 90 80 70 60 50 40 30 20 10 0 ppm

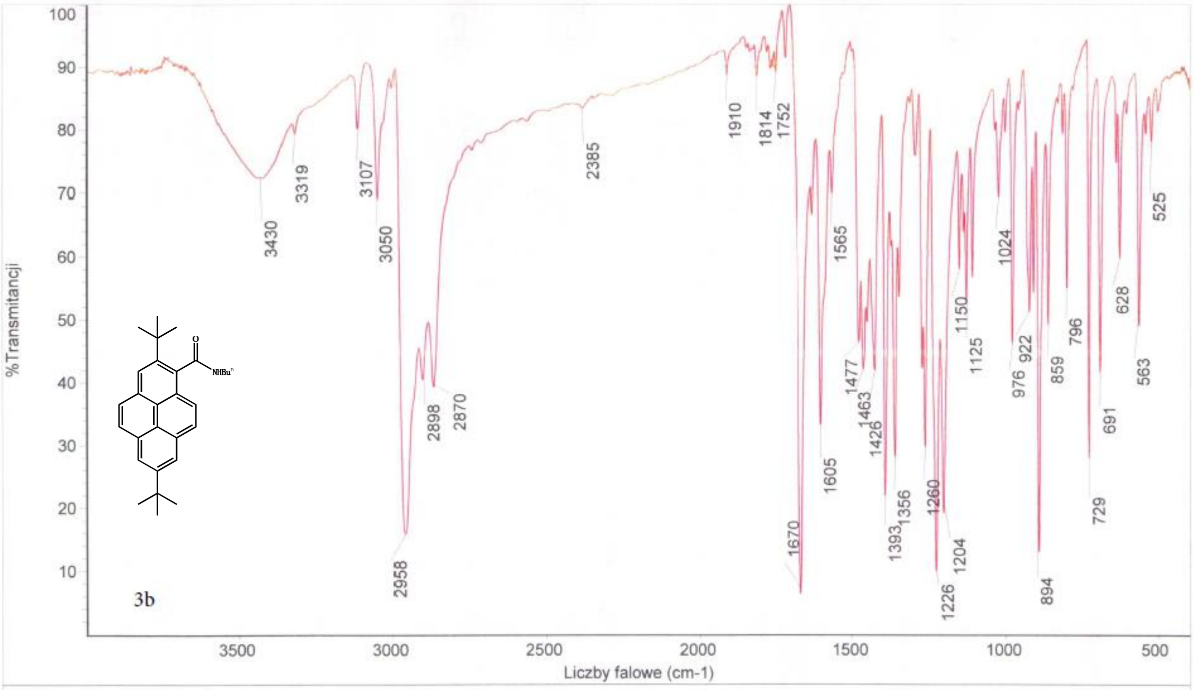
%Transmitancji
Liczby falowe (cm-1)
3430
3319
3107
3050
2958
2898
2870
2385
1910
1814
1752
1670
1605
1565
1477
1463
1426
1393
1356
1260
1226
1204
1150
1125
1024
976
922
894
859
796
729
691
628
563
525
O
NHBu^n
3b

3c

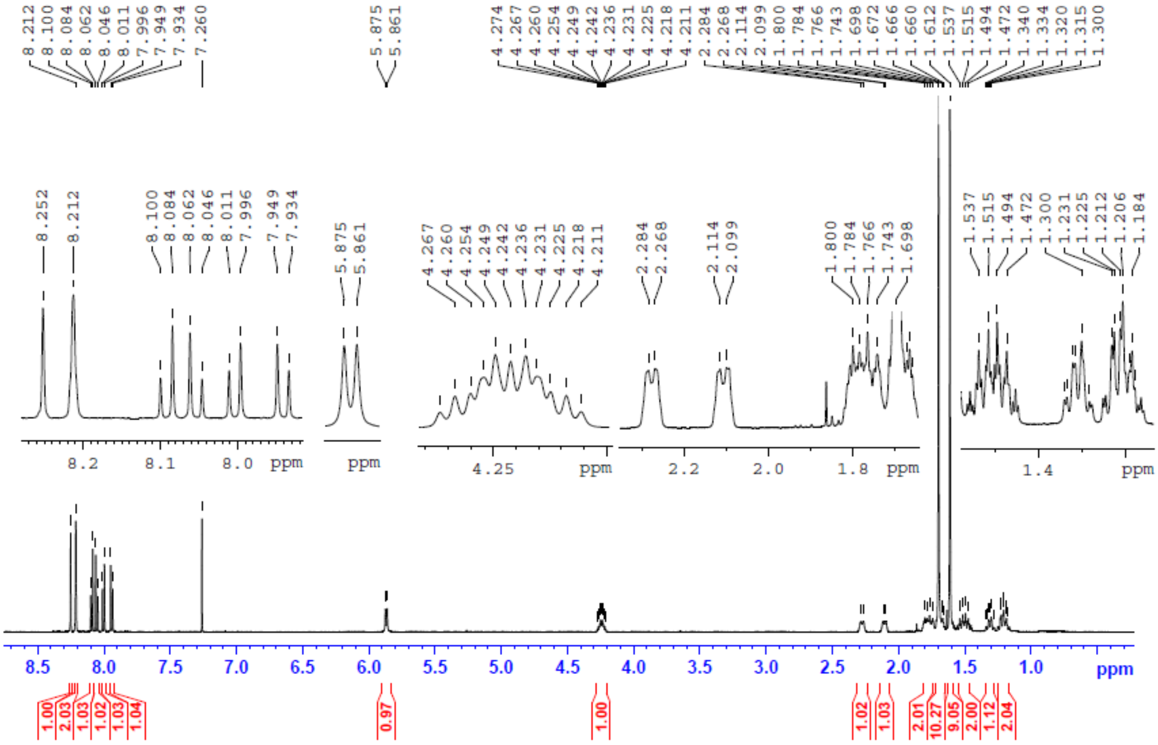
8.212 8.100 8.084 8.062 8.046 8.011 7.996 7.949 7.934 7.260
5.875 5.861
4.274 4.267 4.260 4.254 4.249 4.242 4.236 4.231 4.225 4.218 4.211
2.284 2.268 2.114 2.099 1.800 1.784 1.766 1.743 1.698 1.672 1.666 1.660 1.612 1.537 1.515 1.494 1.472 1.340 1.334 1.320 1.315 1.300
8.252 8.212 8.100 8.084 8.062 8.046 8.011 7.996 7.949 7.934
1.537 1.515 1.494 1.472 1.300 1.231 1.225 1.212 1.206 1.184
ppm
1.00 2.03 1.03 1.02 1.03 1.04 0.97 1.00 1.02 1.03 2.01 10.27 9.05 2.00 1.12 2.04

3c

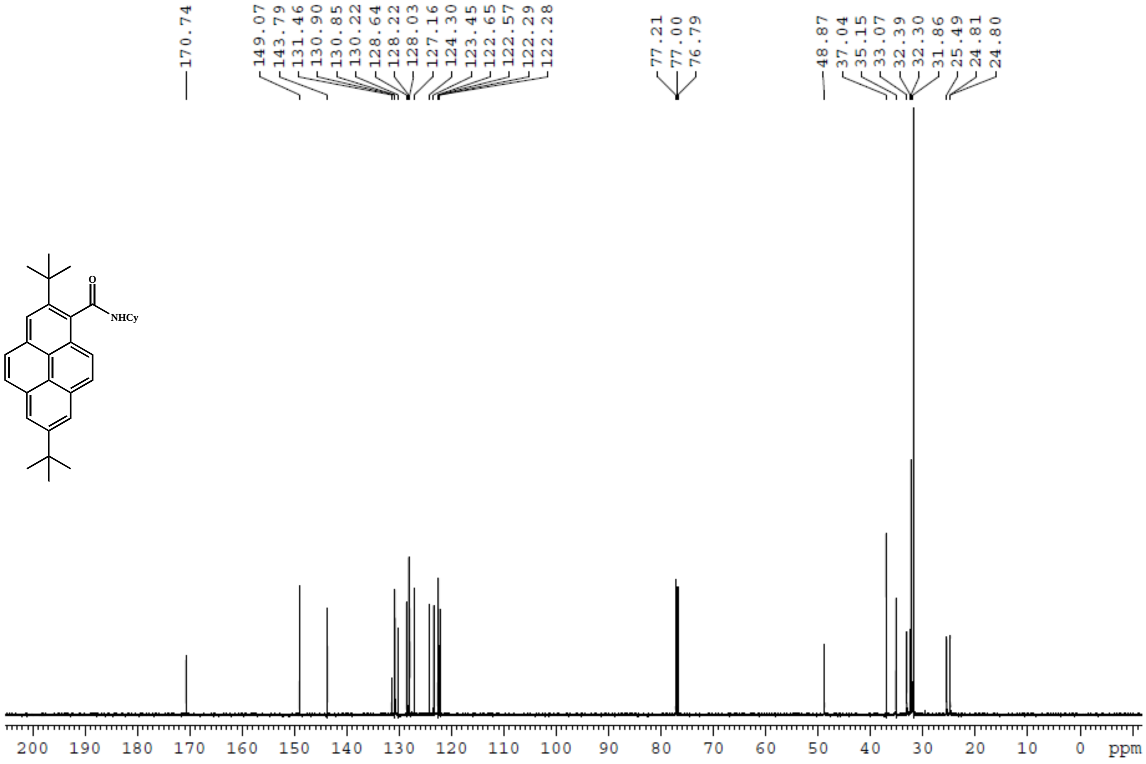
170.74
149.07 143.79 131.46 130.90 130.85 130.22 128.64 128.22 128.03 127.16 124.30 123.45 122.65 122.57 122.29 122.28
77.21 77.00 76.79
48.87 37.04 35.15 33.07 32.39 32.30 31.86 25.49 24.81 24.80
O
NHCy
ppm

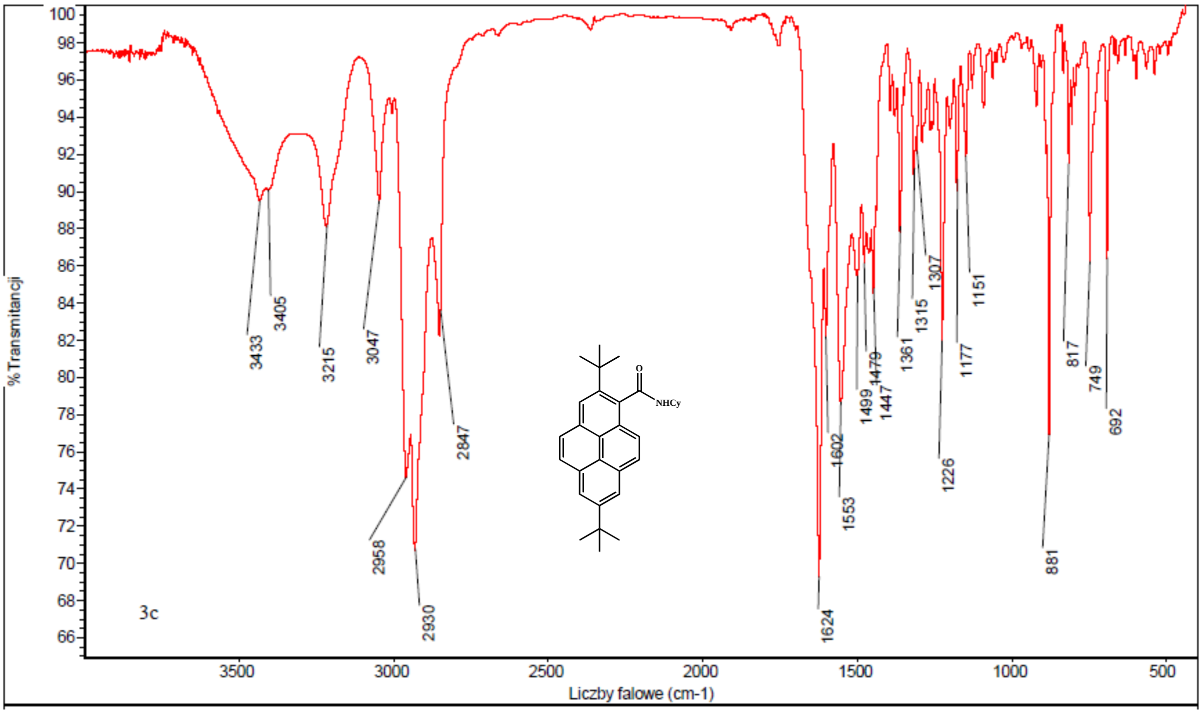

% Transmitancji
Liczby falowe (cm-1)
3c
NHCy
O
3433
3405
3215
3047
2958
2930
2847
1624
1602
1553
1499
1479
1447
1361
1315
1307
1226
1177
1151
881
817
749
692

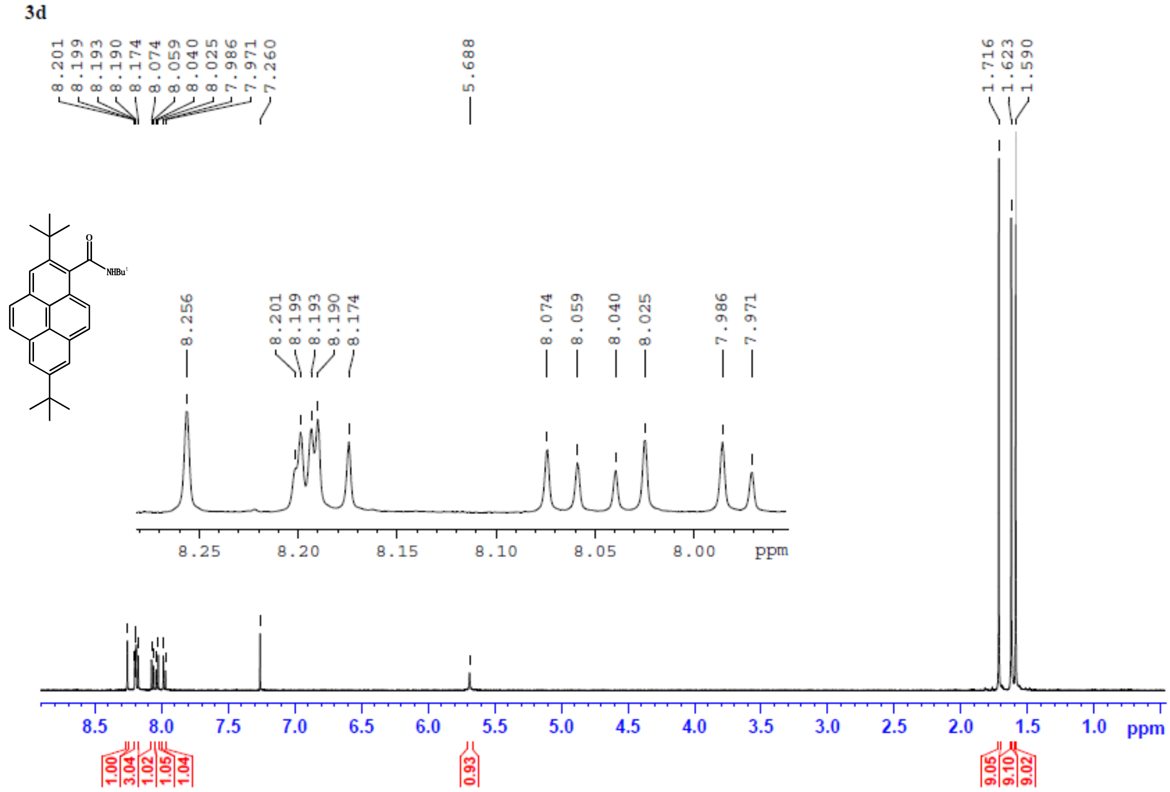

3d
NHBu
O
8.256
8.201
8.199
8.193
8.190
8.174
8.074
8.059
8.040
8.025
7.986
7.971
7.260
5.688
1.716
1.623
1.590
ppm
1.00
3.04
1.02
1.05
1.04
0.93
9.05
9.10
9.02

3d

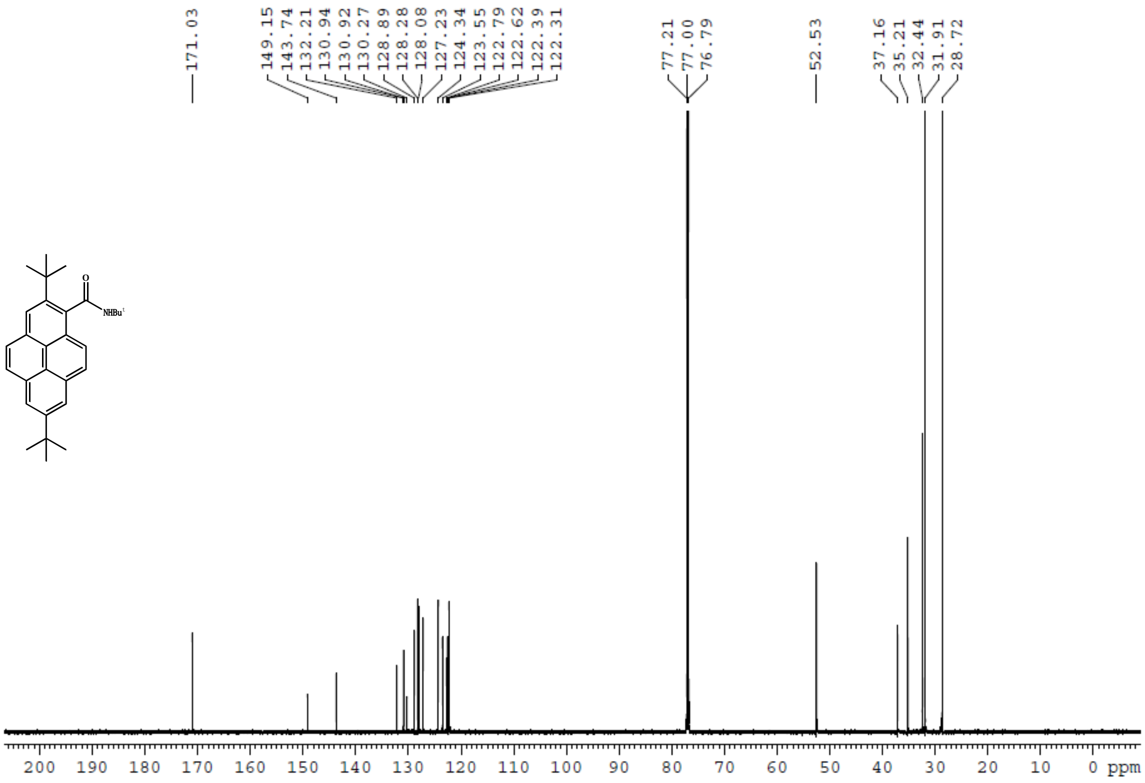
171.03
149.15
143.74
132.21
130.94
130.92
130.27
128.89
128.28
128.08
127.23
124.34
123.55
122.79
122.62
122.39
122.31
77.21
77.00
76.79
52.53
37.16
35.21
32.44
31.91
28.72
O
NHBu[t]
200 190 180 170 160 150 140 130 120 110 100 90 80 70 60 50 40 30 20 10 0 ppm

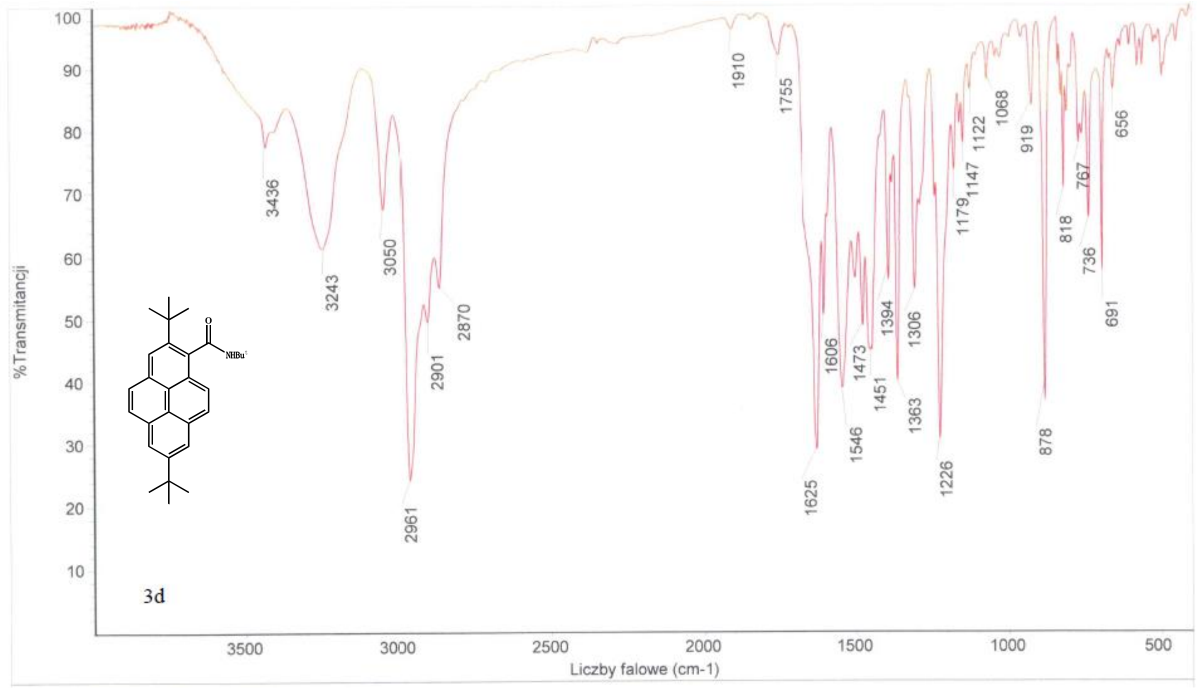
%Transmitancji
Liczby falowe (cm-1)
3d
O
NHBu[t]
3436
3243
3050
2961
2901
2870
1910
1755
1625
1606
1546
1473
1451
1394
1363
1306
1226
1179
1147
1122
1068
919
878
818
767
736
691
656

3e

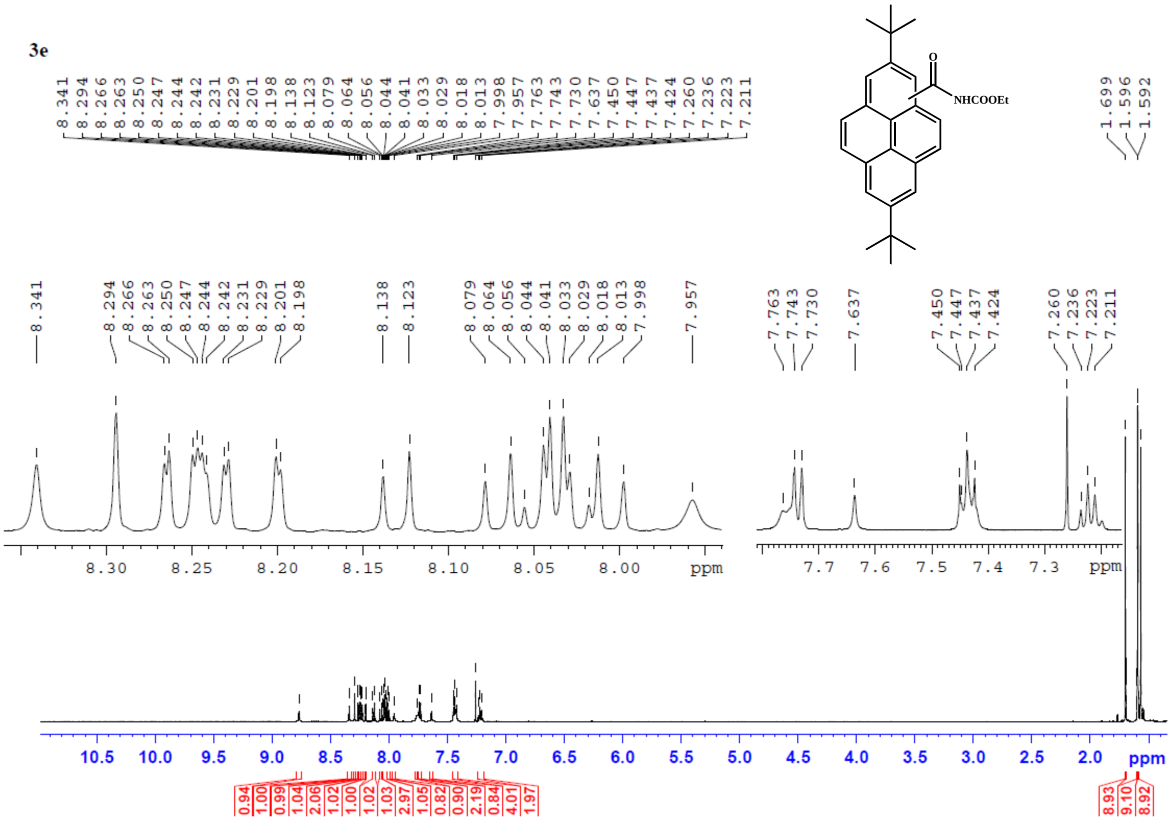

8.341 8.294 8.266 8.263 8.250 8.247 8.244 8.242 8.231 8.229 8.201 8.198 8.138 8.123 8.079 8.064 8.056 8.044 8.041 8.033 8.029 8.018 8.013 7.998 7.957 7.763 7.743 7.730 7.637 7.450 7.447 7.437 7.424 7.260 7.236 7.223 7.211
1.699 1.596 1.592
NHCOOEt
O
8.30 8.25 8.20 8.15 8.10 8.05 8.00 ppm
7.7 7.6 7.5 7.4 7.3 ppm
10.5 10.0 9.5 9.0 8.5 8.0 7.5 7.0 6.5 6.0 5.5 5.0 4.5 4.0 3.5 3.0 2.5 2.0 ppm
0.94 1.00 0.99 1.04 2.06 1.02 1.00 1.02 1.03 2.97 1.05 0.82 0.90 2.19 0.84 4.01 1.97
8.93 9.10 8.92

3e

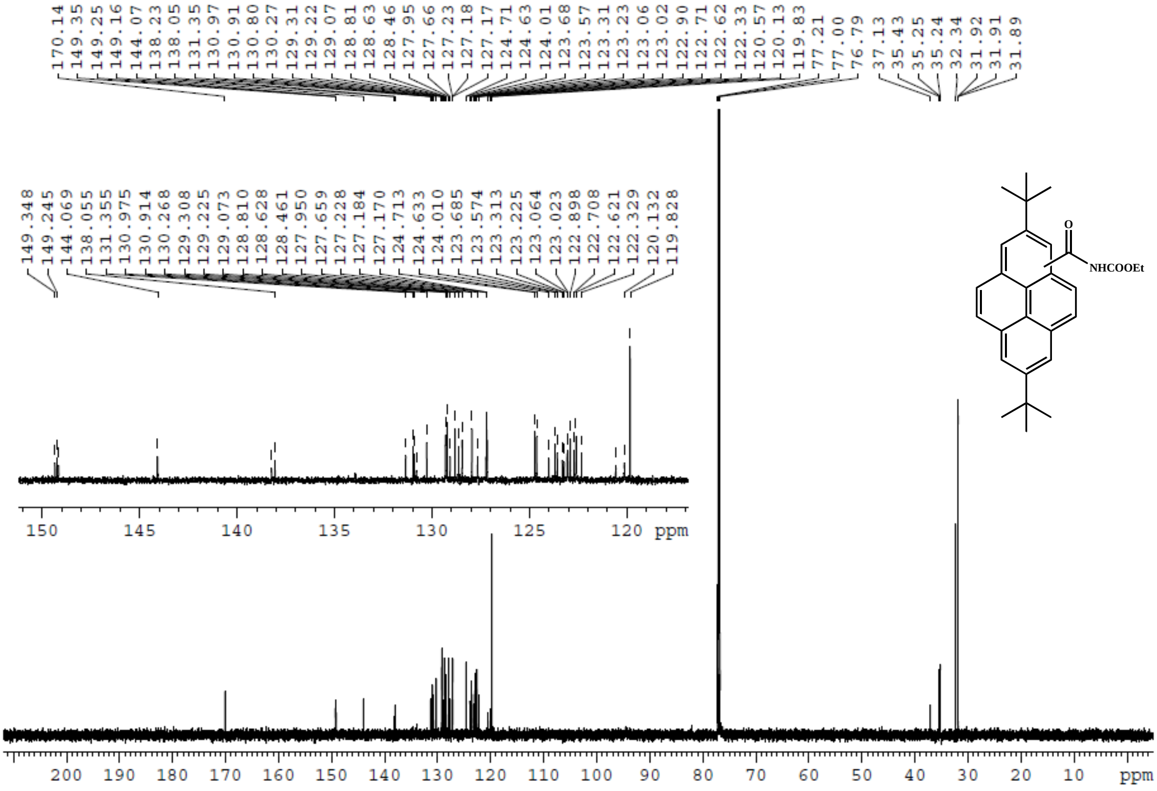

170.14 149.35 149.25 149.16 144.07 138.23 138.05 131.35 130.97 130.91 130.80 130.27 129.31 129.22 129.07 128.81 128.63 128.46 127.95 127.66 127.23 127.18 127.17 124.71 124.63 124.01 123.68 123.57 123.31 123.23 123.06 123.02 122.90 122.71 122.62 122.33 120.57 120.13 119.83 77.21 77.00 76.79 37.13 35.43 35.25 35.24 32.34 31.92 31.91 31.89
149.348 149.245 144.069 138.055 131.355 130.975 130.914 130.268 129.308 129.225 129.073 128.810 128.628 128.461 127.950 127.659 127.228 127.184 127.170 124.713 124.633 124.010 123.685 123.574 123.313 123.225 123.064 123.023 122.898 122.708 122.621 122.329 120.132 119.828
150 145 140 135 130 125 120 ppm
NHCOOEt
O
200 190 180 170 160 150 140 130 120 110 100 90 80 70 60 50 40 30 20 10 ppm

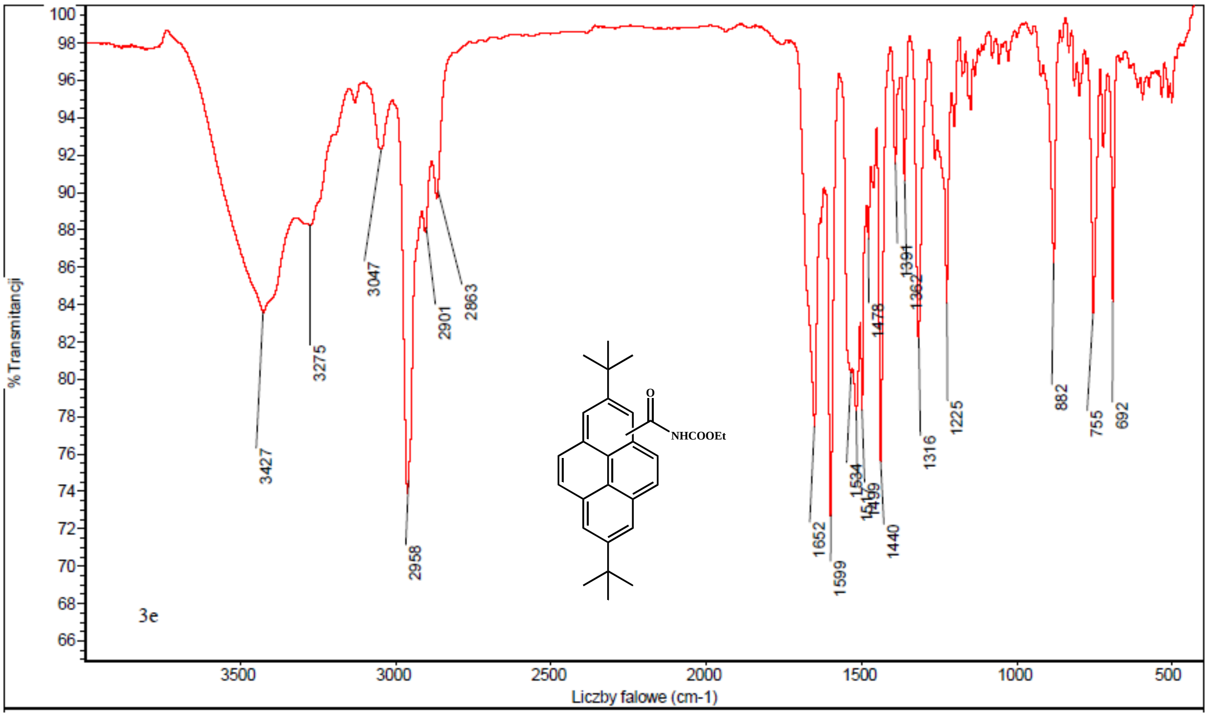
% Transmitancji
Liczby falowe (cm-1)
3e
NHCOOEt
3427
3275
3047
2958
2901
2863
1652
1599
1534
1440
1478
1391
1362
1316
1225
882
755
692

3f

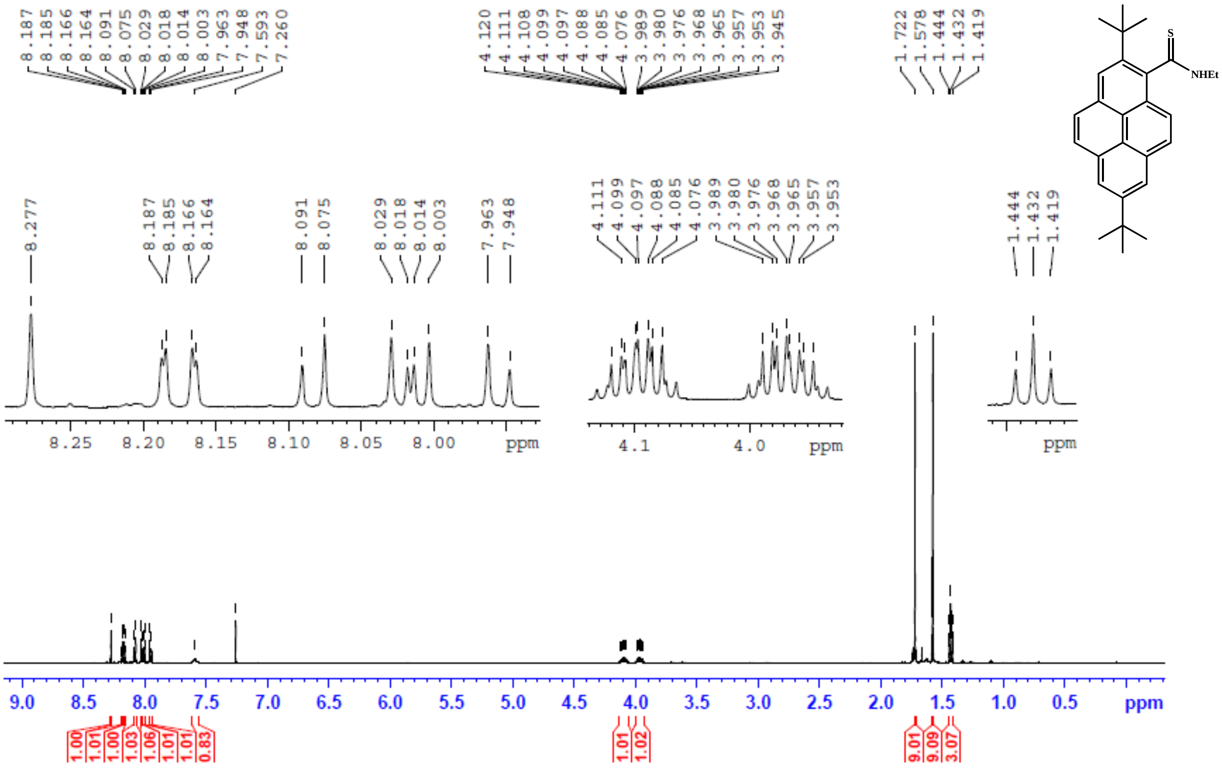
NHEt
ppm
9.01
9.09
3.07

3f

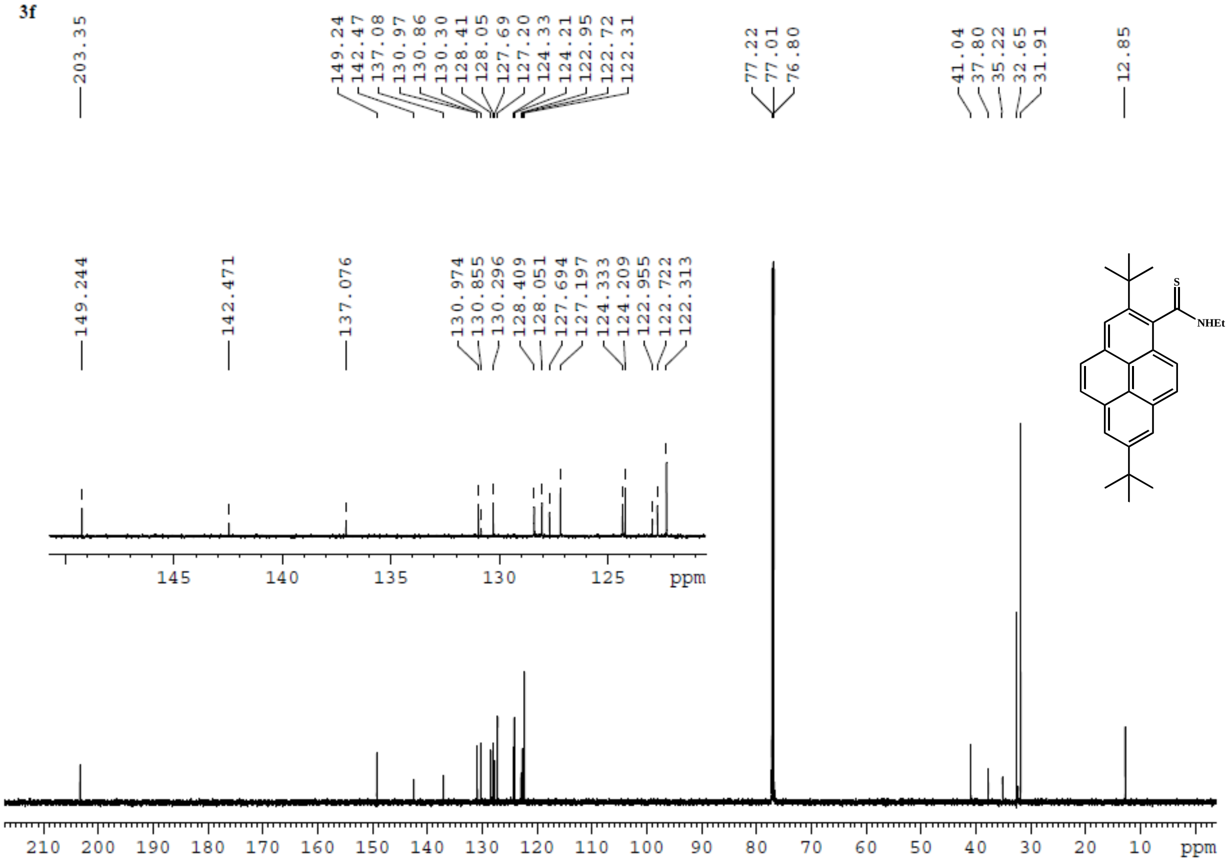
203.35
149.24
142.47
137.08
130.97
130.86
130.30
128.41
128.05
127.69
127.20
124.33
124.21
122.95
122.72
122.31
77.22
77.01
76.80
41.04
37.80
35.22
32.65
31.91
12.85
149.244
142.471
137.076
130.974
130.855
130.296
128.409
128.051
127.694
127.197
124.333
124.209
122.955
122.722
122.313
ppm
S
NHEt
ppm

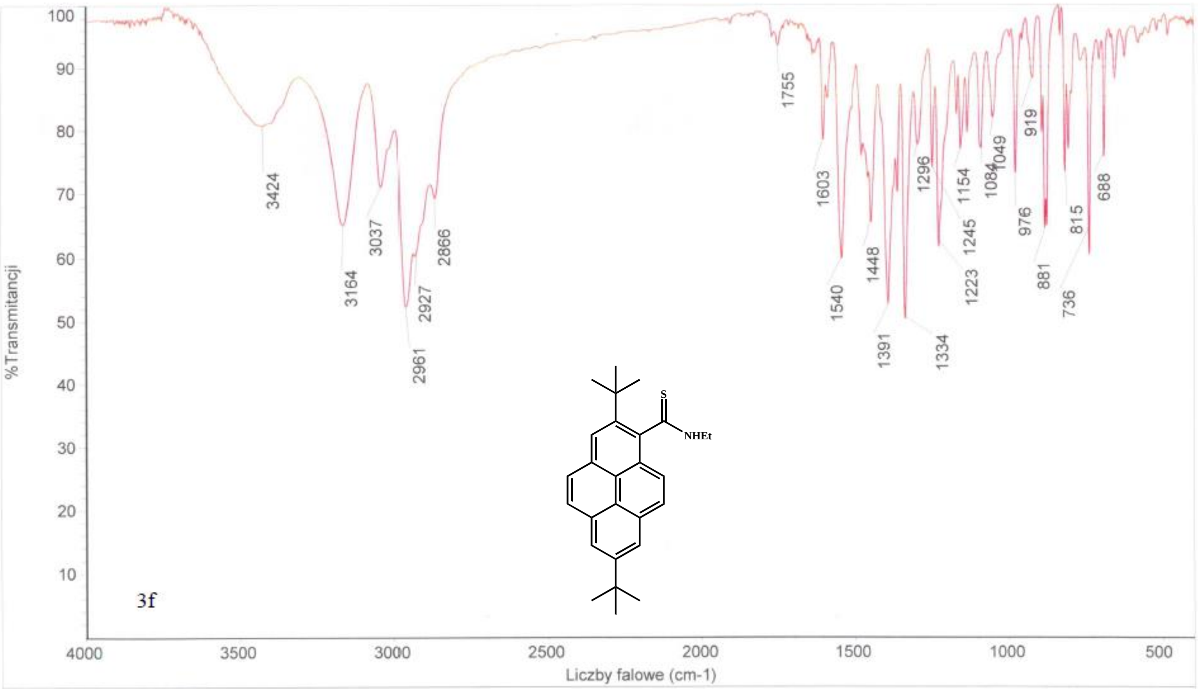
%Transmitancji
Liczby falowe (cm-1)
3424
3164
3037
2961
2927
2866
1755
1603
1540
1448
1391
1334
1296
1245
1223
1154
1084
1049
976
919
881
815
736
688
S
NHEt
3f

3g

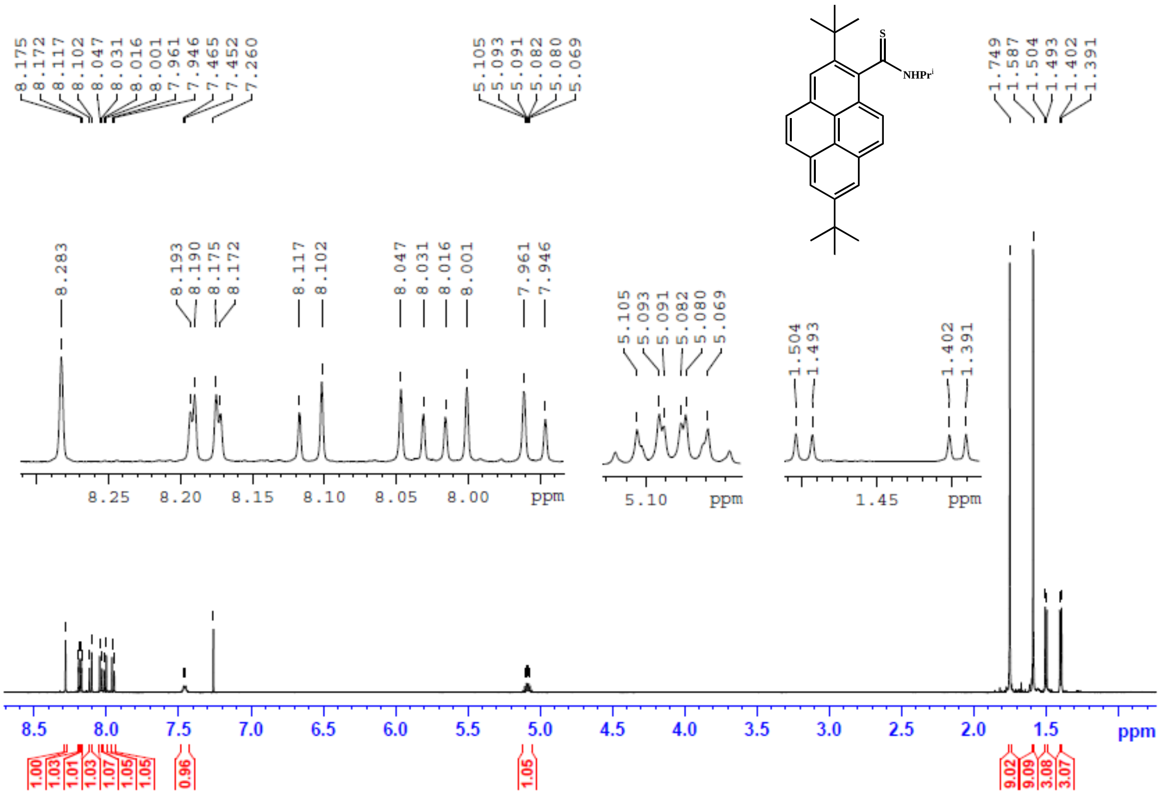

8.175 8.172 8.117 8.102 8.047 8.031 8.016 8.001 7.961 7.946 7.465 7.452 7.260
5.105 5.093 5.091 5.082 5.080 5.069
S
NHPr^i
1.749 1.587 1.504 1.493 1.402 1.391
8.283 8.193 8.190 8.175 8.172 8.117 8.102 8.047 8.031 8.016 8.001 7.961 7.946
8.25 8.20 8.15 8.10 8.05 8.00 ppm
5.105 5.093 5.091 5.082 5.080 5.069
5.10 ppm
1.504 1.493 1.402 1.391
1.45 ppm
8.5 8.0 7.5 7.0 6.5 6.0 5.5 5.0 4.5 4.0 3.5 3.0 2.5 2.0 1.5 ppm
1.00 1.03 1.01 1.03 1.07 1.05 1.05 0.96 1.05 9.02 9.09 3.08 3.07

3g

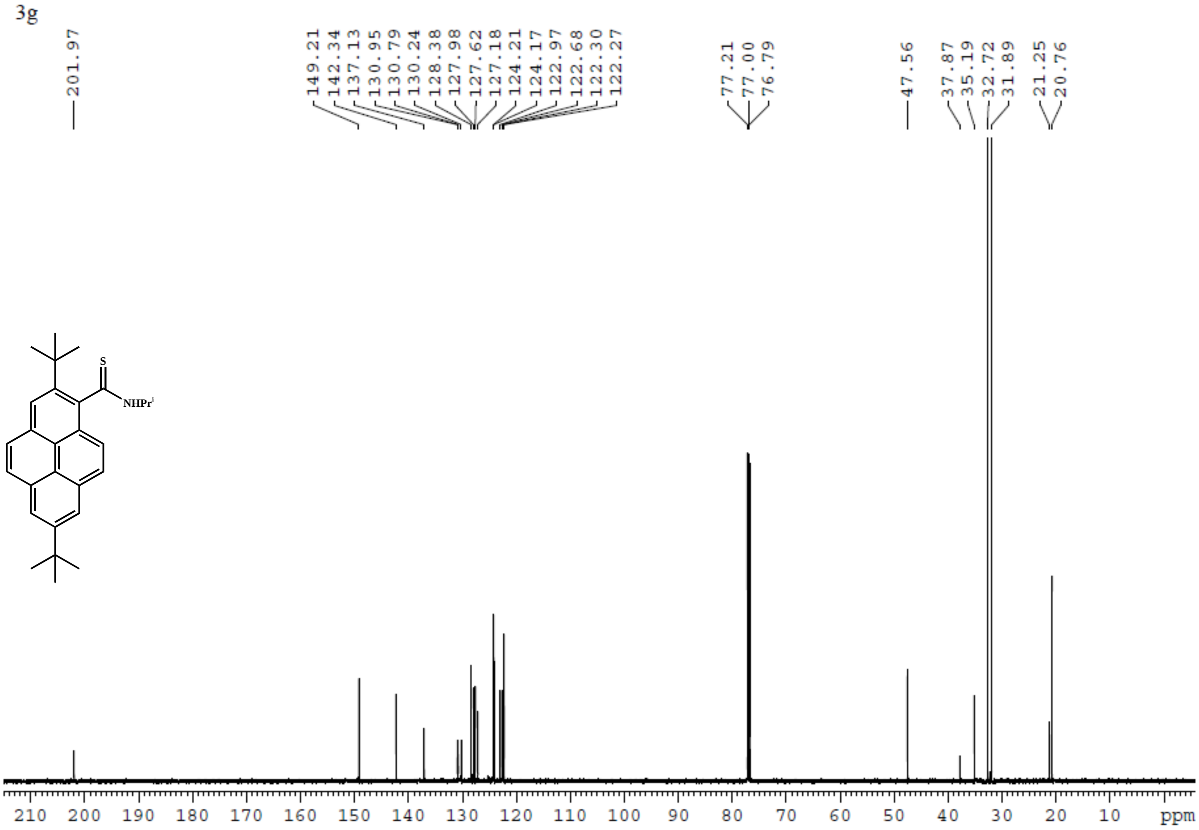

201.97
149.21 142.34 137.13 130.95 130.79 130.24 128.38 127.98 127.62 127.18 124.21 124.17 122.97 122.68 122.30 122.27
77.21 77.00 76.79
47.56
37.87 35.19 32.72 31.89
21.25 20.76
S
NHPr^i
210 200 190 180 170 160 150 140 130 120 110 100 90 80 70 60 50 40 30 20 10 ppm

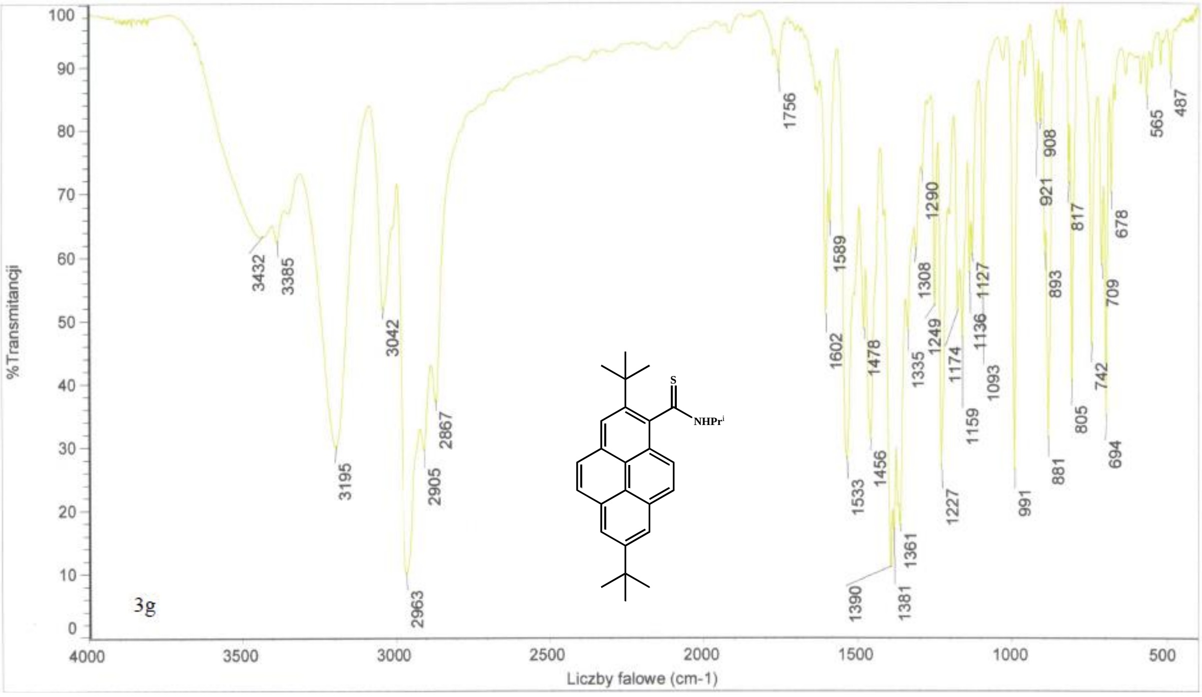

3h

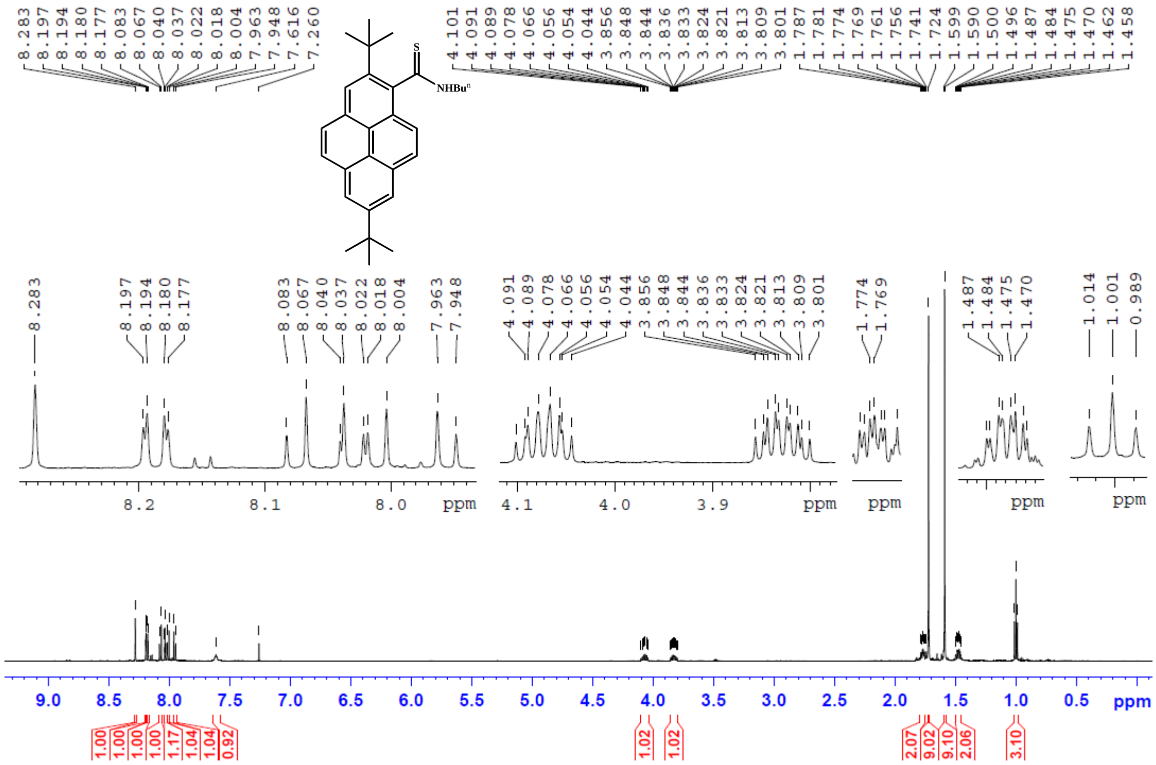

3h

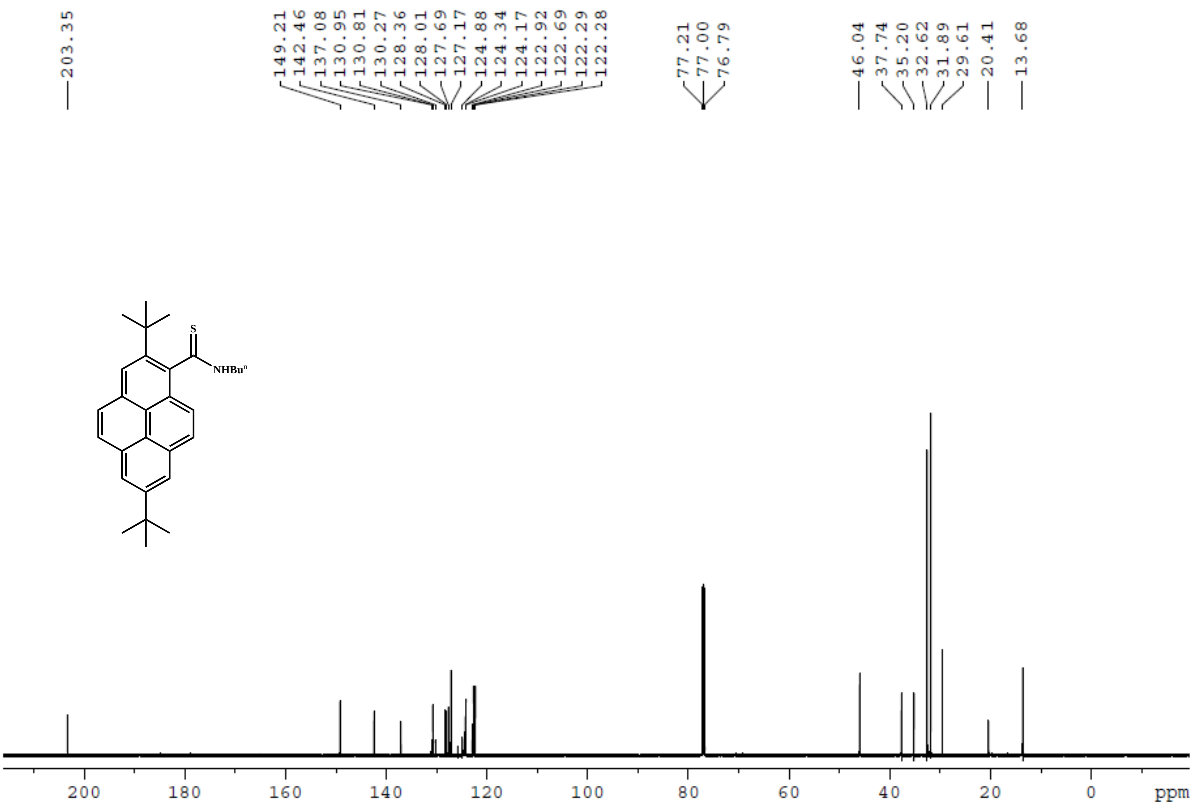
203.35
149.21
142.46
137.08
130.95
130.81
130.27
128.36
128.01
127.69
127.17
124.88
124.34
124.17
122.92
122.69
122.29
122.28
77.21
77.00
76.79
46.04
37.74
35.20
32.62
31.89
29.61
20.41
13.68
S
NHBu^n
200
180
160
140
120
100
80
60
40
20
0
ppm

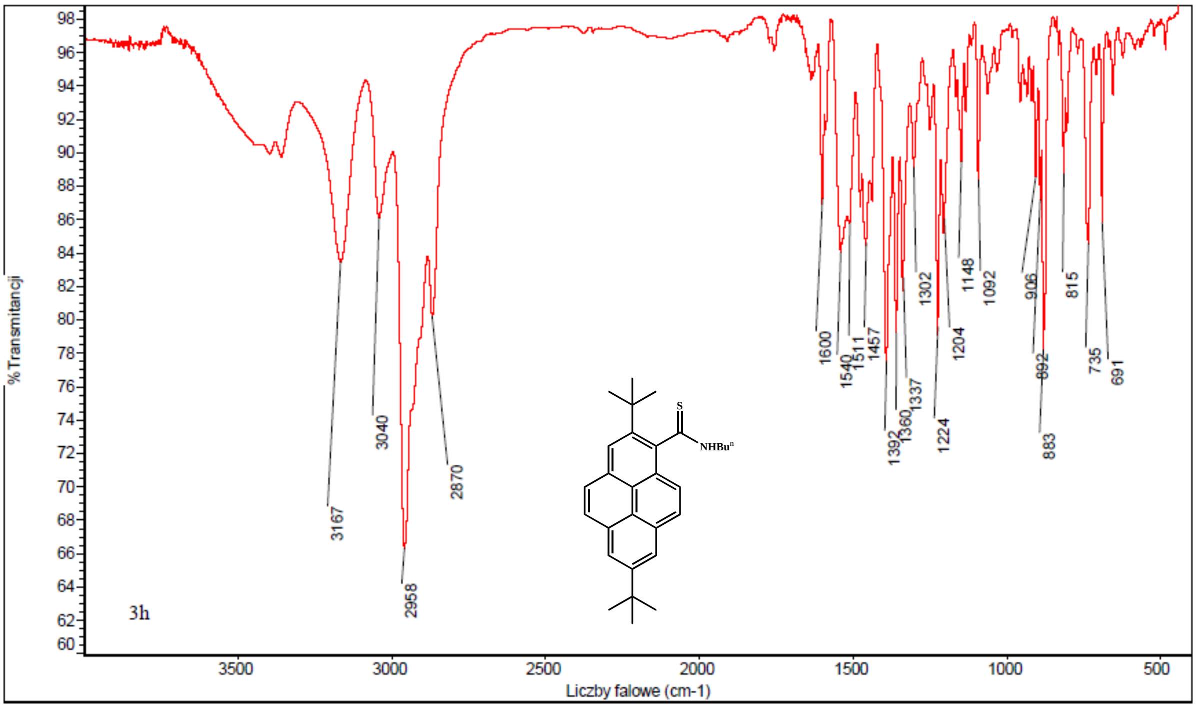
% Transmitancji
Liczby falowe (cm-1)
3h
S
NHBu^n
3167
3040
2958
2870
1600
1540
1511
1457
1392
1360
1337
1302
1224
1204
1148
1092
906
892
883
815
735
691
3500
3000
2500
2000
1500
1000
500

3i

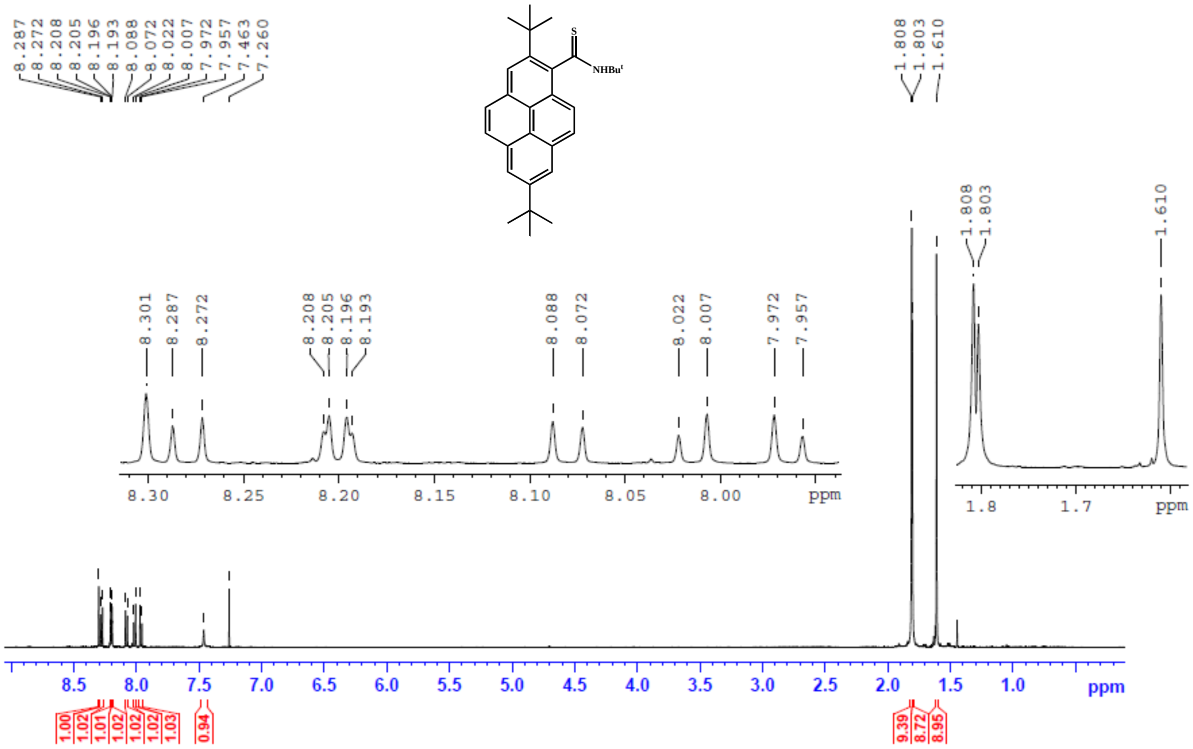
8.287
8.272
8.208
8.205
8.196
8.193
8.088
8.072
8.022
8.007
7.972
7.957
7.463
7.260
1.808
1.803
1.610
NHBu^t
S
8.301
ppm
8.30 8.25 8.20 8.15 8.10 8.05 8.00
1.8 1.7
8.5 8.0 7.5 7.0 6.5 6.0 5.5 5.0 4.5 4.0 3.5 3.0 2.5 2.0 1.5 1.0
1.00 1.02 1.01 1.02 1.02 1.02 1.03 0.94
9.39 8.72 8.95

3i

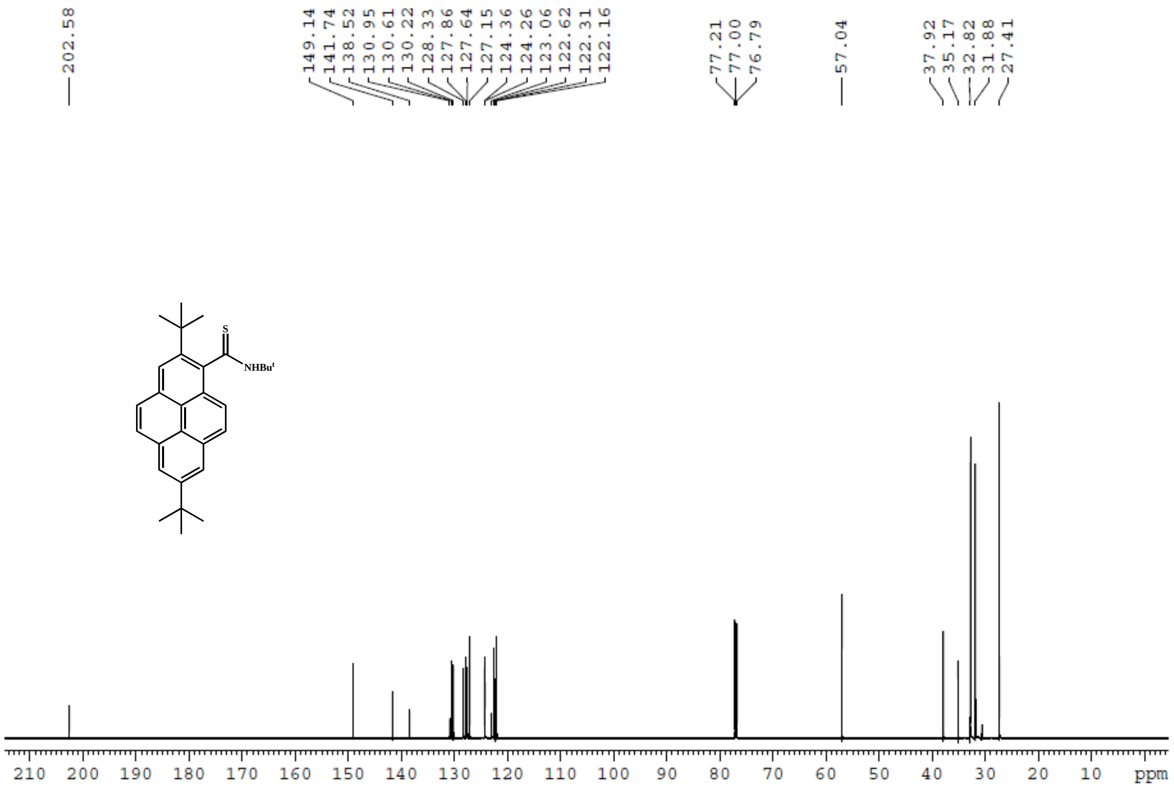
202.58
149.14
141.74
138.52
130.95
130.61
130.22
128.33
127.86
127.64
127.15
124.36
124.26
123.06
122.62
122.31
122.16
77.21
77.00
76.79
57.04
37.92
35.17
32.82
31.88
27.41
S
NHBu^t
210 200 190 180 170 160 150 140 130 120 110 100 90 80 70 60 50 40 30 20 10 ppm

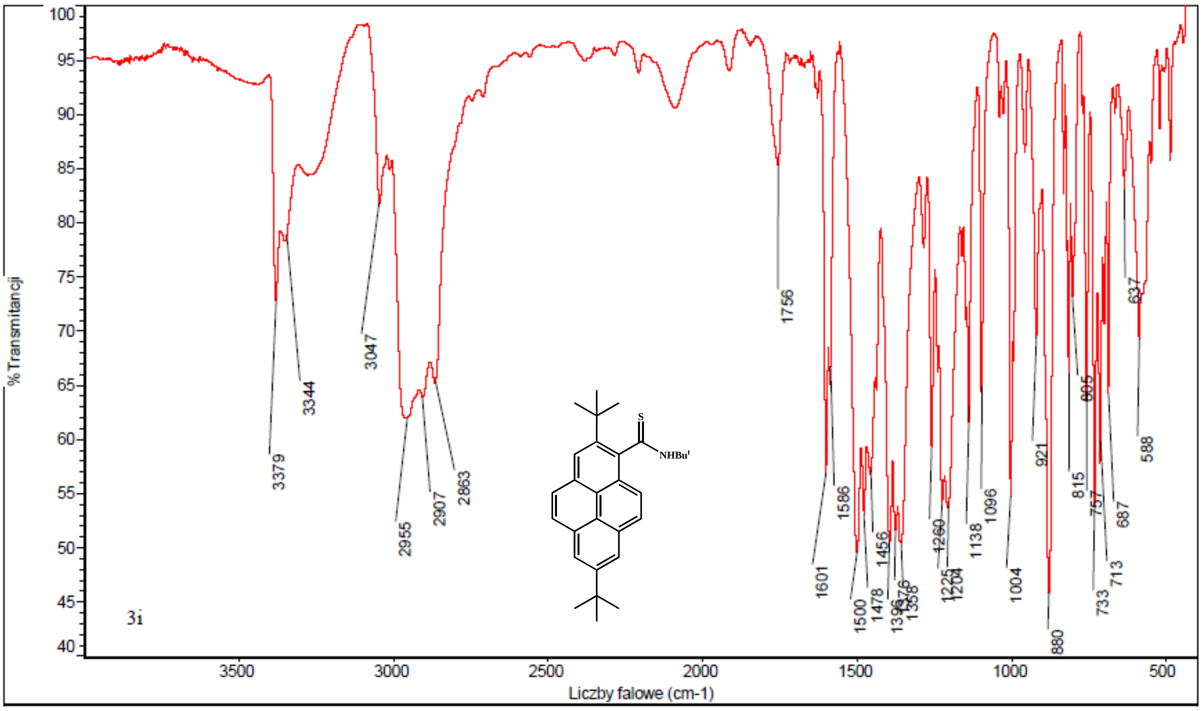
3i
% Transmitancji
Liczby falowe (cm-1)
NHBu^t
S
3379
3344
3047
2955
2907
2863
1756
1601
1586
1500
1478
1456
1396
1376
1358
1260
1225
1204
1138
1096
1004
921
880
815
805
757
733
713
687
637
588

3j

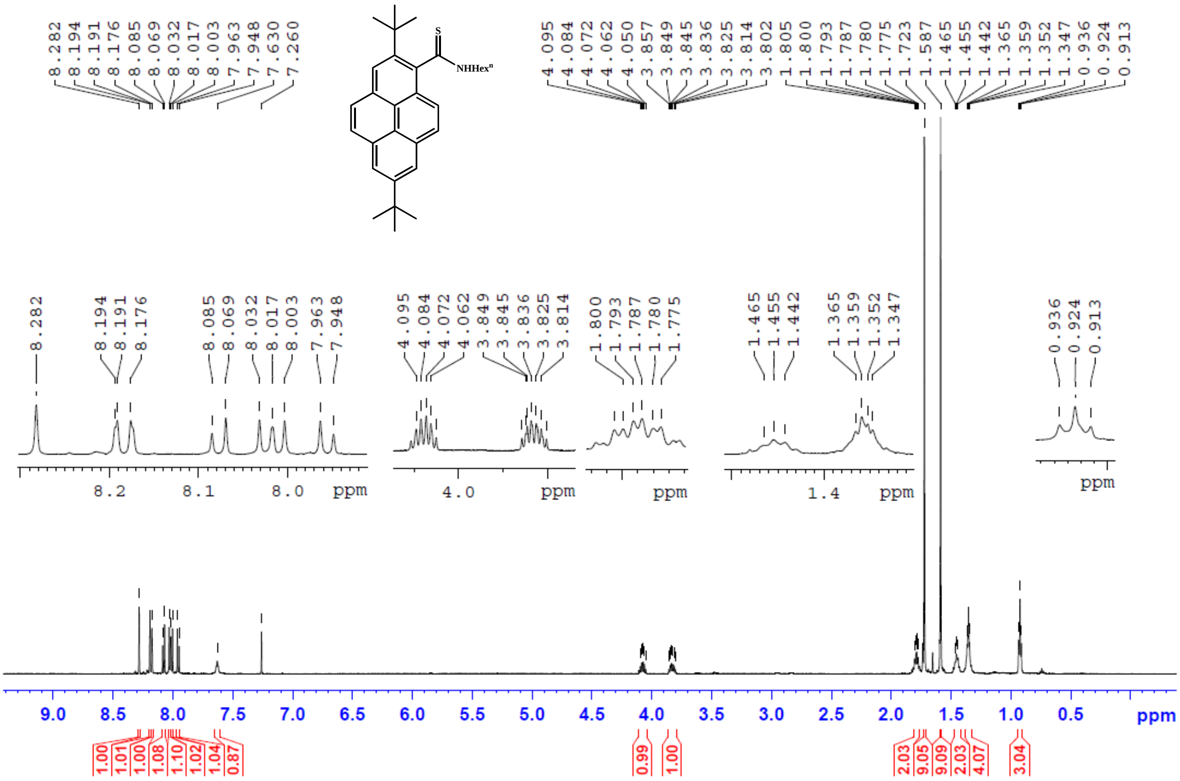
NHHex^n
S
8.282
8.194
8.191
8.176
8.085
8.069
8.032
8.017
8.003
7.963
7.948
7.630
7.260
4.095
4.084
4.072
4.062
4.050
3.857
3.849
3.845
3.836
3.825
3.814
3.802
1.805
1.800
1.793
1.787
1.780
1.775
1.723
1.587
1.465
1.455
1.442
1.365
1.359
1.352
1.347
0.936
0.924
0.913
ppm
1.00
1.01
1.00
1.08
1.10
1.02
1.04
0.87
0.99
1.00
2.03
9.05
9.09
2.03
4.07
3.04

3j

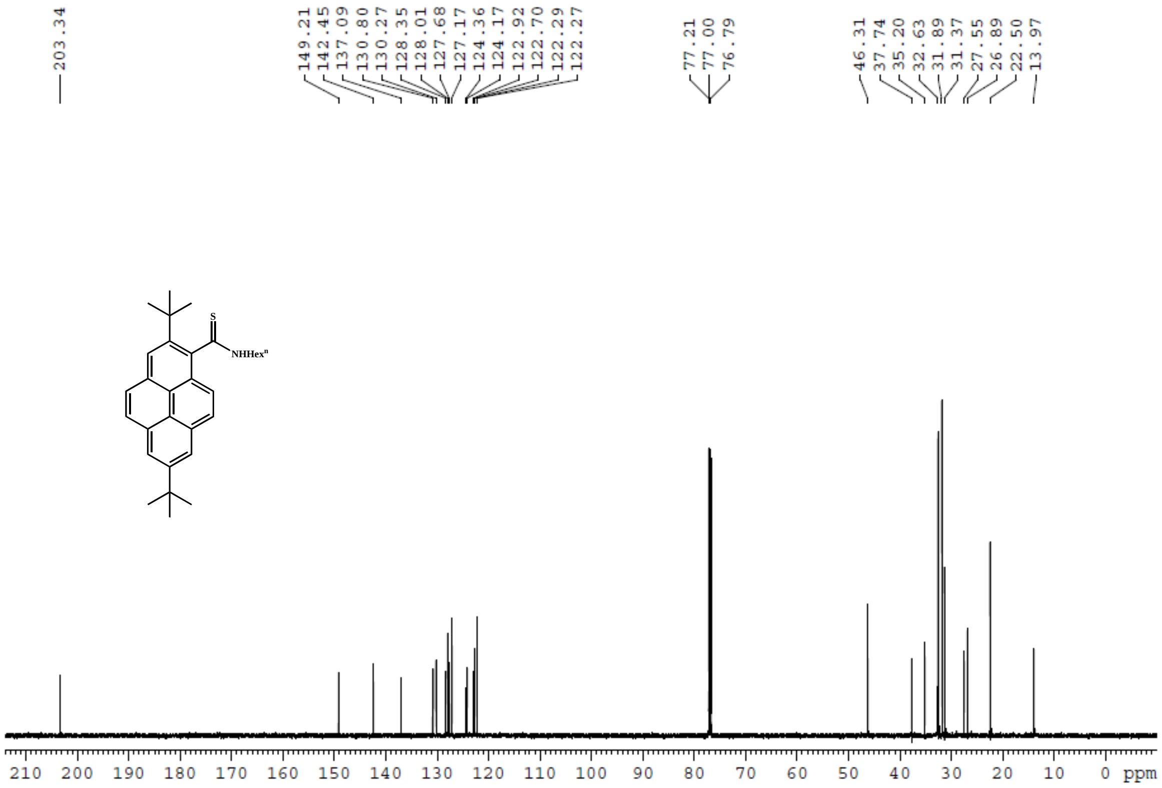
203.34
149.21
142.45
137.09
130.80
130.27
128.35
128.01
127.68
127.17
124.36
124.17
122.92
122.70
122.29
122.27
77.21
77.00
76.79
46.31
37.74
35.20
32.63
31.89
31.37
27.55
26.89
22.50
13.97
S
NHHex^n
ppm

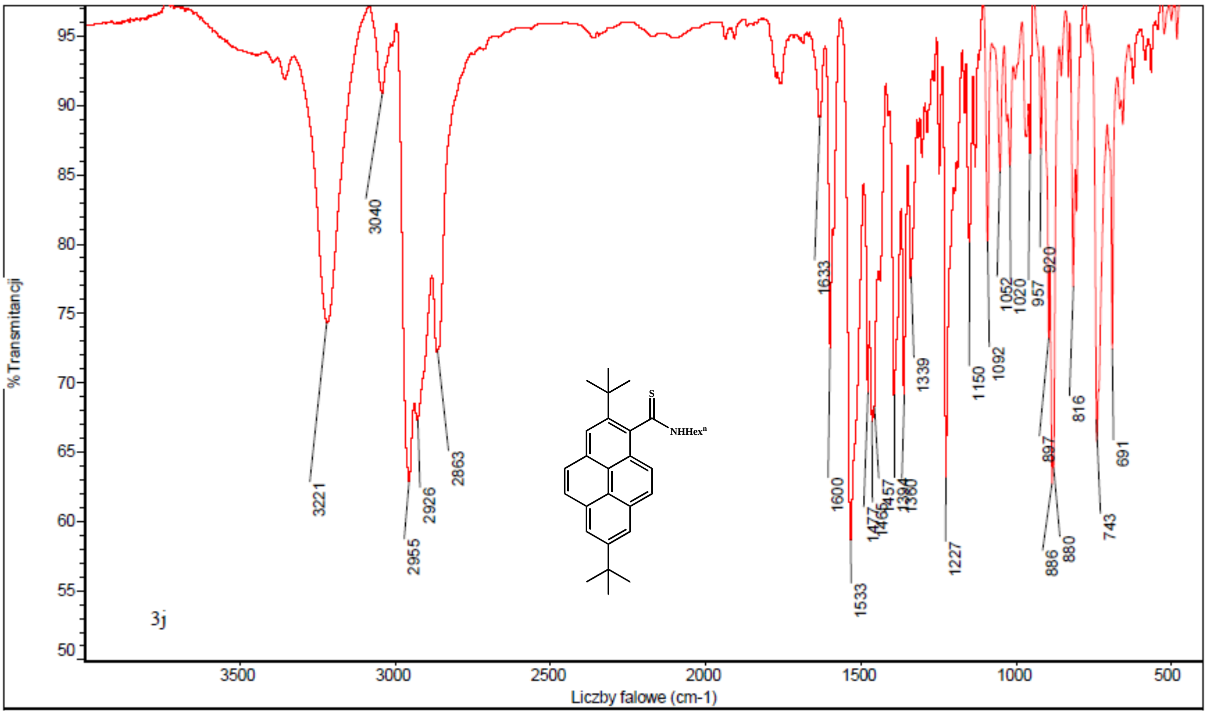
% Transmitancji
Liczby falowe (cm-1)
3j
S
NHHex^n
3221
3040
2955
2926
2863
1633
1600
1533
1477
1466
1457
1394
1380
1339
1227
1150
1092
1052
1020
957
920
897
886
880
816
743
691

3k

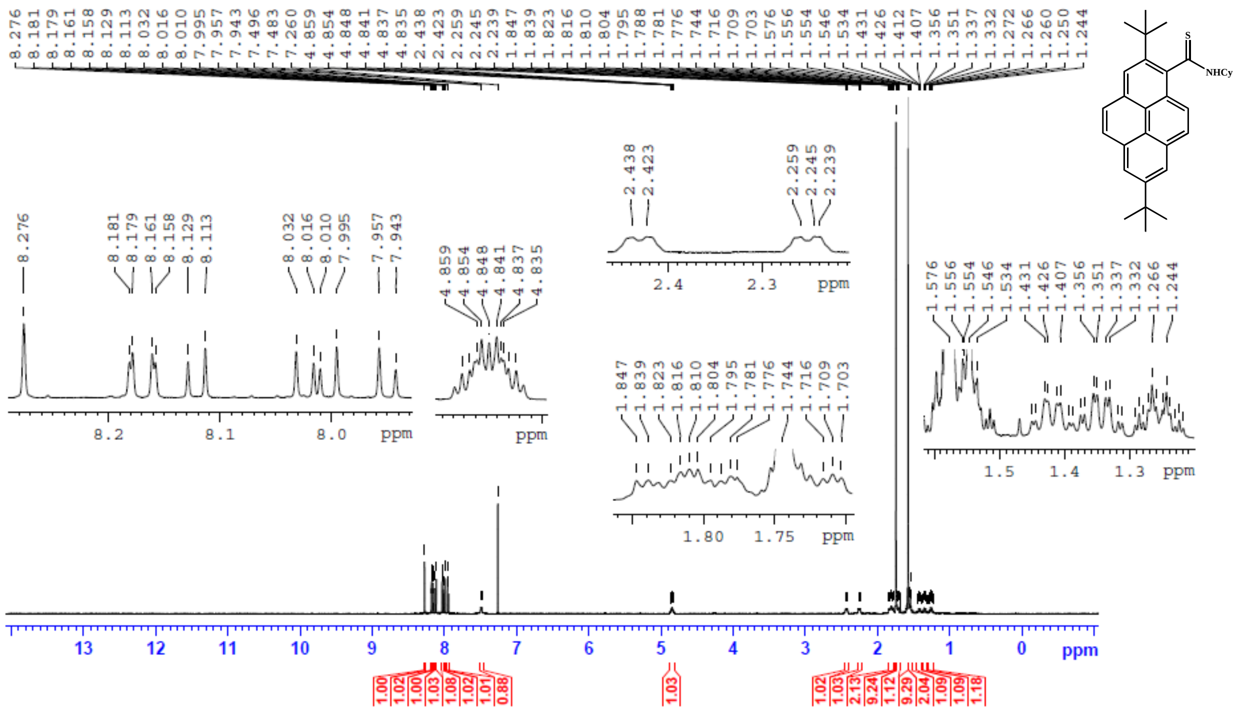

3k

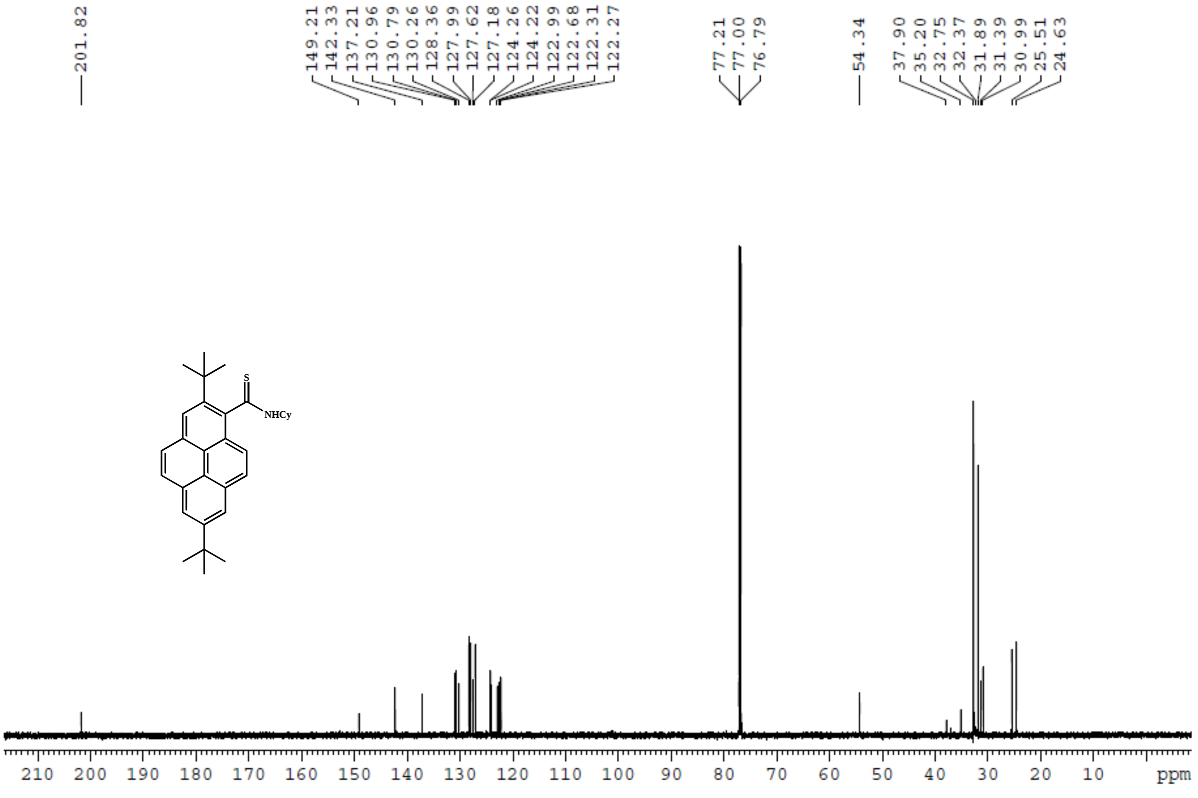

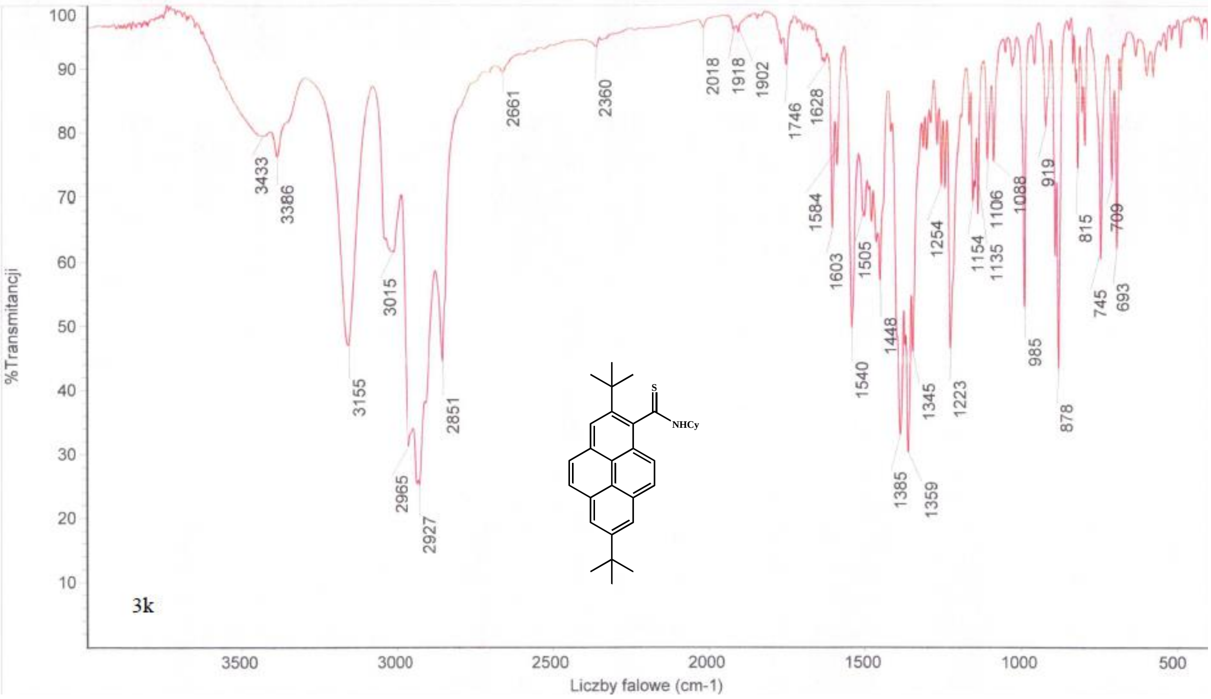

3k
%Transmitancji
Liczby falowe (cm-1)
100
90
80
70
60
50
40
30
20
10
3500
3000
2500
2000
1500
1000
500
3433
3386
3155
3015
2965
2927
2851
2661
2360
2018
1918
1902
1746
1628
1584
1603
1540
1505
1448
1385
1359
1345
1254
1223
1154
1135
1106
1088
985
919
878
815
745
709
693
S
NHCy

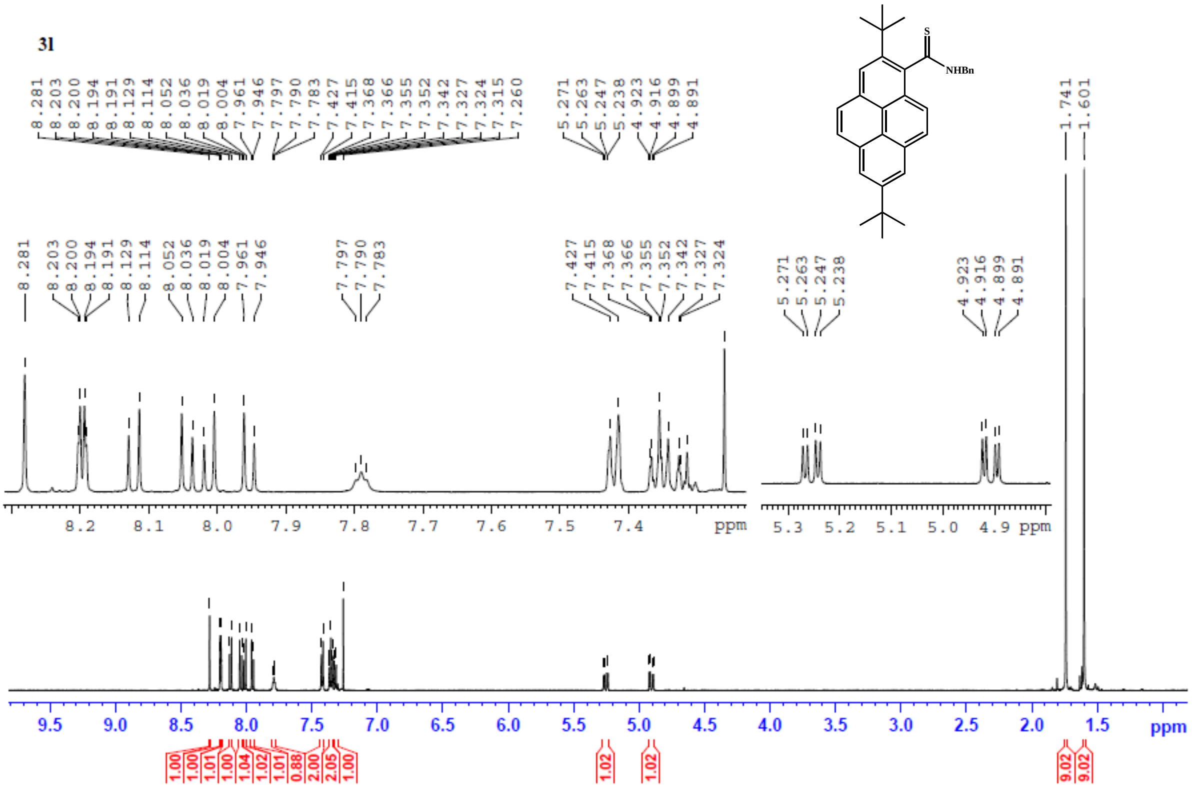

3l
S
NHBn
8.281
8.203
8.200
8.194
8.191
8.129
8.114
8.052
8.036
8.019
8.004
7.961
7.946
7.797
7.790
7.783
7.427
7.415
7.368
7.366
7.355
7.352
7.342
7.327
7.324
7.315
7.260
5.271
5.263
5.247
5.238
4.923
4.916
4.899
4.891
1.741
1.601
ppm
1.00
1.00
1.01
1.00
1.04
1.02
1.01
0.88
2.00
2.05
1.00
1.02
1.02
9.02
9.02

31

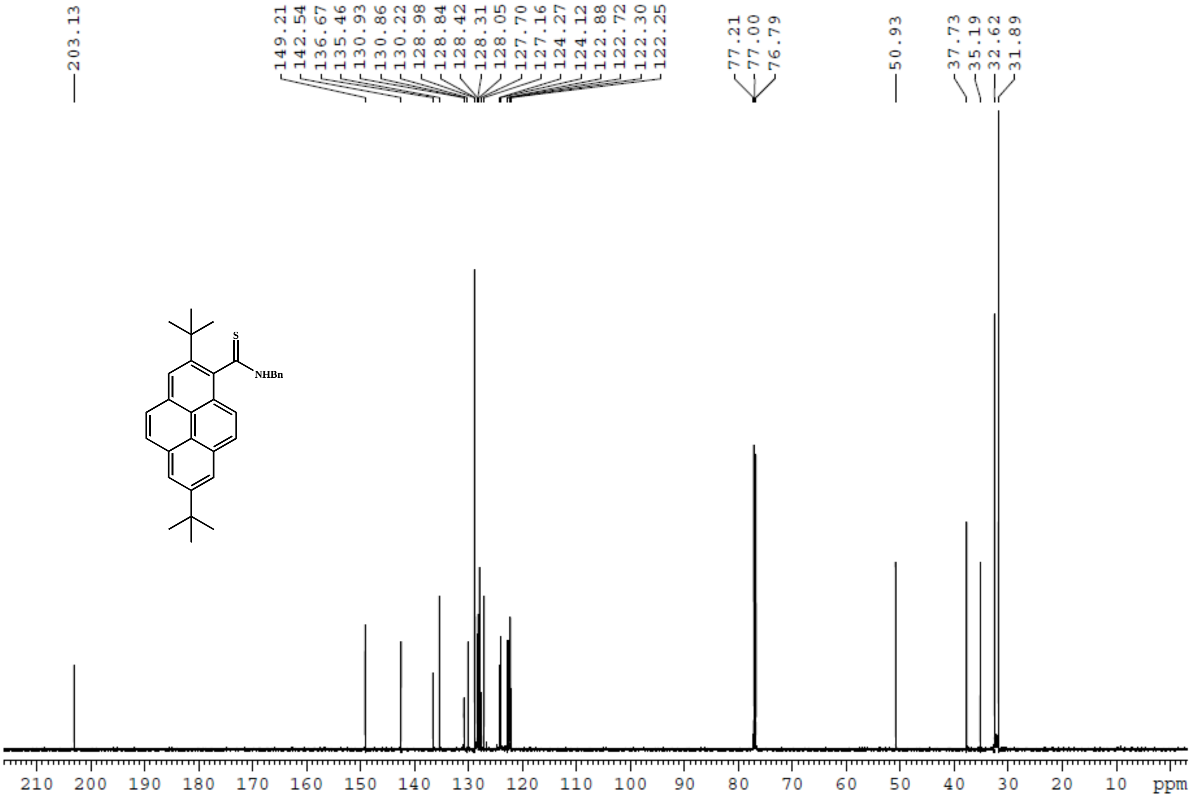

203.13
149.21
142.54
136.67
135.46
130.93
130.86
130.22
128.98
128.84
128.42
128.31
128.05
127.70
127.16
124.27
124.12
122.88
122.72
122.30
122.25
77.21
77.00
76.79
50.93
37.73
35.19
32.62
31.89
S
NHBn
210 200 190 180 170 160 150 140 130 120 110 100 90 80 70 60 50 40 30 20 10 ppm

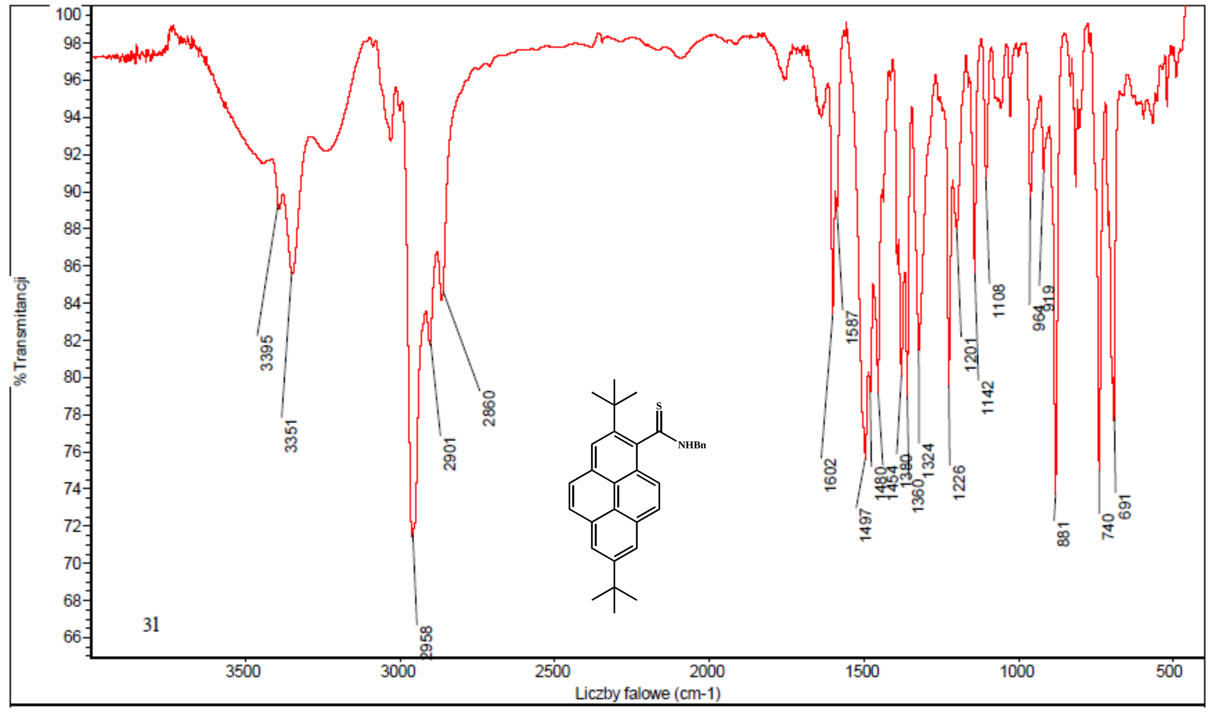

% Transmitancji
Liczby falowe (cm-1)
31
S
NHBn
3395
3351
2958
2901
2860
1602
1587
1497
1480
1454
1380
1360
1324
1226
1201
1142
1108
964
919
881
740
691

3m

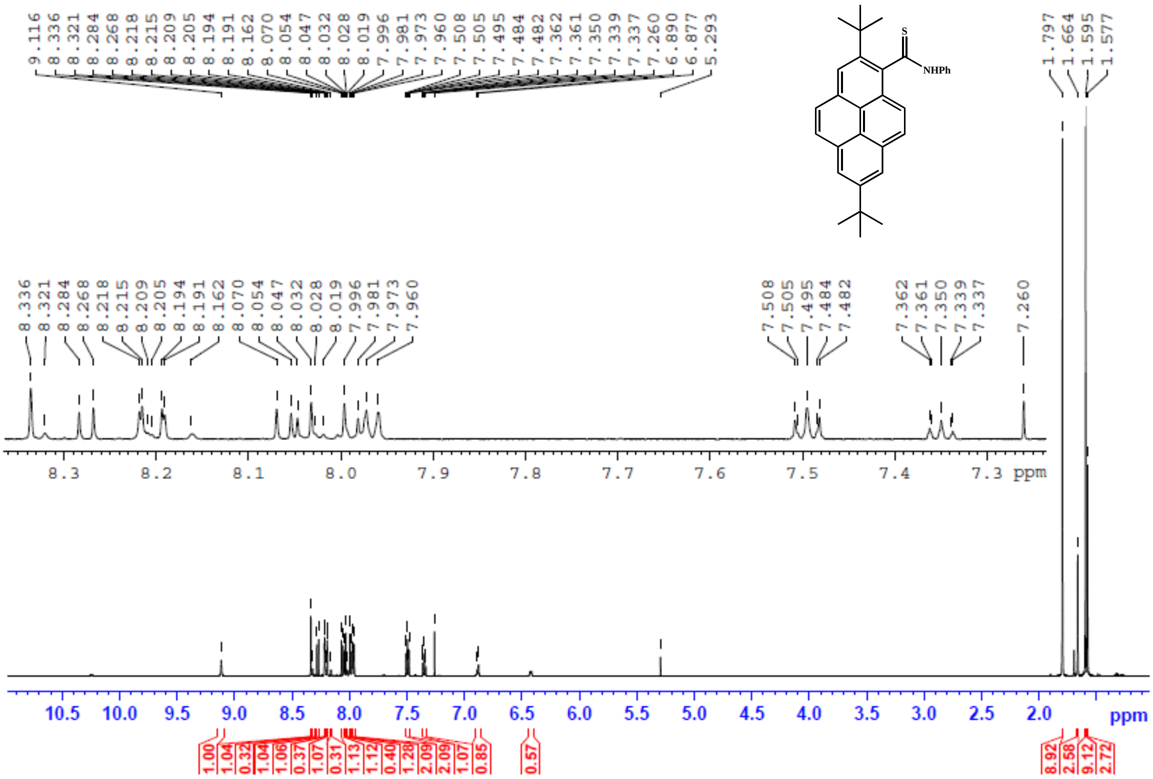
9.116 8.336 8.321 8.284 8.268 8.218 8.215 8.209 8.205 8.194 8.191 8.162 8.070 8.054 8.047 8.032 8.028 8.019 7.996 7.981 7.973 7.960 7.508 7.505 7.495 7.484 7.482 7.362 7.361 7.350 7.339 7.337 7.260 6.890 6.877 5.293
1.797 1.664 1.595 1.577
S
NHPh
8.336 8.321 8.284 8.268 8.218 8.215 8.209 8.205 8.194 8.191 8.162 8.070 8.054 8.047 8.032 8.028 8.019 7.996 7.981 7.973 7.960
7.508 7.505 7.495 7.484 7.482
7.362 7.361 7.350 7.339 7.337
7.260
8.3 8.2 8.1 8.0 7.9 7.8 7.7 7.6 7.5 7.4 7.3 ppm
10.5 10.0 9.5 9.0 8.5 8.0 7.5 7.0 6.5 6.0 5.5 5.0 4.5 4.0 3.5 3.0 2.5 2.0 ppm
1.00 1.04 0.32 1.04 1.06 0.37 1.07 0.31 1.13 1.12 0.40 1.28 2.09 2.09 1.07 0.85 0.57
8.92 2.58 9.12 2.72

3m

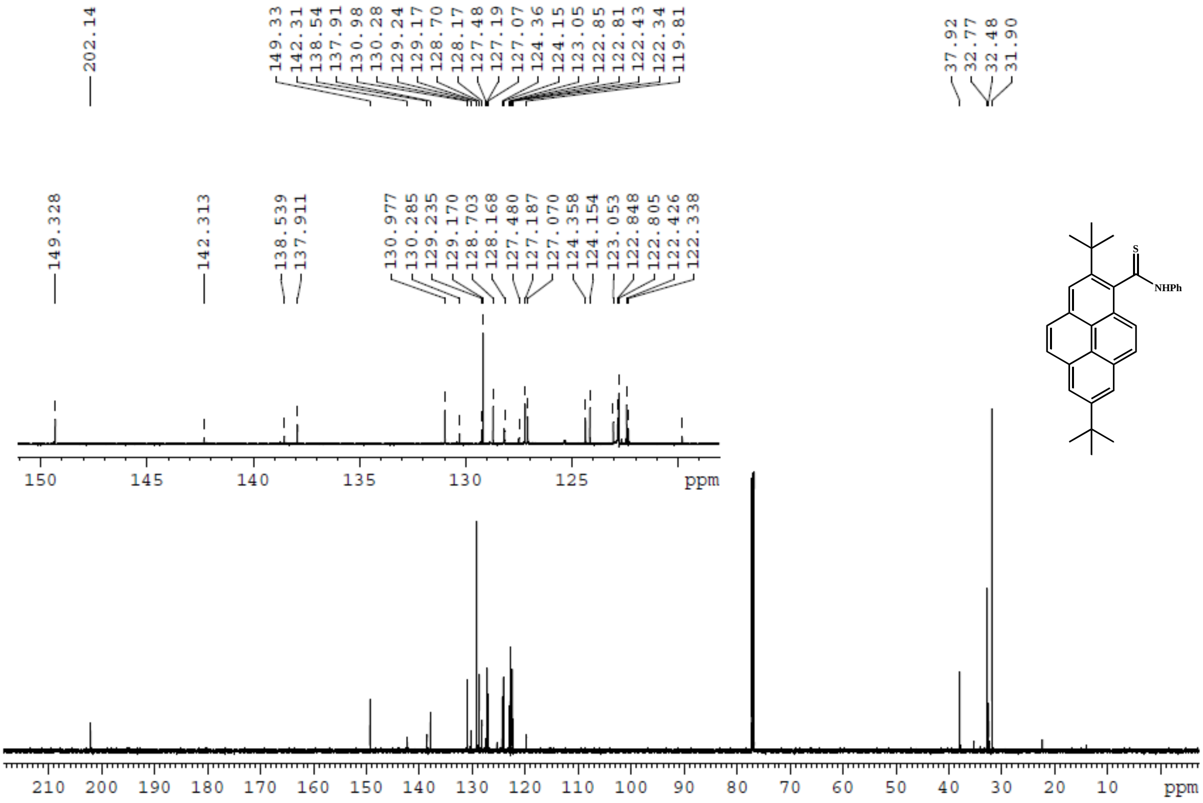
202.14
149.33 142.31 138.54 137.91 130.98 130.28 129.24 129.17 128.70 128.17 127.48 127.19 127.07 124.36 124.15 123.05 122.85 122.81 122.43 122.34 119.81
37.92 32.77 32.48 31.90
149.328
142.313
138.539 137.911
130.977 130.285 129.235 129.170 128.703 128.168 127.480 127.187 127.070 124.358 124.154 123.053 122.848 122.805 122.426 122.338
S
NHPh
150 145 140 135 130 125 ppm
210 200 190 180 170 160 150 140 130 120 110 100 90 80 70 60 50 40 30 20 10 ppm

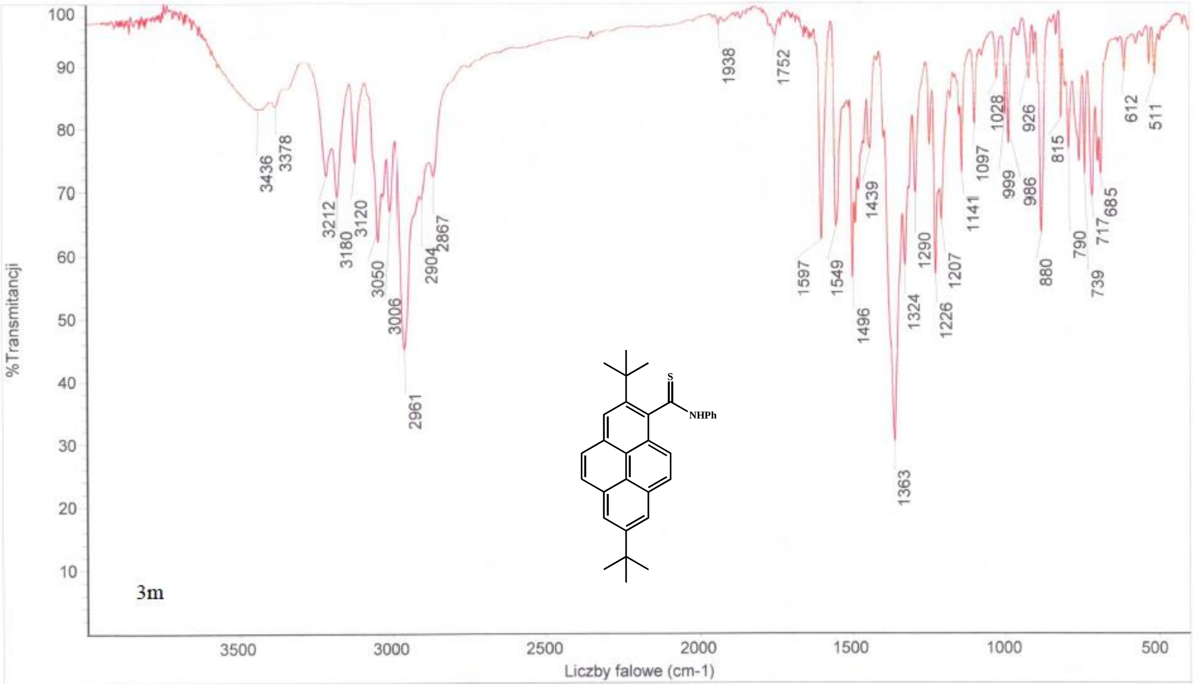
3m
%Transmitancji
Liczby falowe (cm-1)
3436
3378
3212
3180
3120
3050
3006
2961
2904
2867
1938
1752
1597
1549
1496
1439
1363
1324
1290
1226
1207
1141
1097
1028
999
986
926
880
815
790
739
717
685
612
511
S
NHPh

3n

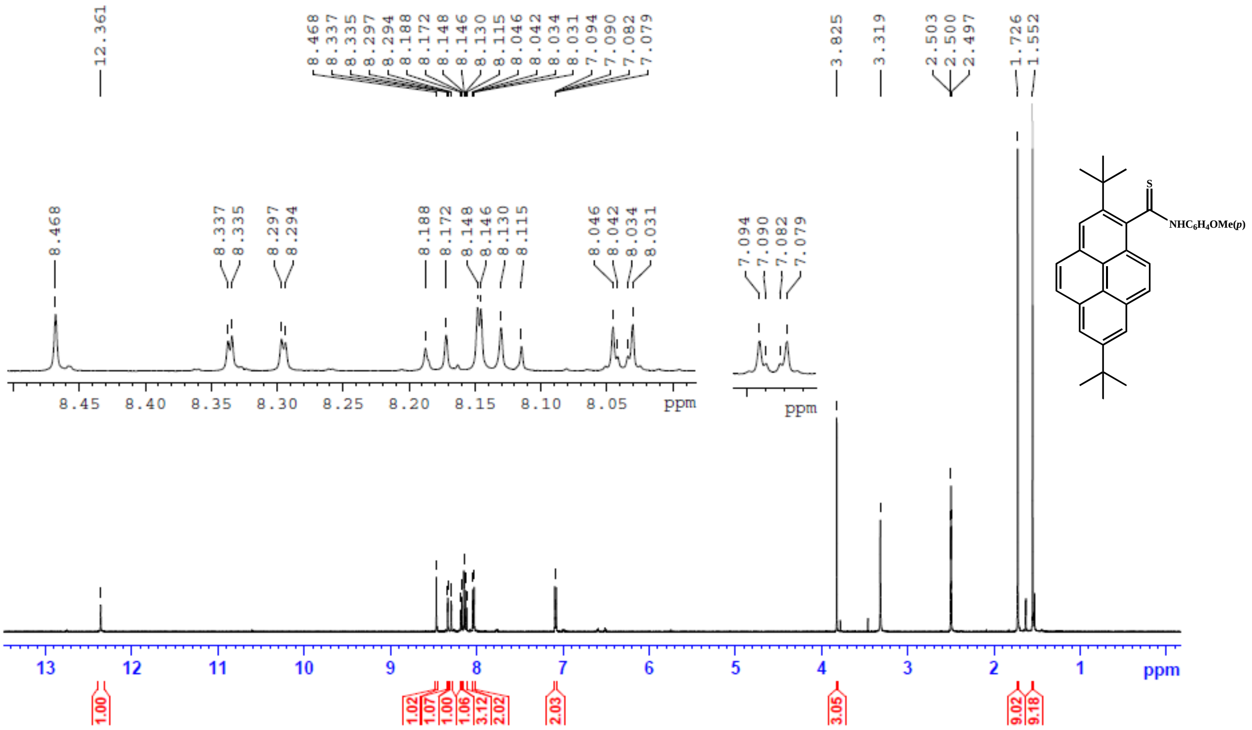
12.361
8.468
8.337
8.335
8.297
8.294
8.188
8.172
8.148
8.146
8.130
8.115
8.046
8.042
8.034
8.031
7.094
7.090
7.082
7.079
3.825
3.319
2.503
2.500
2.497
1.726
1.552
ppm
S
NHC6H4OMe(p)
1.00
1.02
1.07
1.00
1.06
3.12
2.02
2.03
3.05
9.02
9.18

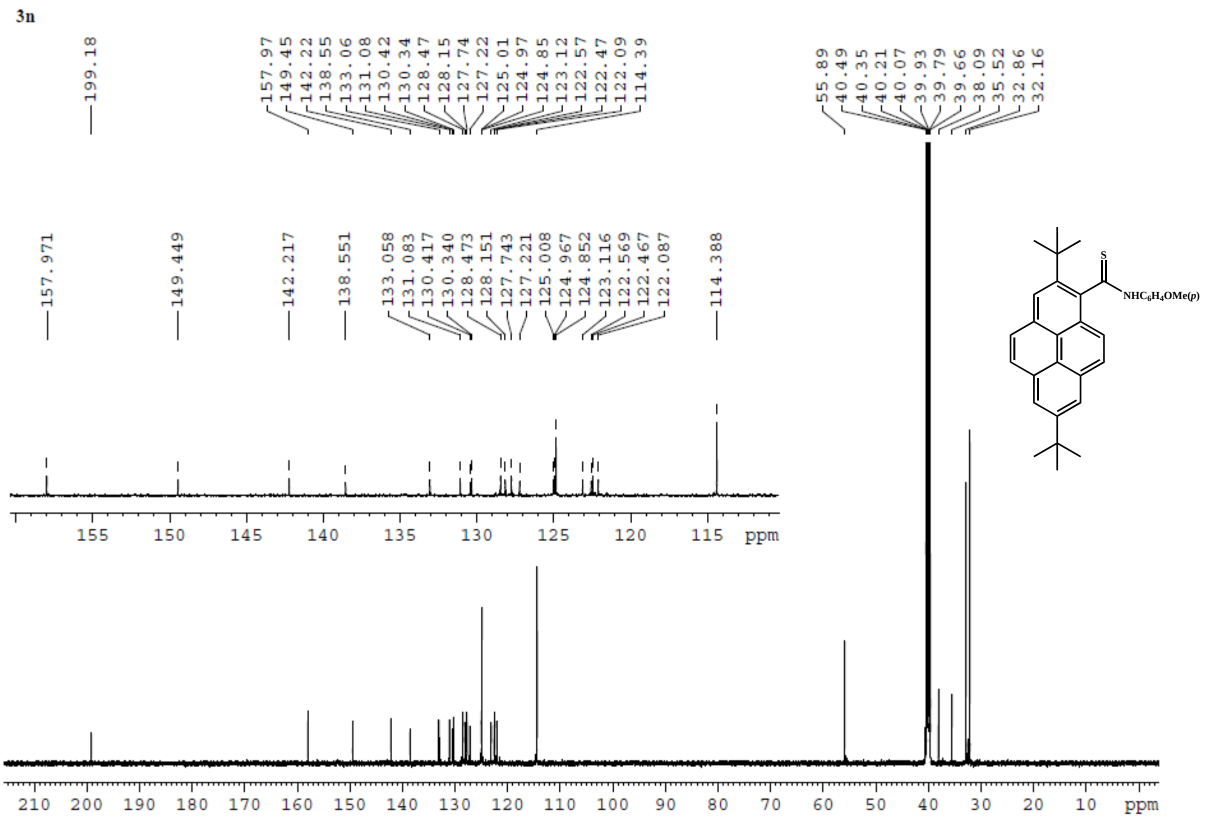
3n
199.18
157.97 149.45 142.22 138.55 133.06 131.08 130.42 130.34 128.47 128.15 127.74 127.22 125.01 124.97 124.85 123.12 122.57 122.47 122.09 114.39
55.89 40.49 40.35 40.21 40.07 39.93 39.79 39.66 38.09 35.52 32.86 32.16
157.971 149.449 142.217 138.551 133.058 131.083 130.417 130.340 128.473 128.151 127.743 127.221 125.008 124.967 124.852 123.116 122.569 122.467 122.087 114.388
155 150 145 140 135 130 125 120 115 ppm
210 200 190 180 170 160 150 140 130 120 110 100 90 80 70 60 50 40 30 20 10 ppm
S
$NHC_6H_4OMe(p)$

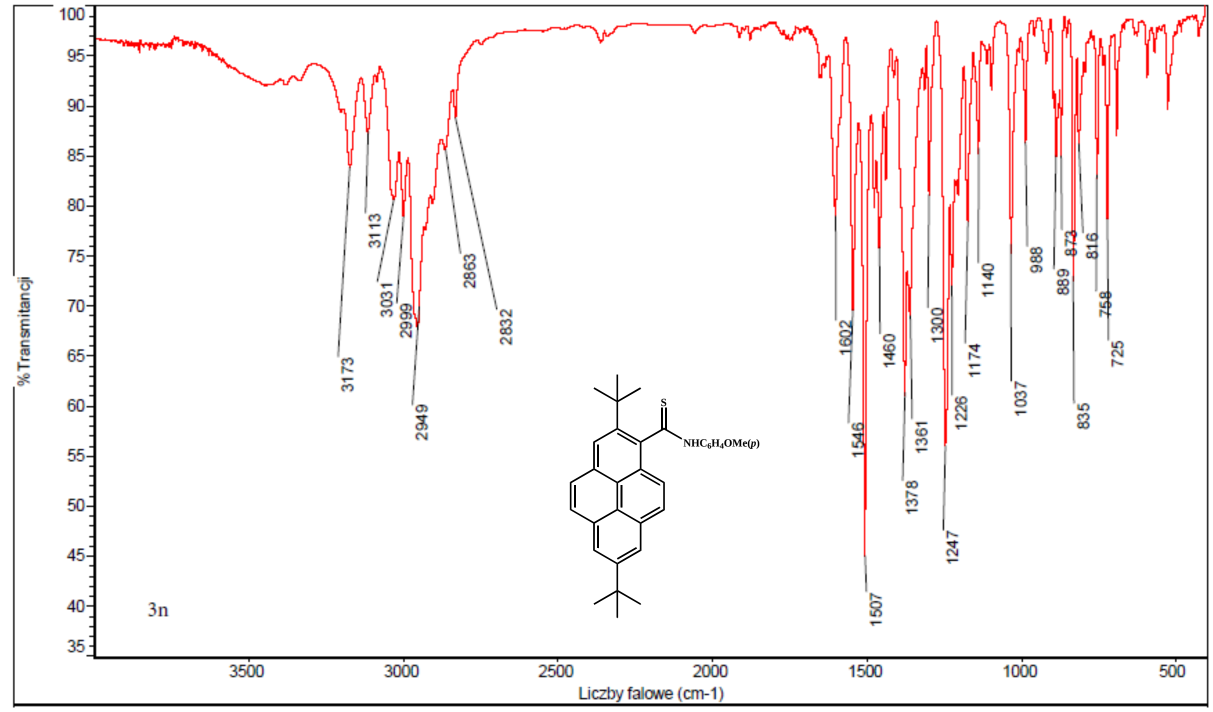
% Transmitancji
Liczby falowe (cm-1)
3n
S
$NHC_6H_4OMe(p)$
3173 3113 3031 2999 2949 2863 2832 1602 1546 1507 1460 1378 1361 1300 1247 1226 1174 1140 1037 988 889 873 835 816 758 725

3o

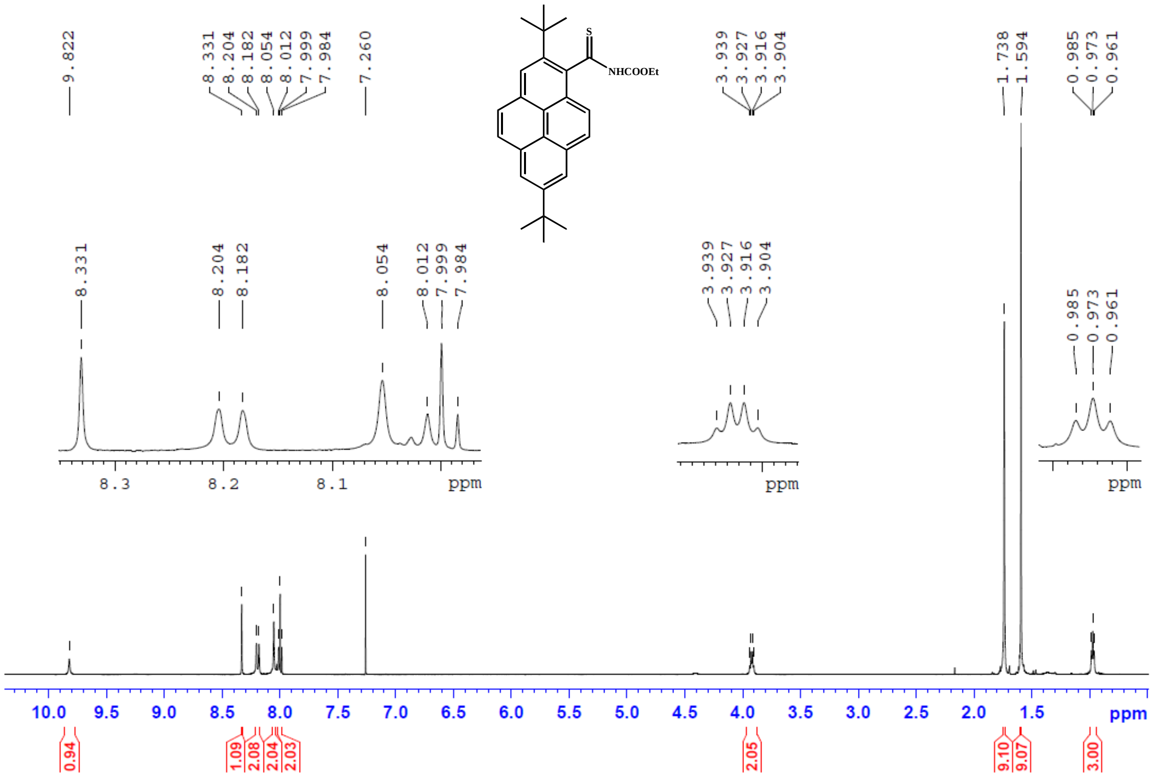
9.822
8.331
8.204
8.182
8.054
8.012
7.999
7.984
7.260
3.939
3.927
3.916
3.904
1.738
1.594
0.985
0.973
0.961
S
NHCOOEt
8.3
8.2
8.1
ppm
0.94
1.09
2.08
2.04
2.03
2.05
9.10
9.07
3.00
10.0
9.5
9.0
8.5
8.0
7.5
7.0
6.5
6.0
5.5
5.0
4.5
4.0
3.5
3.0
2.5
2.0
1.5

3o

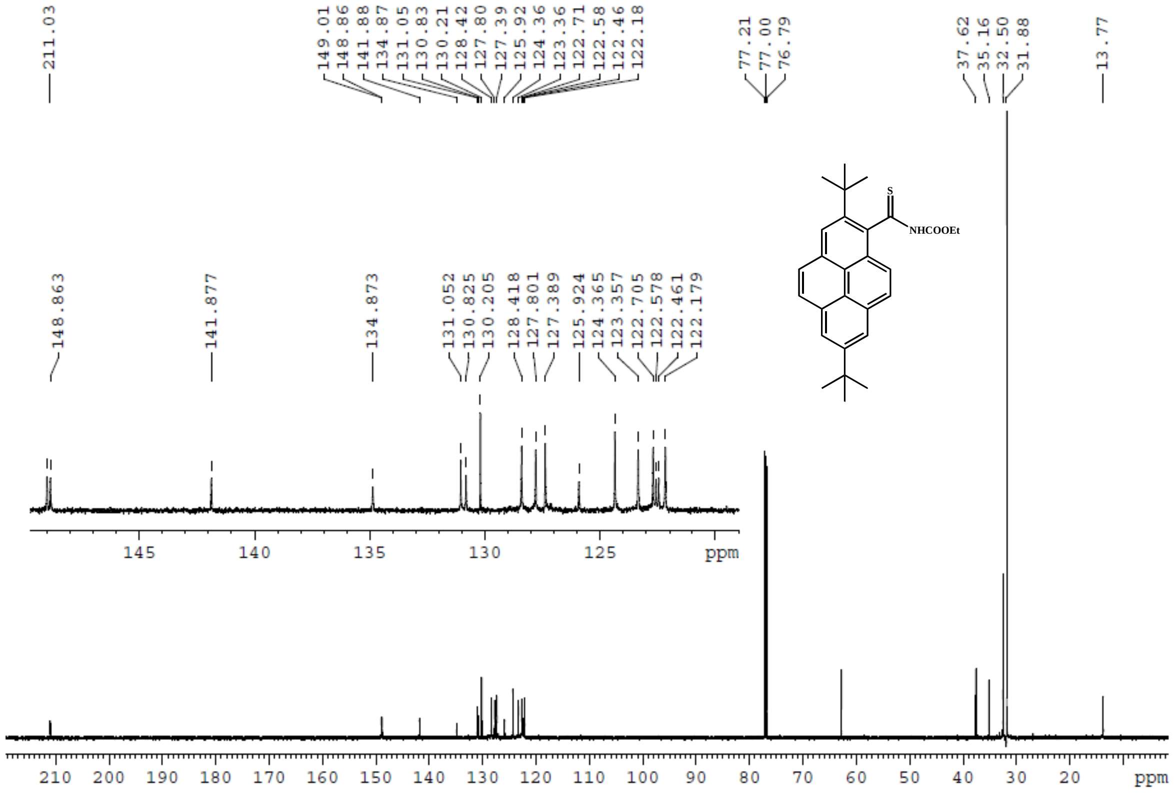
211.03
149.01
148.86
141.88
134.87
131.05
130.83
130.21
128.42
127.80
127.39
125.92
124.36
123.36
122.71
122.58
122.46
122.18
77.21
77.00
76.79
37.62
35.16
32.50
31.88
13.77
148.863
141.877
134.873
131.052
130.825
130.205
128.418
127.801
127.389
125.924
124.365
123.357
122.705
122.578
122.461
122.179
S
NHCOOEt
145
140
135
130
125
ppm
210
200
190
180
170
160
150
140
130
120
110
100
90
80
70
60
50
40
30
20

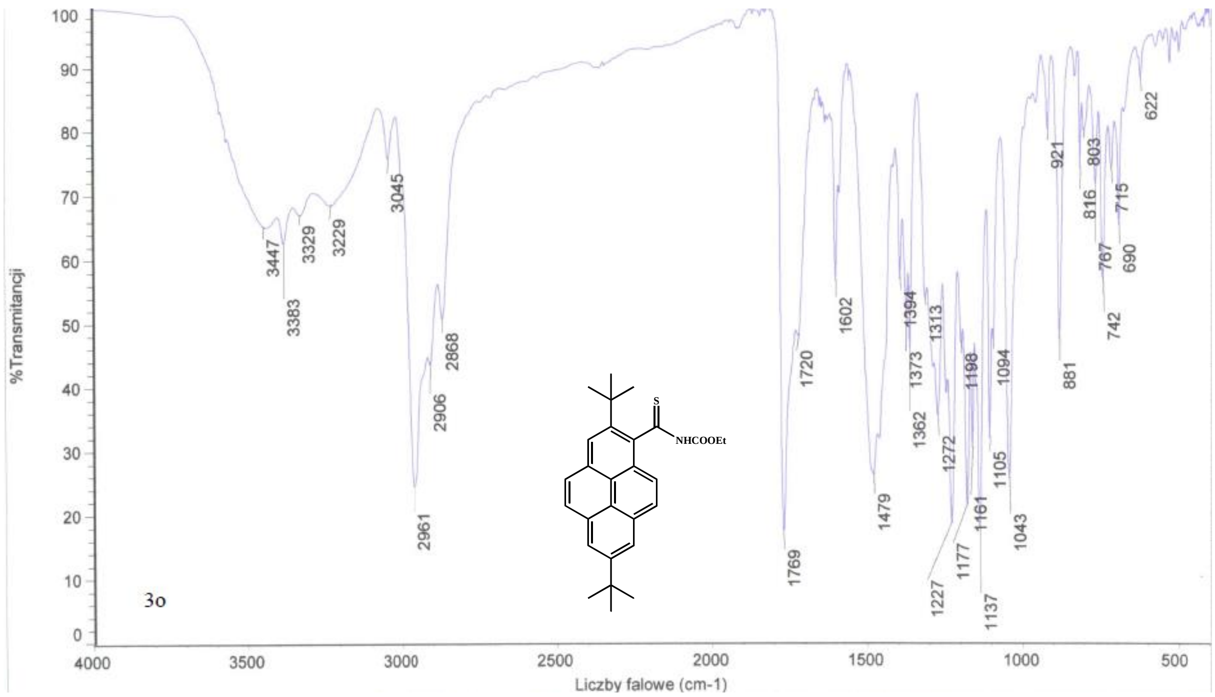
%Transmitancji
Liczby falowe (cm-1)
3o
3447
3383
3329
3229
3045
2961
2906
2868
1769
1720
1602
1479
1394
1373
1362
1313
1272
1227
1198
1177
1161
1137
1105
1094
1043
921
881
816
803
767
742
715
690
622
NHCOOEt
S

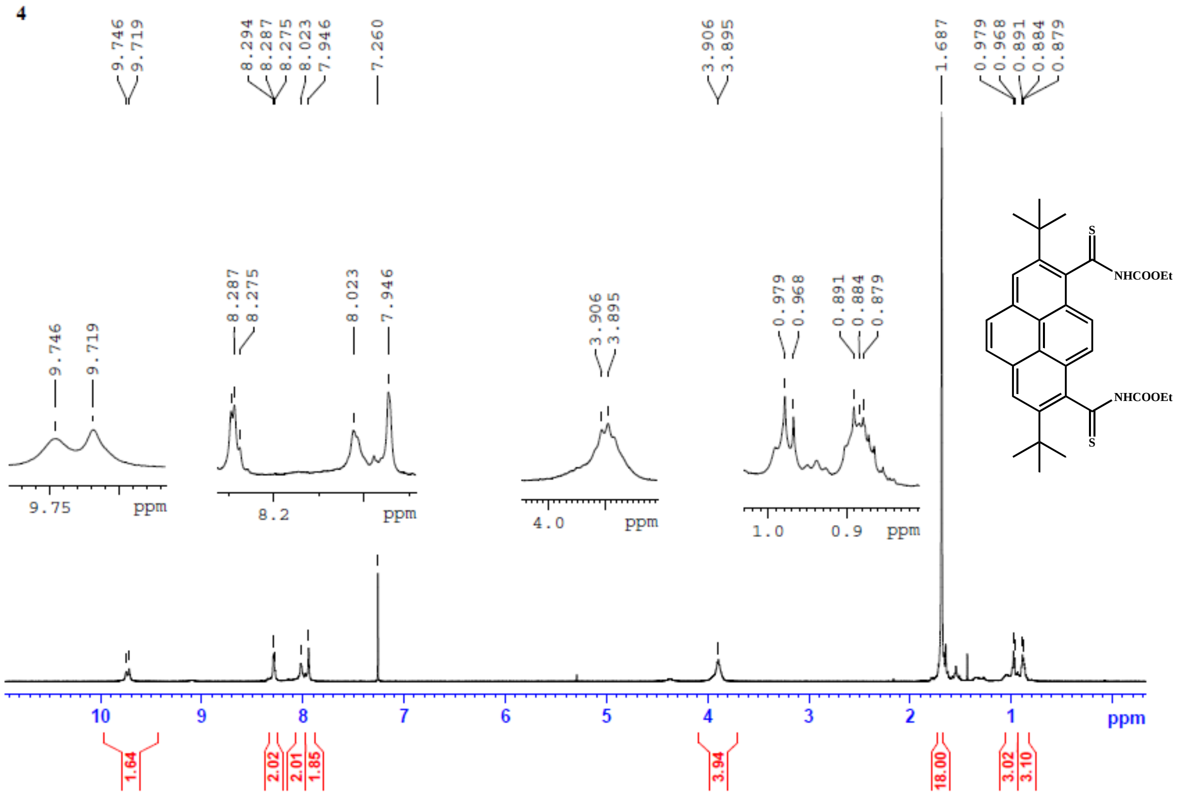
4
9.746
9.719
8.294
8.287
8.275
8.023
7.946
7.260
3.906
3.895
1.687
0.979
0.968
0.891
0.884
0.879
ppm
1.64
2.02
2.01
1.85
3.94
18.00
3.02
3.10
NHCOOEt
NHCOOEt
S
S

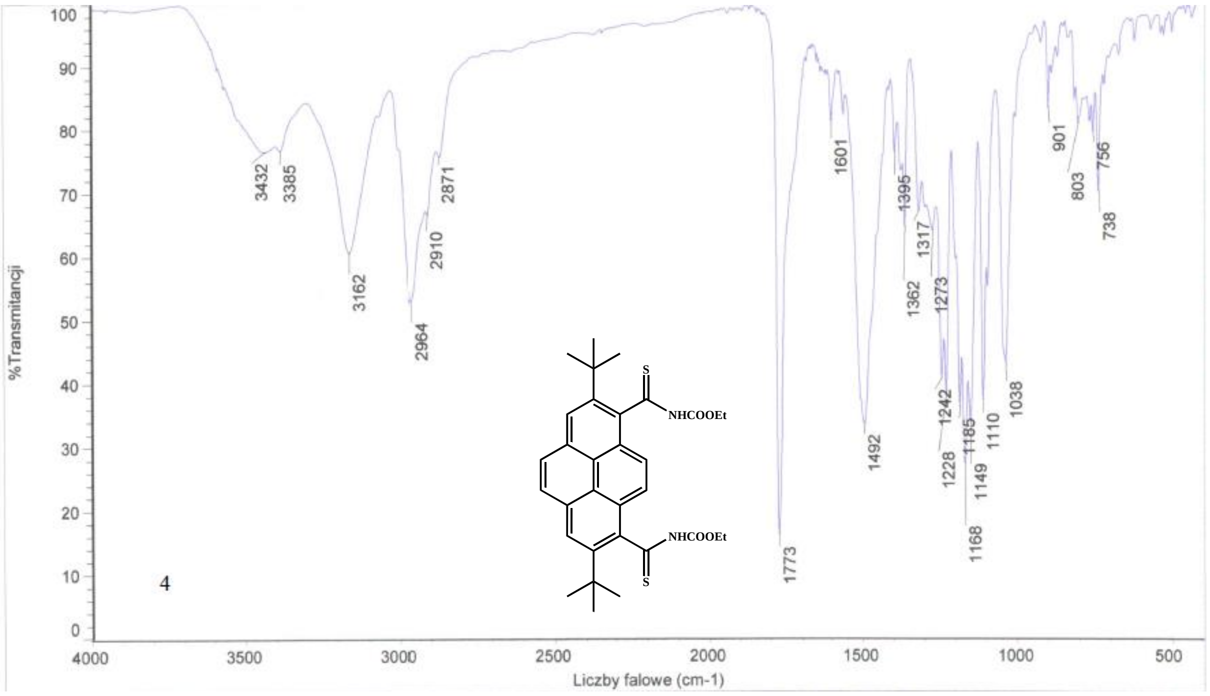

% Transmitancji
Liczby falowe (cm-1)
4
NHCOOEt
NHCOOEt
S
S
3432
3385
3162
2964
2910
2871
1773
1601
1492
1395
1362
1317
1273
1242
1228
1185
1168
1149
1110
1038
901
803
756
738

5

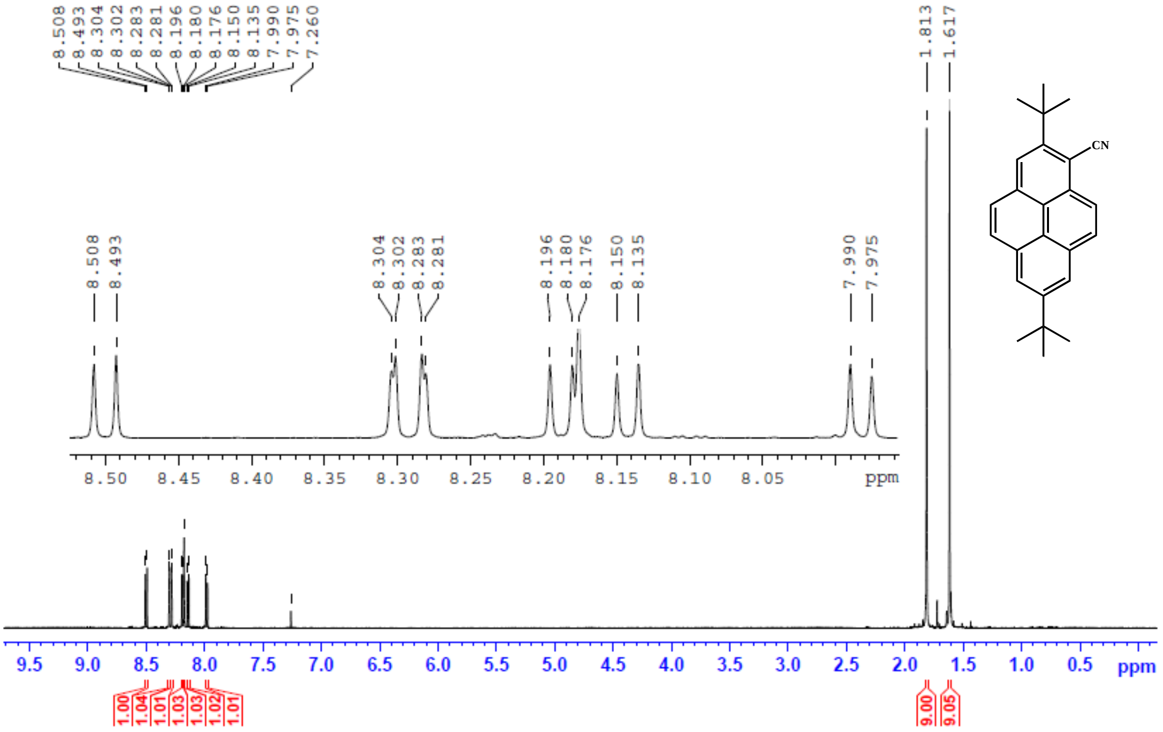

8.508
8.493
8.304
8.302
8.283
8.281
8.196
8.180
8.176
8.150
8.135
7.990
7.975
7.260
1.813
1.617
CN
ppm
1.00
1.04
1.01
1.03
1.03
1.02
1.01
9.00
9.05

5

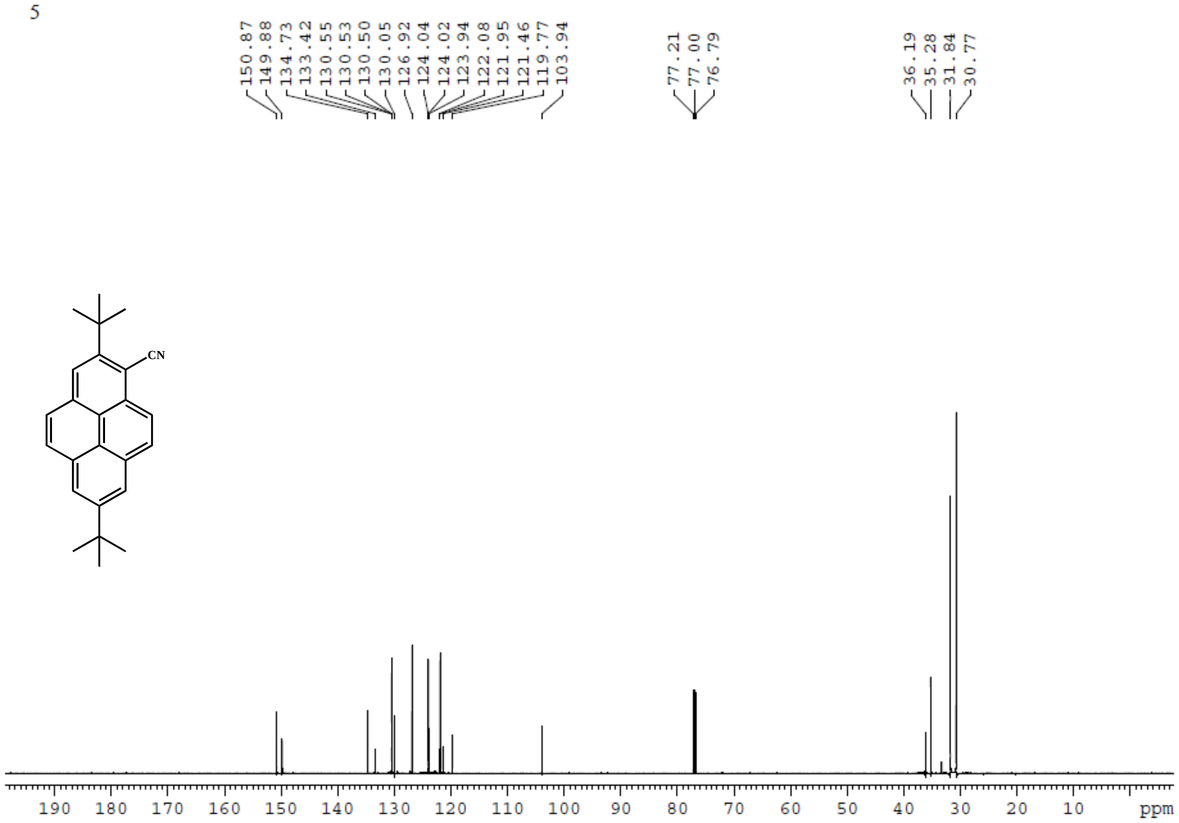
150.87
149.88
134.73
133.42
130.55
130.53
130.50
130.05
126.92
124.04
124.02
123.94
122.08
121.95
121.46
119.77
103.94
77.21
77.00
76.79
36.19
35.28
31.84
30.77
CN
190 180 170 160 150 140 130 120 110 100 90 80 70 60 50 40 30 20 10 ppm

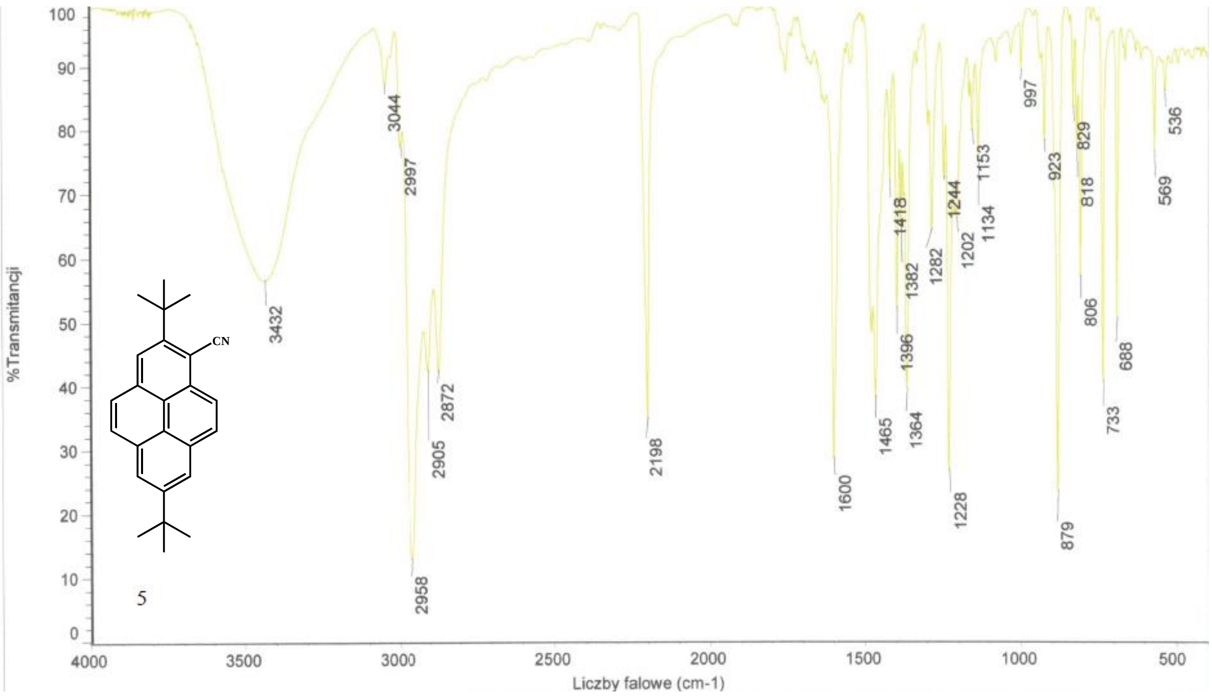
%Transmitancji
Liczby falowe (cm-1)
CN
5
3432
3044
2997
2958
2905
2872
2198
1600
1465
1418
1396
1382
1364
1282
1244
1228
1202
1153
1134
997
923
879
829
818
806
733
688
569
536

6

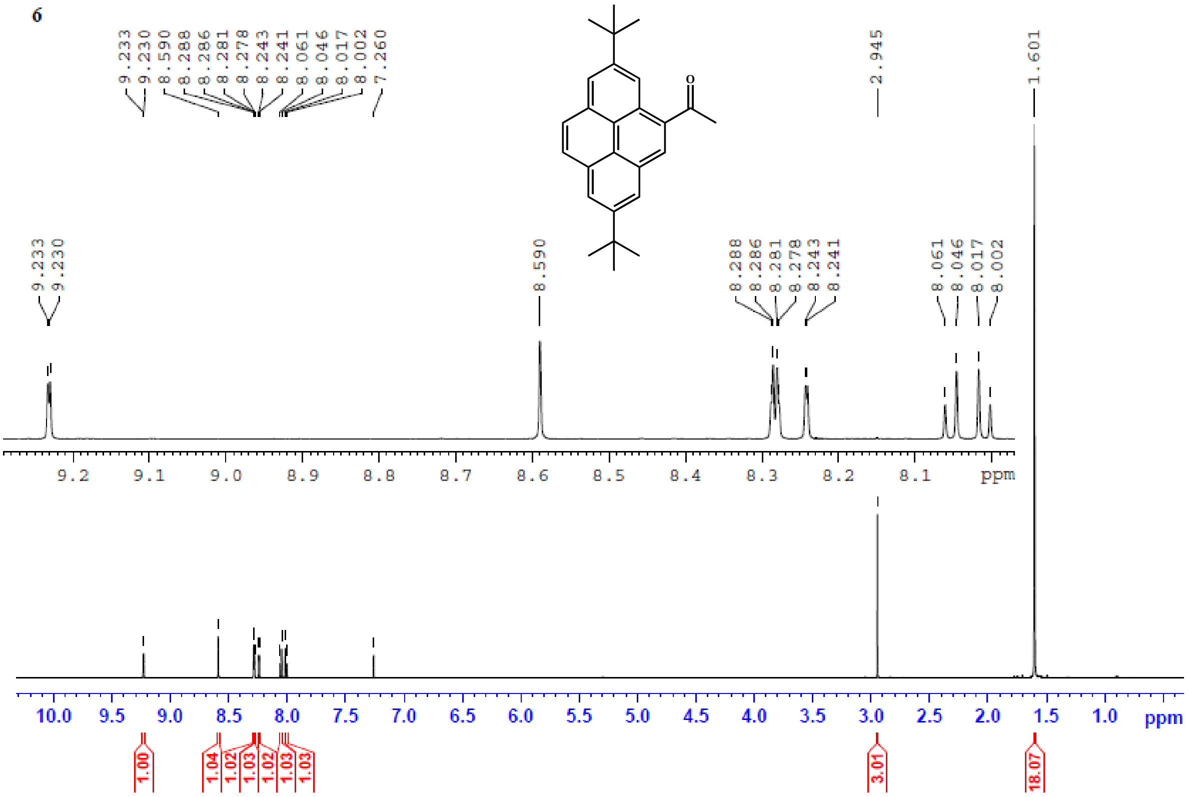
9.233
9.230
8.590
8.288
8.286
8.281
8.278
8.243
8.241
8.061
8.046
8.017
8.002
7.260
2.945
1.601
O
9.2 9.1 9.0 8.9 8.8 8.7 8.6 8.5 8.4 8.3 8.2 8.1 ppm
10.0 9.5 9.0 8.5 8.0 7.5 7.0 6.5 6.0 5.5 5.0 4.5 4.0 3.5 3.0 2.5 2.0 1.5 1.0 ppm
1.00
1.04
1.02
1.03
1.02
1.03
1.03
3.01
18.07

6

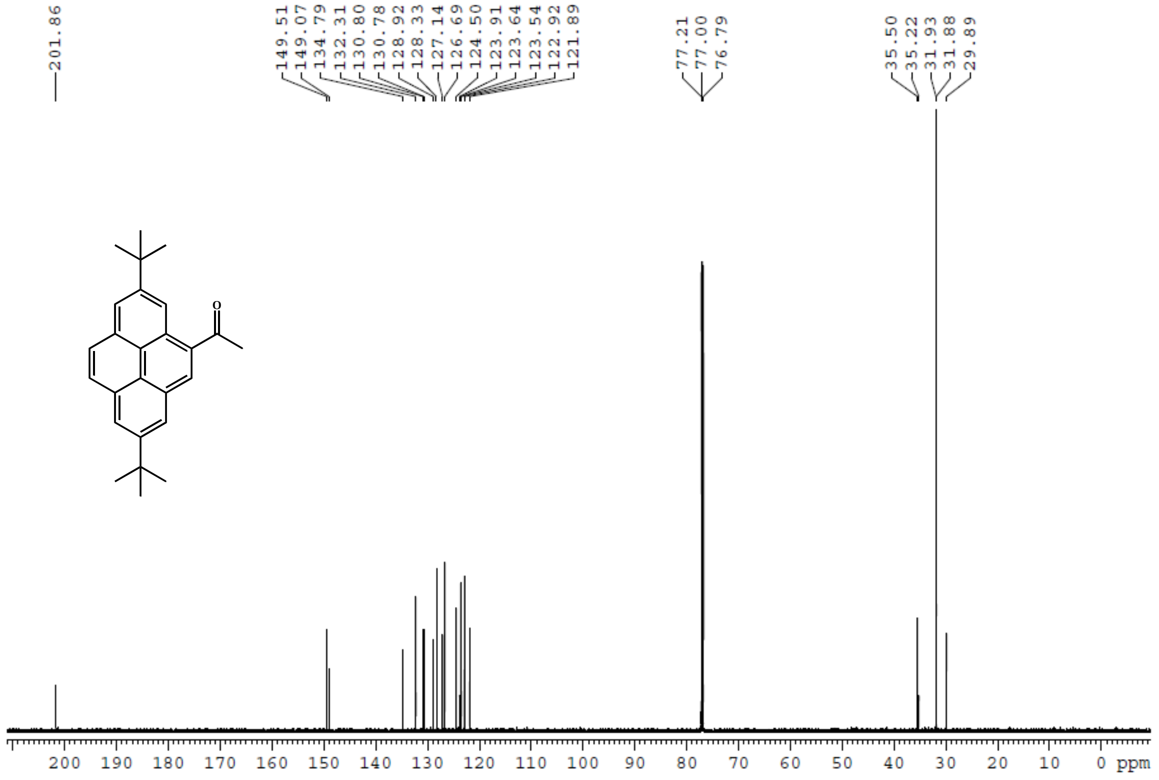
201.86
149.51
149.07
134.79
132.31
130.80
130.78
128.92
128.33
127.14
126.69
124.50
123.91
123.64
123.54
122.92
121.89
77.21
77.00
76.79
35.50
35.22
31.93
31.88
29.89
O
200 190 180 170 160 150 140 130 120 110 100 90 80 70 60 50 40 30 20 10 0 ppm

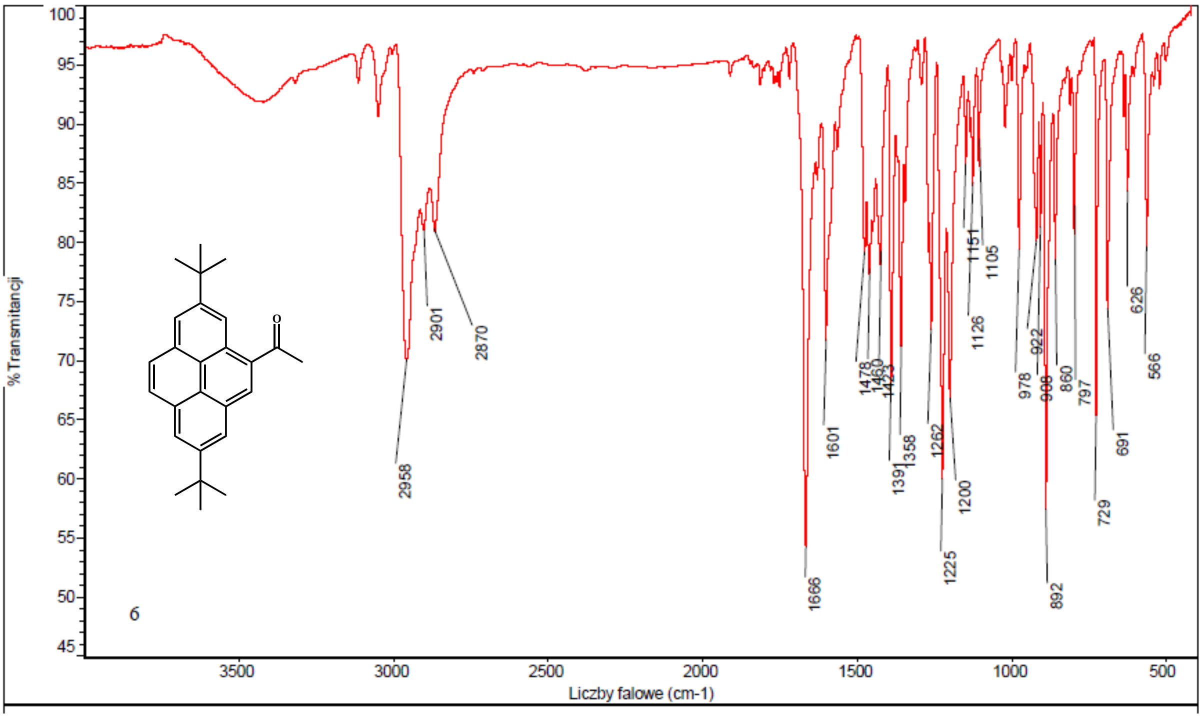
% Transmitancji
Liczby falowe (cm-1)
6
2958
2901
2870
1666
1601
1478
1460
1423
1391
1358
1262
1225
1200
1151
1126
1105
978
922
908
892
860
797
729
691
626
566

Crystallographic and Structure Refinement Data for 4.

Table S1. X-ray crystallographic data for **4**.

**Identification code**	**4**
solvent	CH_2Cl_2
Empirical formula	$C_{32}H_{36}N_2O_4S_2$
Formula weight /gmol^{-1}	589.48
Temperature/K	89.9(2)
Crystal system	monoclinic
Space group	P21/n
a/Å	13.8947(6)
b/Å	11.5388(5)
c/Å	20.3238(10)
α/°	90
β/°	107.084(5)
γ/°	90
Volume/Å^3	3114.7(3)
Z	4
ρ_{calc} mg/mm^3	1.257
F(000)	1249
Θ range for data collection	28.819-1.583
μ /mm^{-1}	0.235
Transmission max/min	1.000
	0.478
Absorption correction type	gaussian
Crystal color	yellow
Crystal habit	needle
Crystal size max/mid/min /mm^3	0.3163
	0.1705

	0.0572
Rint	0.0765
Rsigma	0.0312
Completeness	0.999
Diffractometer / detector	Supernova / Eos
Radiation / wavelength /Å	MoKα 0.71073
Friedel pairs coverage	n/a
Reflections collected I>=2σ (I)	6718
Reflections collected	11128
Largest diff. peak/hole/rms /eÅ^{-3}	0.661
	-0.544
	0.102
Flack parameter	n/a
Extinction coefficient	n/a
Goodness-of-fit on F^2	0.963
Parameters	448
Data	11128
restraints	114
Final R1 indexes [I>=2σ (I)]	0.1025 / 0.0676
Final wR2 indexes [I>=2σ (I)]	0.1721 / 0.1630

Diffraction data were collected on Agilent Supernova 4 circle diffractometer system equipped with molybdenum microsource (Kα, 0.71073Å) and EOS CCD detector. The data were collected withCrysAlis171[1] software and integrated with the CrysAlisPRO[2] software. Data were corrected for absorption effects using the numerical method (SCALE3 ABSPACK).
All the crystals of **4a** were twinned, with two components and the initial twin fractions estimated to be 0.54 and 0.46 for the collected data.

The structure was solved by direct methods using SXELXS[3] and refined by full-matrix least squares procedure with SHELXL[3] within OLEX2[4] graphical interface. Figures were produced

with Mercury_3.5[5]. Most of the H atoms were visible in the residual density map, but all were finally added geometrically and refined in riding approximation. The structure contains solvent molecule with partial occupation, disordered over 2 positions close to the crystallographic symmetry... This results in disorder affecting the **4a** molecule. Each of the two ethyl fragments adapt two alternative positions, with occupancies of 0.65 for the major conformer and 0.35 for the minor.

Additional restraints were necessary in order to properly model the disorder of the ethyl groups and the solvent molecules. The applied restraints were similarity restraints between the major and minor conformers, concerning C-C distances and atomic displacement parameters.

Crystallographic data for 4 have been deposited at the Cambridge Crystallographic Data Centre (Deposition No. 1517030). Copies of these data can be obtained free of charge via www.ccdc.cam.ac.uk/conts/retrieving.html or from the Cambridge Crystallographic Data Centre, 12, Union Road, Cambridge CB21EZ, UK. [Fax: (+44) 1223-336-033; or e-mail: deposit@ccdc.cam.ac.uk].

References

1.CrysAlisPro, Agilent Technologies, Version 1.171.36.20 (release 01-02-2013 CrysAlis171.NET)

2. CrysAlisRED, Agilent Technologies, Version 1.171.36.20 (release 01-02-2013 CrysAlis171.NET)

3. SHELX: G. Sheldrick, A short history of SHELX, Acta Cryst. Section A, *64* (2008) 112.

4. OLEX2: O.V. Dolomanov, L.J. Bourhis, R.J. Gildea, J.A.K. Howard, H. Puschmann, OLEX2: a complete structure solution, refinement and analysis program, J.Appl. Cryst., *42* (2009), 339.

5 MERCURY: C. F. Macrae, P. R. Edgington, P. McCabe, E. Pidcock, G. P. Shields, R. Taylor, M. Towler and J. van de Streek, *J. Appl. Cryst.*, *39* (2006), 45.

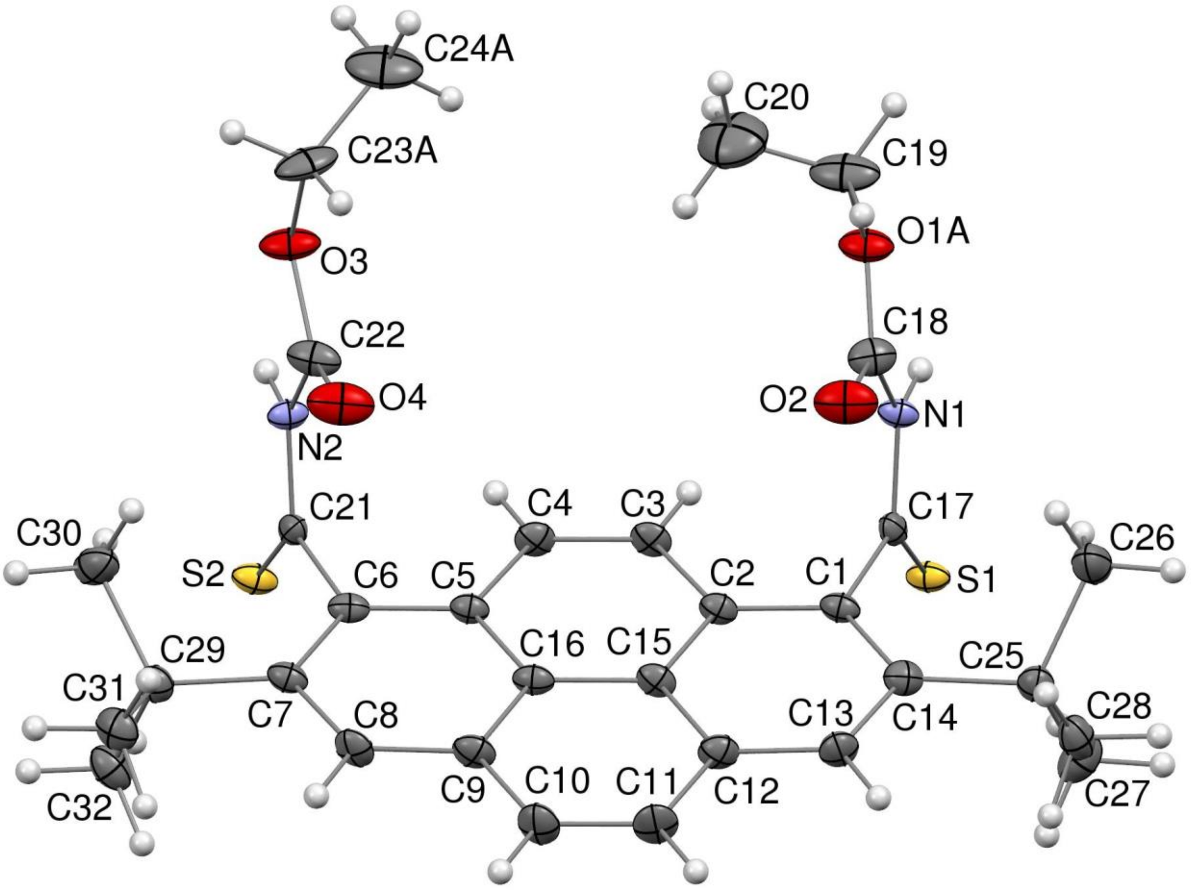

Figure S1. X-ray crystallographic structure of **4** (50% probability level). Only major conformer of the disorder is shown, for clarity.
